# Recent origin and rapid speciation of Neotropical orchids in the world's richest plant biodiversity hotspot

**DOI:** 10.1111/nph.14629

**Published:** 2017-06-20

**Authors:** Oscar Alejandro Pérez‐Escobar, Guillaume Chomicki, Fabien L. Condamine, Adam P. Karremans, Diego Bogarín, Nicholas J. Matzke, Daniele Silvestro, Alexandre Antonelli

**Affiliations:** ^1^ Identification and Naming Department Royal Botanic Gardens, Kew Richmond TW9 3AB UK; ^2^ Systematic Botany and Mycology University of Munich (LMU) 67 Menzinger Str. Munich 80638 Germany; ^3^ CNRS UMR 5554 Institut des Sciences de l'Evolution (Université de Montpellier) Place Eugène Bataillon 34095 Montpellier France; ^4^ Lankester Botanical Garden University of Costa Rica PO Box 302‐7050 Cartago Costa Rica; ^5^ Naturalis Biodiversity Center Leiden 2333 CR the Netherlands; ^6^ Division of Ecology, Evolution, and Genetics Research School of Biology The Australian National University Canberra ACT 2601 Australia; ^7^ Department of Biological and Environmental Sciences University of Gothenburg 413 19 Gothenburg Sweden; ^8^ Department of Computational Biology, Biophore University of Lausanne 1015 Lausanne Switzerland; ^9^ Gothenburg Global Biodiversity Centre Box 461 SE‐405 30 Göteborg Sweden; ^10^ Gothenburg Botanical Garden Carl Skottsbergs gata 22A 41319 Gothenburg Sweden

**Keywords:** Andes, biodiversity hotspots, biogeography, diversification, molecular clocks, mountain building, neotropics, Orchidaceae

## Abstract

- The Andean mountains of South America are the most species‐rich biodiversity hotspot worldwide with *c*. 15% of the world's plant species, in only 1% of the world's land surface. Orchids are a key element of the Andean flora, and one of the most prominent components of the Neotropical epiphyte diversity, yet very little is known about their origin and diversification.
- We address this knowledge gap by inferring the biogeographical history and diversification dynamics of the two largest Neotropical orchid groups (Cymbidieae and Pleurothallidinae), using two unparalleled, densely sampled orchid phylogenies (including more than 400 newly generated DNA sequences), comparative phylogenetic methods, geological and biological datasets.
- We find that the majority of Andean orchid lineages only originated in the last 20–15 million yr. Andean lineages are derived from lowland Amazonian ancestors, with additional contributions from Central America and the Antilles. Species diversification is correlated with Andean orogeny, and multiple migrations and recolonizations across the Andes indicate that mountains do not constrain orchid dispersal over long timescales.
- Our study sheds new light on the timing and geography of a major Neotropical diversification, and suggests that mountain uplift promotes species diversification across all elevational zones.

## Introduction

Species richness is unevenly distributed in time (Simpson, 1953), space (Willis, 1922) and across the Tree of Life (Vargas & Zardoya, 2014). An understanding of the processes underlying current patterns in species richness and distribution therefore constitutes a major scientific challenge. The Andean mountains of South America contain *c*. 15% of the world's plant species, in only 1% of the world's land surface, resulting in the most species‐rich biodiversity hotspot worldwide (Myers *et al*., 2000). A large proportion of this diversity is found in high‐altitude grasslands, and is suggested to have resulted from recent rapid speciation events (Hughes & Eastwood, 2006; Hughes & Atchison, 2015). By contrast, Andean seasonally dry forests experienced much slower diversification and have older origins (Pennington *et al*., 2010), suggesting contrasted macroevolutionary histories within the Andean biodiversity hotspot (Valencia *et al*., 1994; Pennington *et al*., 2010; ter Steege *et al*., 2013).

In a seminal paper, Gentry (1982) postulated that mountain uplift was a major trigger of Andean mega‐diversity, although he posited that this might have occurred indirectly via biotic interactions. A pivotal result of Gentry's floristic analyses was the discovery of two patterns of plant distribution in the Neotropics: ‘Amazonian‐centred’ and ‘Andean‐centred’ taxa. Amazonian‐centred taxa consist mostly of canopy trees and lianas, whereas Andean‐centred taxa are almost exclusively epiphytes and shrubs (Gentry, 1982). The latter occur mostly in the Northern Andes, with secondary centres in the Brazilian coastal mountains and Central America, together accounting for *c*. 33% of all Neotropical plants (Gentry, 1982), and thus largely contributing to the world's most species‐rich biodiversity hotspot, the tropical Andes (Myers *et al*., 2000).

Contrasting with the dominant views at the time, Gentry (1982) hypothesized that the Andean‐centred flora resulted from ‘recent, very dynamic speciation’, a hypothesis that we test here. Gentry & Dodson (1987) further suggested that the high diversity of epiphytes in the Northern Andes and southern South America could have resulted from the finer niche partitioning in these forests, allowing for high alpha diversity, the high microsite differentiation of mountain areas, fostering high beta diversity, and explosive speciation driven by genetic founder effects because of the environmental dynamicity, implying frequent relocation.

Orchids are one of the most characteristic and diverse components of the Andean flora (Gentry & Dodson, 1987; Krömer & Gradstein, 2003; Richter *et al*., 2009; Parra‐Sánchez *et al*., 2016). They often make up 30–50% of the total epiphytic species number reported along the Northern Andes (Kreft *et al*., 2004; Küper *et al*., 2004), and epiphytic orchids account for 69% of all vascular epiphytes world‐wide (Zotz & Winkler, 2013). Neotropical epiphytic orchids are generally characterized by narrowly restricted populations with small numbers of individuals (Tremblay & Ackerman, 2001; Jost, 2004; Crain & Tremblay, 2012; Pandey *et al*., 2013). Despite the ecological importance and prominence of epiphytic orchids (and of epiphyte diversity overall) in the Andean flora, their origin and diversification have not been explicitly studied because of the difficulties in generating densely sampled and strongly supported phylogenies.

We address these issues by studying the evolutionary history of the two largest Neotropical orchid clades, namely Cymbidieae and Pleurothallidinae. The Cymbidieae comprise over 3700 species, 90% of which occur in the Neotropics (the remaining species occur in tropical Africa and Australasia). Cymbidieae comprise 12 subtribes, four of which are the most speciose and include Andean‐dwelling subclades (i.e. Maxillariinae, Oncidiinae, Stanhopeinae and Zygopetalinae; Pridgeon *et al*., 2009). Pleurothallidinae comprise 44 genera and 5100 exclusively Neotropical species (Karremans, 2016) distributed mostly in the highlands of the Northern Andes and Central America. Together, they are the most representative elements of the Andean orchid flora (Pérez‐Escobar *et al*., 2009; Pridgeon *et al*., 2009; Kolanowska, 2014) and make up most of their species richness. In addition, these lineages have evolved a rich array of pollination syndromes and mating systems (including protandry, unisexuality, cleistogamy; Gerlach & Schill, 1991; Borba *et al*., 2011; Pérez‐Escobar *et al*., 2016a) that have long fascinated botanists and naturalists (Lindley, 1843; Darwin, 1877). This is particularly true for Cymbidieae, in which up to seven pollination syndromes have been recorded (van der Cingel, 2001; Pridgeon *et al*., 2009), ranging from species exclusively pollinated by male euglossine bees (Ramírez *et al*., 2011) to those pollinated only by oil bees. Data on the pollination ecology of Pleurothallidinae are very scarce, but scattered reports across the clade suggest that they are mostly pollinated by a vast array of dipteran lineages (Blanco & Barboza, 2005; Pupulin *et al*., 2012).

Rapid Andean orogeny could have promoted orchid species richness by creating ecological opportunities, such as increasing the landscape, mediating local climate change, creating novel habitats and forming insular environments that affected migrations and allopatric speciation through isolation (Gentry & Dodson, 1987; Hoorn *et al*., 2013). This effect should have been most accentuated over the last 10 million yr (Ma), during which *c*. 60% of the current elevation of the Andes was achieved (Gregory‐Wodzicki, 2000). Diversification studies of Andean‐centred clades have provided evidence for rapid diversification that temporally matches the Andean surface uplift, for instance in the plant genera *Lupinus*,* Espeletia*,* Halenia* and *Heliotropium*, and in the families Campanulaceae and Annonaceae (von Hagen & Kadereit, 2003; Bell & Donoghue, 2005; Donoghue & Winkworth, 2005; Hughes & Eastwood, 2006; Pirie *et al*., 2006; Antonelli *et al*., 2009b; Luebert *et al*., 2011; Drummond *et al*., 2012; Madriñán *et al*., 2013; Lagomarsino *et al*., 2016; Diazgranados & Barber, 2017). Taken together, these studies suggest that rapid Andean uplift yielded new niches that fostered both adaptive and non‐adaptive radiations (Nevado *et al*., 2016). Other Andean groups, such as hummingbirds, diversified mostly before Andean uplift (McGuire *et al*., 2014) or after it had attained most of its current height (Smith *et al*., 2014).

We address the impact of the Andean uplift on the diversity and distribution of orchids by inferring the dynamics of speciation, extinction and migration, whilst simultaneously incorporating surface uplift of the two largest Andean Neotropical orchid clades Cymbidieae and Pleurothallidinae. We rely on model‐based inference methods in historical biogeography, ancestral area and character estimation approaches, and a series of diversification analyses to investigate the following questions. From which geographical area(s) do Andean orchids mostly originate? Is there evidence for the Andes acting as a dispersal barrier for epiphytic lowland taxa? Did the Andean uplift enhance orchid diversification and, if so, was this effect evident on all species from the Andean region or just those from the highest elevations? Is Andean diversity derived from pre‐adapted (i.e. high elevation) lineages or rather descendants of lowland migrants (either local or from other areas)? In addition, we use the limited available data to evaluate whether shifts in pollination syndromes are associated with changes in diversification rates.

Our results support Gentry's prediction (Gentry, 1982) that Andean‐centred groups have resulted from recent rapid speciation, suggesting that Andean orogeny provided opportunities for rapid orchid species diversification in the world's premier plant biodiversity hotspot. Such diversity is derived from lowland lineages but, more rarely, from migrants already pre‐adapted to cool environments, a more frequent situation documented from other mountain environments (Merckx *et al*., 2015).

## Materials and Methods

### Taxon sampling, DNA sequencing and phylogenetic analysis

To generate solid phylogenies of the tribe Cymbidieae and subtribe Pleurothallidinae, we newly generated a total of 420 sequences of the nuclear ribosomal internal transcribed spacer (ITS) and a *c*. 1500‐bp fragment of the gene ycf 1 of underrepresented lineages of key biogeographical importance. DNA amplification, PCR product purification and sequencing were conducted as described previously in Irimia *et al*. (2014) and Pérez‐Escobar *et al*. (2016a). Voucher information and GenBank accession numbers are provided in Supporting Information Tables S1 and S2.

We merged our novel dataset with previously generated data from the studies of Blanco *et al*. (2007), Whitten *et al*. (2014), Karremans *et al*. (2016a,b), Pérez‐Escobar *et al*. (2017), and Ramírez *et al*. (2011), using the R‐package megaptera v.1.0 (available at https://github.com/cran/megaptera.git). We retrieved 3541 sequences of nuclear (ITS) and plastid (*mat*K, *trn*L‐F region, *psb*A, *ycf*1). We selected outgroup taxa representing the old and new world subtribes Polystachyinae, Aeridinae and Laeliinae. Trees were rooted on *Calypso bulbosa* (for Cymbidieae) and *Arpophyllum giganteum* (for Pleurothallidinae) following Whitten *et al*. (2014).

Poorly aligned positions were excluded from the alignments using Gblocks v.0.9 (Talavera & Castresana, 2007). To statistically detect potential incongruences between plastid and nuclear DNA phylogenies, we used the tool Procrustes Approach to Cophylogeny (PACo; http://www.uv.es/cophylpaco/) (Balbuena *et al*., 2013; Pérez‐Escobar *et al*., 2016b). Maximum likelihood (ML) tree inference was performed using RAxML‐HPC v.8.0 (Stamatakis, 2014), under the GTR + G substitution model with four gamma categories (best model for both datasets as inferred via the Akaike information criterion (AIC) in jModelTest v.2.1.6; Darriba *et al*., 2012), with 1000 bootstrap replicates and data partitioning by genome compartment. All phylogenetic and dating analyses were performed in the CIPRES Science Gateway computing facility (Miller *et al*., 2015).

### Molecular clock dating

A few unambiguous orchid macrofossils are available for Orchidaceae (*Dendrobium winikaphyllum*,* Earina fouldenensis*,* Meliorchis caribea*; Ramírez *et al*., 2007; Conran *et al*., 2009), but these are assigned to lineages very distantly related to our groups of interest. Using distant outgroups to calibrate our Cymbidieae and Pleurothallidinae phylogenies would have created extensive sampling heterogeneities, which can result in spurious results (Drummond & Bouckaert, 2014). Thus, we had to rely on secondary calibrations. In order to obtain the best secondary calibration points possible, we first generated an Orchidaceae‐wide, fossil‐calibrated phylogeny, sampling 316 orchid species and four loci (ITS, *mat*K, *rbc*L and *trn*L‐F), sampled as evenly as possible along the tree. Detailed settings and fossil calibrations used to generate an Orchidaceae‐wide phylogeny are provided in the extended Methods S1.

Secondary calibration points were obtained from our Orchidaceae‐wide dated phylogeny, and the most recent common ancestor (MRCA) of Cymbidieae + Vandeae was dated to 34 ± 7 Ma, 95% credible interval (CI), whereas that of Pleurothallidinae + Laeliinae was estimated to 20 ± 7 Ma. We therefore used a normal prior (with values of mean = 34, SD = 4 for Cymbidieae; mean = 20, SD = 3 for Pleurothallidinae, to reflect the 95% CI from our fossil‐calibrated tree) to calibrate our phylogenies using these secondary constraints, which were designed to reflect the uncertainty previously estimated for the root node of Cymbidieae and Pleurothallidinae.

### Ancestral range estimation

Species ranges were coded from the literature (Pridgeon *et al*., 2009) and from herbarium specimens through a survey of virtual collections and loans of several herbaria (AMES, COL, F, MO, SEL, US, M), as well as the Global Biodiversity Information Facility (GBIF) repository. To query the GBIF database, we relied on the function *occ* of the R‐package spocc (Chamberlain *et al*., 2016). A total of 19 486 distribution records were compiled for the Cymbidieae, and 9042 records for the Pleurothallidinae. Protocols for distribution maps and species richness pattern analyses are detailed in Methods S1.

**Figure 1:**
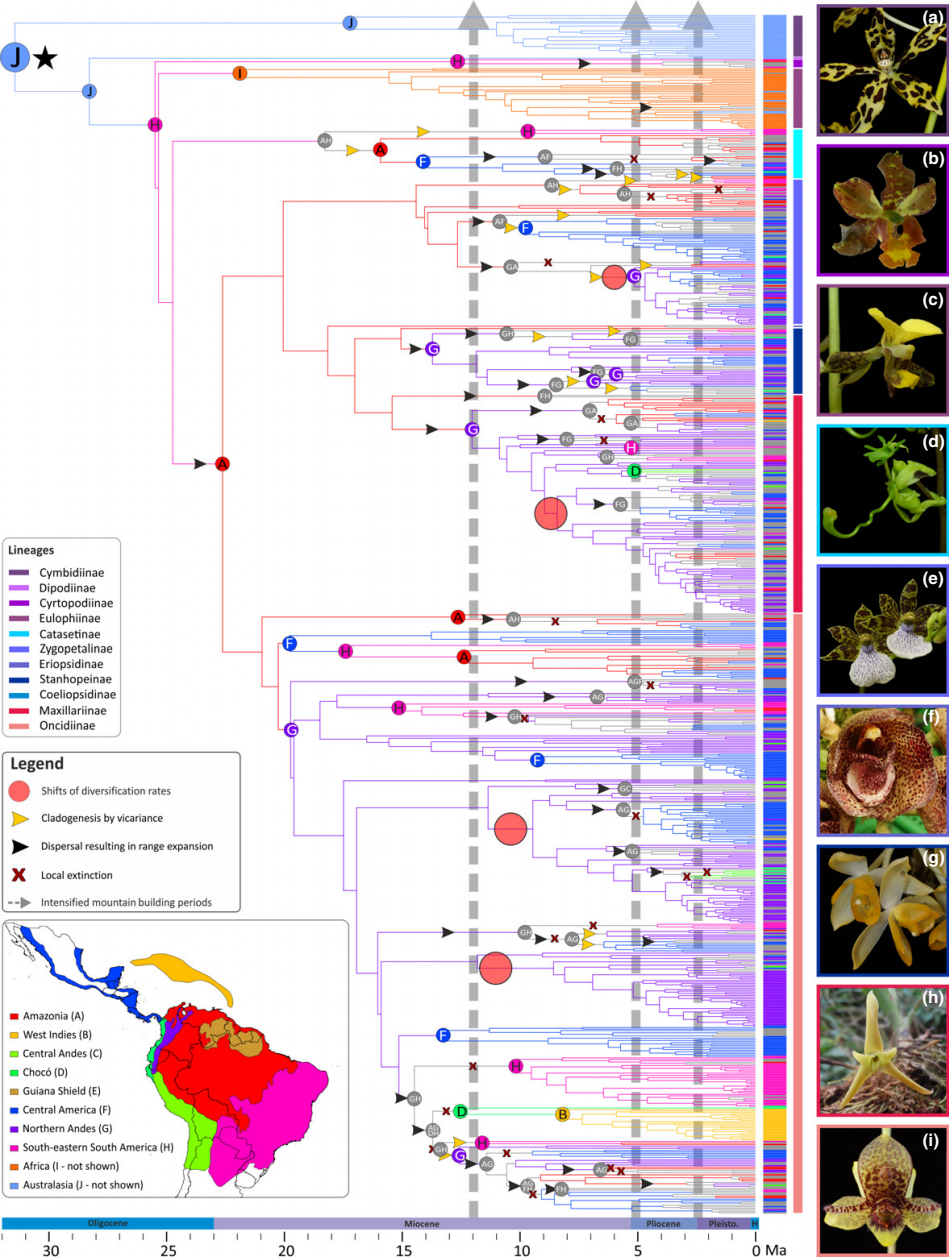
Biogeographical history of Cymbidieae orchids. Letters on the coloured circles at the nodes indicate the estimated ancestral area with the highest probability as inferred by BioGeoBears. Branches are colour coded following the reconstructed area of their corresponding node, and the geographical ranges of every taxon are shown as vertical bars in front of the terminals. The black star indicates the most recent common ancestor of Cymbidieae. Grey arrows show the periods of accelerated Andean uplift (Gregory‐Wodzicki, 2000). Changes on shifts of diversification rates are shown as pale red circles on the branches. Range expansions, local extinctions and cladogenetic events via vicariance are indicated on the branches with black and yellow arrowheads and red crosses, respectively. Subtribe members of Cymbidieae are colour coded. Right panels show selected representatives of (a) Cymbidiinae (*Grammatophyllum measuresianum*); (b) Cyrtopodiinae (*Cyrtopodium macrobulbon*; photograph by D. Bogarín); (c) Eulophiinae (*Eulophia streptopetala*); (d) Catasetinae (*Cycnoches egertonianum*); (e) Zygopetalinae (*Zygopetalum* aff. *brachypetalum*); (f) Coeliopsidinae (*Peristeria cerina*); (g) Stanhopeinae (*Sievenkingia* sp.); (h) Maxillariinae (*Cryptocentrum* sp.); (i) Oncidiinae (*Trichoceros* sp.). Photographs (except b): O. Pérez. (Inset) Coded areas for biogeographical analysis. Political divisions obtained from DIVA‐GIS (http://www.diva-gis.org/gdata). Timescale shown at bottom is expressed in million years ago (Ma).

**Figure 2:**
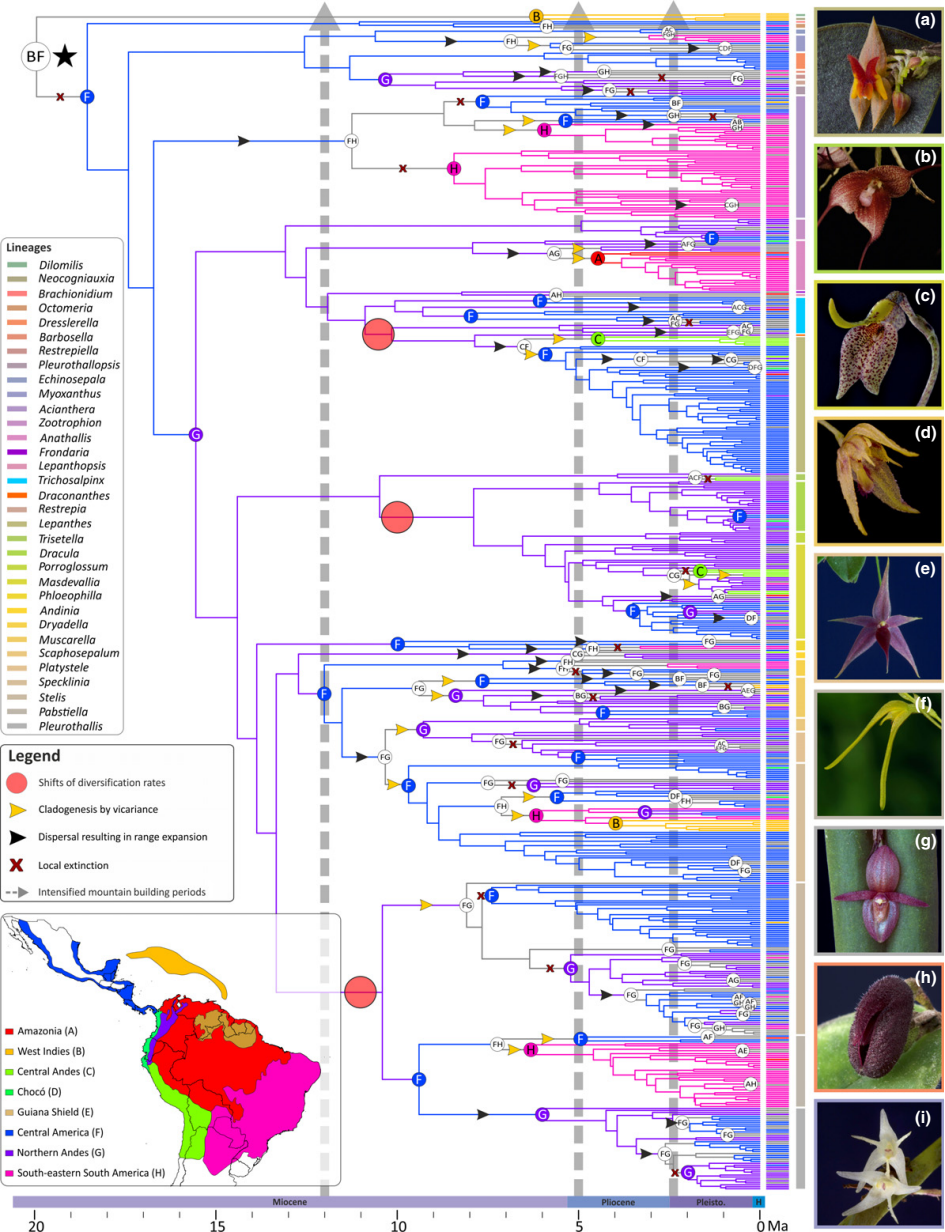
Biogeographical history of Pleurothallidinae orchids. Letters on coloured circles at the nodes indicate the estimated ancestral area with the highest probability as inferred by BioGeoBears. Branches are colour coded following the reconstructed area of their corresponding node, and geographical ranges of every taxon are shown as vertical bars in front of the terminals. The black star indicates the most recent common ancestor of Pleurothallidinae. Grey arrows show the periods of accelerated Andean uplift (Gregory‐Wodzicki, 2000). Changes on shifts of diversification rates are shown as pale red circles on the branches. Range expansions, local extinctions and cladogenetic events via vicariance are indicated on the branches with black and yellow arrowheads and red crosses, respectively. Generic members of Pleurothallidinae are colour coded. Right panels show selected representatives of (a) *Lepanthes* (*Lepanthes* sp.); (b) *Dracula* (*D. astuta*); (c) *Masdevallia* (*M. utriculata*); (d) *Muscarella* (*M. exesilabia*); (e) *Platystele* (*P. porquinqua*); (f) *Pabstiella* (*P. ephemera*); (g) *Pleurothallis* (*P. adventurae*); (h) *Dresslerella* (*D. pilosissima*); (i) *Myoxanthus* (*M. colothrix*). Photographs: A. Karremans, D. Bogarín and O. Pérez. (Inset) Coded areas for biogeographical analysis. Political divisions obtained from DIVA‐GIS (http://www.diva-gis.org/gdata). Timescale shown at bottom is expressed in million years ago, Ma.

Distribution maps for Cymbidieae and Pleurothallidinae (summarized in Figs S1, S2) and extant distribution patterns identified for other plant lineages (e.g. Rubiaceae, Antonelli *et al*., 2009b) allowed the identification of 10 main distribution areas (see the inset in Figs 1, 2). Species were assigned to one of these regions: Central America (comprising southern Florida to Panama); West Indies (i.e. Caribbean Islands); Northern Andes (mountain ranges from elevations higher than 500 m in Colombia and Venezuela); Central Andes (from Peru to the Tropic of Capricorn, from elevations higher than 500 m); Amazonia (including lowlands and montane forest below 500 m in Colombia, Ecuador, Peru, Brazil, Venezuela, Guyana, Suriname and French Guiana); the Guiana Shield (including elevations higher than 500 m in north‐eastern South America (Brazil, Guyana, Suriname and Venezuela)); South‐eastern South America (including the Brazilian shield, but also lowlands in eastern Brazil and northern Argentina); Chocó (comprises lowlands below 500 m of the western Andes in Colombia and Ecuador); Africa; and Australasia. To infer the ancestral range of all examined lineages in Cymbidieae and Pleurothallidinae, we used the R‐package BioGeoBears v.0.2.1 (Matzke, 2013, 2014). In addition, in order to estimate the number of migrations, dispersals, extinctions and within‐area speciation events from our phylogeny, we used biogeographical stochastic mapping (BSM) (Matzke, 2014) under the best‐fit model, as implemented in BioGeoBears (for detailed settings, see Methods S1).

### Rates of species diversification

To infer the diversification dynamics of the Cymbidieae and Pleurothallidinae, we first used a time‐dependent model implemented in Bamm v.2.5.0 (Rabosky, 2014) to estimate the rates of extinction and speciation across the phylogenies. Incomplete taxon sampling was accounted for by assigning a sampling fraction of 25% of the extant orchid diversity of Cymbidieae, and 13% of Pleurothallidinae (sampling fractions of every genus sampled were incorporated according to Chase *et al*., 2015). We performed three runs with 1 million Markov chain Monte Carlo (MCMC) generations, sampling parameters every 10 000 generations. Diversification rates and rate shift configurations were plotted using the R‐package BAMMtools (Rabosky *et al*., 2014). We checked the convergence of the runs by plotting the log‐likelihood across MCMC generations sampled in the ‘mcmc_out’ file. To evaluate the best model generated by Bamm (compared with a null *M*_0_ model with no diversification rate shifts), we relied on Bayes Factors calculated with the ComputeBayesFactor function of BAMMtools. We examined the 95% credible set of macroevolutionary shift configurations using the BAMMtools function CredibleShiftSet. We sought cross‐validation of our Bamm results with Rpanda (Morlon *et al*., 2016), and details about the settings are provided in Methods S1.

### Geographical state‐dependent analyses

We used GeoSSE (Goldberg *et al*., 2011), an extension of the BiSSE model that allows lineages to occur simultaneously in two areas and to test whether one area has overall higher speciation rates, as implemented in the R‐package Diversitree v.0.9‐7 (Fitzjohn, 2012). To test whether lineages restricted to the Northern Andes (‘A’) had higher diversification rates than lineages absent from the Northern Andes (collectively called ‘B’ here), we used Bayesian MCMC GeoSSE analyses of 1 million generations on the maximum clade credibility tree from Beast (in the particular case of Cymbidieae, only Neotropical representatives were included). Implemented models in GeoSSE and settings of tailored simulations to account for Type I error biases in GeoSSE are provided in Methods S1.

### Mapping speciation rates in the Neotropics

Based on the speciation and extinction rates inferred for orchid lineages, and their geographical occurrence, it is possible to identify important areas of diversification as plotted on a heat map (Condamine *et al*., 2013). For this purpose, we designed a novel method that involves retrieving speciation rates from Bamm analyses using the function GetTipsRates in BAMMtools v.2.1 (Rabosky *et al*., 2014) and to link them to species occurrences. Rates were further associated to known distribution records of Cymbidieae and Pleurothallidinae and interpolated to a polygon representing the currently known distribution of Cymbidieae and Pleurothallidinae species, using the inverse distance weight method implemented in the software ArcMap v.9.3 (Esri). To account for geographical sampling biases, we divided the geographical range of species records into a grid of 0.5° × 0.5° cells. We then randomly sampled occurrences arrayed on every grid cell using the R package Raster (Hijmans & Elith, 2016), so that a single occurrence per grid cell was kept.

### Palaeo‐elevation‐dependent diversification

We tested the effect of past environmental change on the diversification of Cymbidieae and Pleurothallidinae using birth–death models that allow speciation and extinction rates to vary according to a quantitative, time‐dependent, environmental variable (Condamine *et al*., 2013), here the palaeo‐elevation of the Northern Andes (Hoorn *et al*., 2010; Lagomarsino *et al*., 2016). The R‐package Pspline (Ramsey & Ripley, 2010) was used to interpolate a smooth line for Andean palaeo‐elevation. This smooth line was sampled during each birth–death modelling process to give the value of the palaeo‐elevation variable at each time point. Speciation and extinction rates were then estimated as a function of these values along the time‐calibrated phylogenies, according to the parameters of each model. The palaeo‐environmental‐dependent model is implemented in the R‐package Rpanda v.1.1 (Morlon *et al*., 2016). Implemented models in Rpanda are provided in Methods S1.

### Ancestral character state estimation

To account for potential biotic variables as drivers of Neotropical orchid diversification, such as shifts on pollination syndromes (Givnish *et al*., 2015), we compiled information on the pollination syndromes of Cymbidieae from the literature (van der Cingel, 2001; Singer, 2002; Pansarin *et al*., 2009; Pridgeon *et al*., 2009; Gerlach, 2011; Ramírez *et al*., 2011), and consulted experts on specific groups (see the Acknowledgements section). As a result of a dearth of detailed information on pollination ecology (i.e. available for *c*. 6% of taxa sampled only), we followed a generalist coding approach, and seven pollination syndromes, (i.e. bee, bird, butterfly, lepidopteran, fly, wasp and self‐pollination) were coded. To account for missing information on pollination syndromes, we assigned equal probabilities to all character states to taxa with unknown pollination syndromes. To estimate ancestral elevation ranges in Pleurothallidinae and Cymbidieae, we obtained absolute elevation values from herbarium records for every taxon sampled in our phylogenies. We obtained a mean of five values per taxa sampled, and we coded mean elevation values as a continuous character. We followed the classification of major Andean ecoregions proposed by Rangel‐Churio *et al*. (1997) and Jørgensen & León‐Yánez (1999), and taxa occurring at elevations higher than 1100 m were considered to inhabit sub‐Andean (montane) forests (1100–2400 m). Species occurring at elevations of < 1100 m were considered as lowland inhabitants. Detailed settings for ancestral character state of altitude and pollination syndromes are provided in Methods S1.

## Results

### Phylogenetics, age and biogeography of Andean orchids

Analyses of phylogenetic incongruence detection identified 259 and 125 potential conflicting tips in Cymbidieae and Pleurothallidinae, respectively (Figs S3, S4), all of which clustered in weakly to moderately supported clades (< 75% bootstrap support, BS) or in clades with extremely long branches. These analyses indicated the absence of supported phylogenetic incongruence (Mason‐Gamer & Kellog, 1996; Pérez‐Escobar *et al*., 2016b). In the absence of supported phylogenetic conflicts, nuclear and plastid partitions of Cymbidieae and Pleurothallidinae were concatenated. For the Cymbidieae, our molecular dataset consisted of 6.6 kb DNA (five markers) for 816 species, and yielded the first strongly supported phylogeny of the tribe (Fig. S5). The Pleurothallidinae dataset was composed of 2.4 kb DNA (two markers) and 684 terminals, including, in total, 420 newly generated sequences (Fig. S6). Both orchid phylogenies are strongly supported at most important nodes, with 618 nodes (76%) with BS > 75% for the Cymbidieae, and 321 nodes (47%) with BS > 75% for the Pleurothallidinae (Figs S5, S6).

Ages obtained on our wide orchid‐dated phylogeny were very similar to those of other recent orchid dating studies (Chomicki *et al*., 2015; Givnish *et al*., 2015). A chronogram for the orchid family showing absolute ages and 95% CIs for every node is provided in Fig. S7. The absolute ages obtained for Cymbidieae and Pleurothallidinae chronograms are also in agreement with previously published dated phylogenies (e.g. Ramírez *et al*., 2011; Chomicki *et al*., 2015; Givnish *et al*., 2016). Divergence time estimates and 95% CIs inferred for all nodes of Cymbidieae and Pleurothallidinae chronograms are shown in Figs S8 and S9.

Our dating and biogeographical analyses identified the Dispersal–Extinction–Cladogenesis model with founder speciation event (DEC + J) as the best fitting model for both Cymbidieae and Pleurothallidinae (Tables S3, S4). Under this model, an Australasian origin of the Cymbidieae around the Eocene–Oligocene boundary (34 ± 8 Ma) was inferred (Figs 1, S8, S10). We inferred a late Oligocene dispersal from Australasia to South America following the estimation of southern South America as the ancestral area of *Cyrtopodium* and the rest of the Cymbidieae (Figs 1, S10). Such dispersal corresponds to the final break‐up of Gondwana (split between Antarctica and South America at Drake Passage). From the late Oligocene to the early Miocene, our analyses indicate dispersal from east to west in the Neotropics. The Northern Andean region was reached four times from Amazonia by MRCAs nested in Oncidiinae *c*. 19 ± 5 Ma, Maxillariinae *c*. 11 ± 5 Ma, Stanhopeinae *c*. 13 ± 4 Ma and Zygopetalinae *c*. 5 ± 2 Ma.

Ancestral state estimations of mean altitude further show that the MRCA of Cymbidieae was probably adapted to lowland environments (ancestral elevation value of *c*. 900 m; Figs S11, S12). Three of the MRCAs of Amazonian migrants that reached the Andes (i.e. nested in Maxillariinae, Stanhopeinae and Zygopetalinae) were not pre‐adapted to montane habitats (mean elevation values of *c*. 1050, 900 and 1000 m, respectively (< 1000–1100 to 2400 m; Cuatrecasas, 1958; Rangel‐Churio *et al*., 1997); Figs S11, S12). The MRCA of Oncidiinae that reached the Northern Andes, by contrast, was probably adapted to montane habitats (*c*. 1200 m). Strikingly, Oncidiinae and Maxillarinae are the species‐richest lineages in Cymbidieae (1584 and 819 species, respectively; Chase *et al*., 2015), and are derived from both lowland Amazonian and montane pre‐adapted migrants. Stanhopeinae subsequently dispersed to several other Neotropical regions, particularly Central America (Figs 1, S10).

Different from the Cymbidieae, we infer an origin of Pleurothallidinae in Central America or the West Indies in the early Miocene, followed by a migration to the Northern Andes *c*. 16 ± 5 Ma (Figs 2, S9, S13), before the main uplift periods, but within a timeframe in which the Northern Andes had already achieved peak mean elevations of *c*. 1500 m. However, the majority of early divergent Pleurothallidinae and their sister groups are from the Antilles, and thus the inference of Central America as the ancestral area of Pleurothallidinae most probably reflects our inability to sample extensively the early diverging Antillean lineages. As inferred by ancestral state estimations, the MRCA of Pleurothallidinae was probably adapted to montane habitats (mean elevation of *c*. 1200 m), and all Pleurothallidinae migrants to the Northern Andes were probably adapted to montane–cloud forest environments (mean elevation of *c*. 1200–1300 m; Figs S14, S15). BSM indicates that *in situ* speciation was the dominant biogeographical process in both clades, whereas processes of range expansion (dispersal and vicariance) and range contraction (subset speciation) were scarcer and relatively evenly distributed across lineages (Figs 1, 2, S16, S17).

### Diversification of Andean orchids

**Figure 3:**
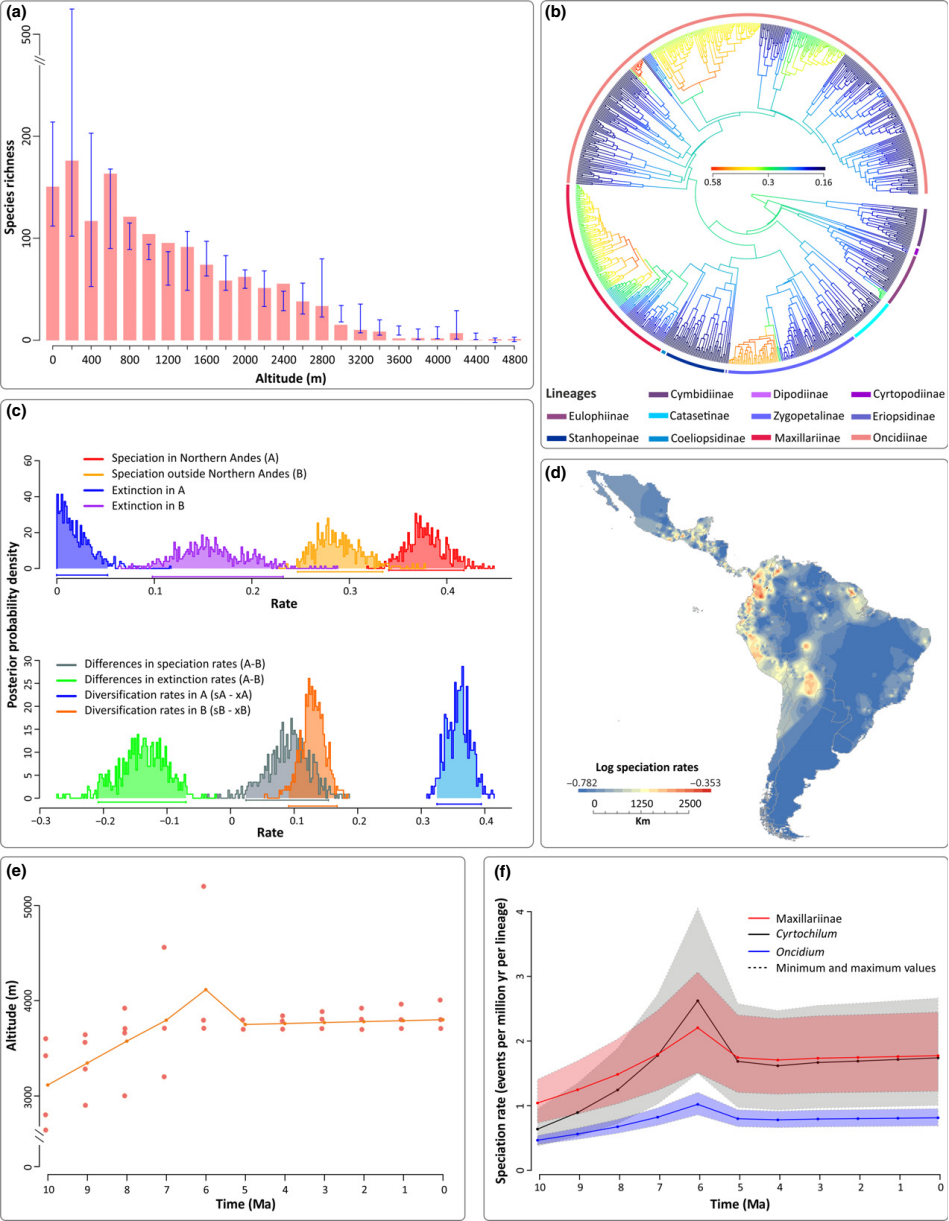
Diversification of the Cymbidieae. (a) Richness vs elevation plot for 55% (> 20 000 herbarium records) of the *c*. 4000 Cymbidieae species. Blue error bars indicate maximum and minimum species richness values. (b) Speciation rate plot (phylorate) showing the best configuration shift identified by Bamm. Colour intensity across branches is proportional to changes in diversification rates. (c) Density probability plots of speciation, extinction and net diversification rates per area identified by Geo SSE. Area ‘A’ refers to species restricted to the Northern Andes; area ‘B’ refers to species occurring in all areas except the Northern Andes. (d) Speciation rate map estimated from Bamm (see the Materials and Methods section). (e) Average palaeo‐elevation of the Central and Northern Andes. (f) Palaeo‐elevation‐dependent models applied to the four clades detected by Bamm to have significantly higher diversification rates than others. Lineages in (b) are colour coded in the same way as shown in Fig. 1. Timescale in panels (e) and (f) is expressed in million years ago (Ma).

**Figure 4:**
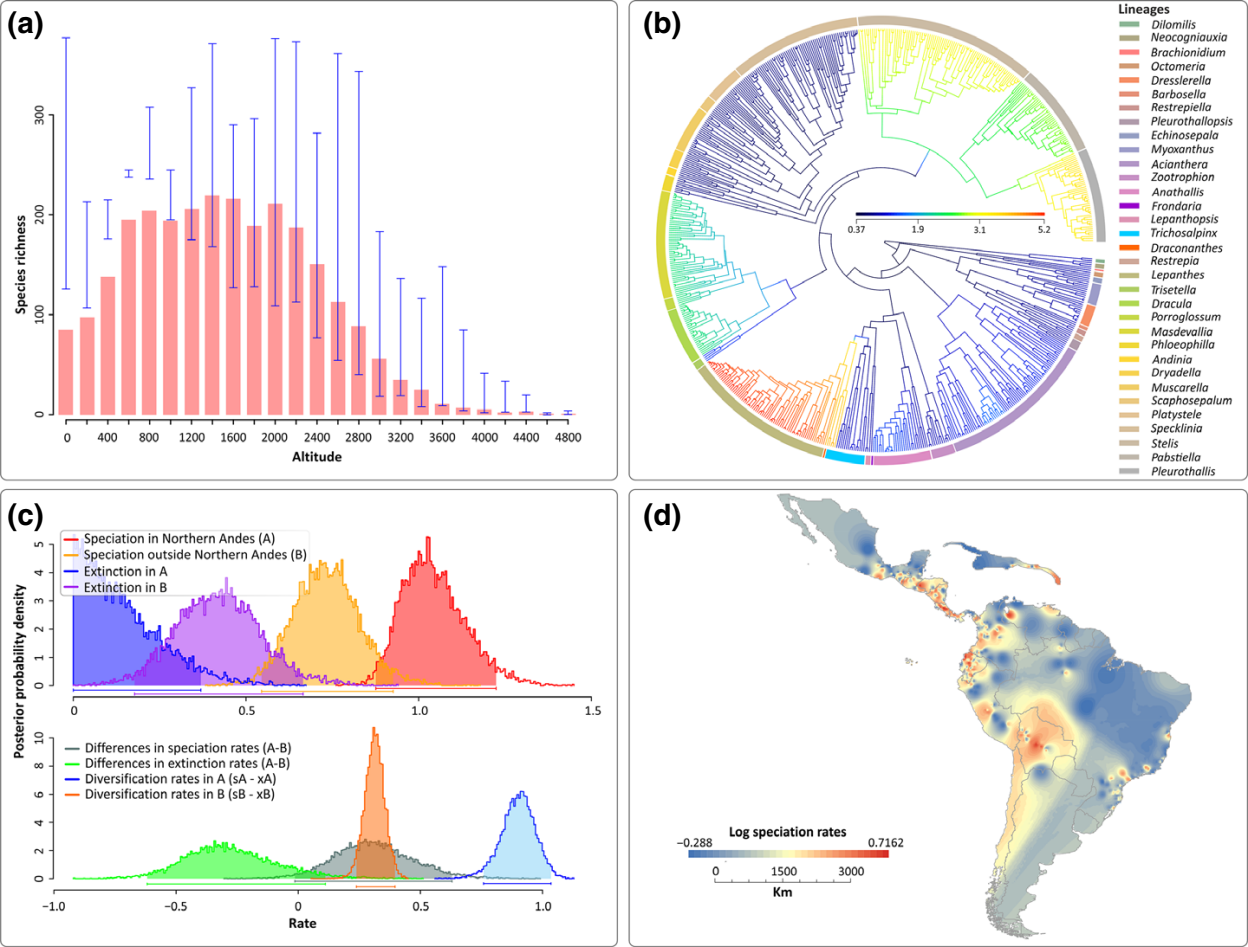
Diversification of the Pleurothallidinae. (a) Richness vs elevation plot for 50% (> 9000 herbarium records) of the *c*. 5000 Pleurothallidinae species. Blue error bars indicate maximum and minimum species richness values. (b) Speciation rate plot (phylorate) showing the best configuration shift identified by Bamm. Colour intensity across branches is proportional to changes in diversification rates. (c) Density probability plots of speciation, extinction and net diversification rates per area identified by Geo SSE. Area ‘A’ refers to species restricted to the Northern Andes; area ‘B’ refers to species occurring in all areas except the Northern Andes. (d) Speciation rate map estimated from Bamm (see the Materials and Methods section). Lineages in (b) are colour coded in the same way as shown in Fig. 2.

The diversification analyses performed with Bamm strongly rejected a constant‐rate model (Bayes factor = 151.3, Table S5) and, instead, identified four rate shifts during the evolutionary history of Cymbidieae (Figs 3b, S18, S19). The best model configuration identified four shifts in speciation rate in the most speciose Cymbidieae lineages: one in Maxillariinae, one in Zygopetalinae and two in Oncidiinae. We further identified three rate shifts in the Pleurothallidinae (Table S6): at the MRCA of *Lepanthes* + *Lepanthopsis*, MRCA of *Dracula* + *Porroglossum* + *Masdevallia*, and MRCA of *Stelis* + *Pabstiella* + *Pleurothallis* (Figs 4b, S20, S21). All shifts in diversification rates in Cymbidieae and Pleurothallidinae were further confirmed using the Rpanda method (Figs S22, S23; Tables S7, S8).

The diversification rate shifts are all located at clades that already inhabited the Northern Andes, and temporally match with periods of accelerated Andean uplift in this region (Cymbidieae, Fig. 1; Pleurothallidinae Fig. 2). To further explore this apparent correlation with either accelerated Andean uplift or presence in the Northern Andes and fast diversification, we used a trait‐dependent approach (GeoSSE) that estimates region‐dependent speciation rates. Here, a model with free rates fitted best our Cymbidieae and Pleurothallidinae datasets (Table S9), indicating significant differences in speciation (sA − sB) and diversification (dA − dB) rates highly if not maximally supported (0.99 and 1 Bayesian posterior probabilities, respectively). GeoSSE analyses further indicated that speciation rates in Northern Andes are consistently higher than in any other biogeographical region (Figs 3c, 4c) in both Cymbidieae and Pleurothallidinae datasets. We evaluated and confirmed the robustness of these results through extensive data simulations (Fig. S24). Here, the null distribution of GeoSSE ∆AIC values obtained from analyses with reshuffled area states was centred towards values of −20 000 and far away from the ∆AIC values obtained under analyses with real area states.

We developed a novel method to generate a ‘speciation rate map’ using inferred speciation rates for each orchid lineage and georeferenced species occurrences (see the Materials and Methods section). Our speciation rate maps are in agreement with GeoSSE results, and we confirmed that speciation rates in the Northern Andes were significantly higher than those in any other region (Figs 3c, 4c). This is in agreement with a recent study with more limited taxon sampling for the two clades focused on here (Givnish *et al*., 2015). The speciation rate map (see the Materials and Methods section) further demonstrates that fastest speciation took place in the Northern Andes region, and reveals secondary speciation hotspots in the Central Andes, the Guiana Shield and Central America (Figs 3d, 4d). These secondary hotspots are occupied by species derived from the four highly diversifying Northern Andean Cymbidieae clades (Fig. S25), suggesting that the Andes acted as a major source of new lineages to the rest of the continent, thus greatly increasing Neotropical orchid diversity. This is particularly true for the Pleurothallidinae, where we identified multiple migrations from the Northern Andes of montane‐adapted lineages to Central America (Figs 2, S26). We also found a strong geographical correlation between current species richness and diversification (Figs 3d, 4d, S27, S28), suggesting that recent *in situ* speciation was the main process for species accumulation in the Neotropics.

Although these results suggest an impact of the Andean uplift on species diversification, they do not explicitly account for biotic interactions, landscape and climatic changes through time. We therefore assessed the fit of a model that explicitly integrates palaeo‐elevation in diversification rate analyses (see the Materials and Methods section). In three of the four Cymbidieae clades in which Bamm inferred a speciation rate shift, the palaeo‐elevation‐dependent model inferred a continuous speciation increase from 10 to 6 Ma as a result of a positive correlation between speciation and palaeo‐elevation (Fig. 3e,f; Table S10). By contrast, no positive correlation with palaeo‐elevation and diversification could be detected for Pleurothallidinae (Table S11). Moreover, our ancestral character estimation of pollination syndromes in Cymbidieae suggests that the MRCA of Cymbidieae was bee pollinated (Fig. S29). Nine shifts of syndromes were identified along the evolutionary history of Cymbidieae, always derived from bee pollination. No reversals from other syndromes towards bee pollination were recovered (Fig. S29).

## Discussion

### Andean orchids are derived from lowland Amazonian, montane Central American and local sub‐Andean migrants

Our ancestral area estimations show that Andean orchid flora is derived primarily from Amazonian lowland taxa (i.e. MRCAs of Andean clades of Maxillariinae, Stanhopeinae and Zygopetalinae, from which most of the species‐richest lineages in Cymbidieae originated), but also from cool pre‐adapted lineages (MRCAs of both Andean Oncidiinae and most extant Andean‐centred pleurothallid taxa). Previous research has revealed that mountain flora origin is strongly influenced by the immigration of cool pre‐adapted lineages (Hughes & Eastwood, 2006; Merckx *et al*., 2015; Uribe‐Convers & Tank, 2015), and that contributions from lowland‐adapted lineages is rather rare. In Borneo, a large portion of the mountain endemics of Mount Kinabalu arose from pre‐adapted lineages from other cool areas (Merckx *et al*., 2015), but *Dendrochilum* orchid montane endemics arose from low‐elevation local ancestors (Barkman & Simpson, 2001). Similarly, epiphytic, tuberous Rubiaceae (Hydnophytinae) endemics from New Guinea montane habitats originated from local lowland migrants (Chomicki & Renner, 2017). Our study points to the key role of Amazonia for the origin of Andean orchid diversity, and also reveals an ancient biological connectivity between Amazonia and the Northern Andes.

### The Andes did not constrain orchid dispersal

The recurrent migration back and forth through the Andes, even during the period of highest palaeo‐elevation, is also a central result from our study. The colonization of the Northern Andes by some clades of Cymbidieae matches in time with accelerated surface uplift (Figs 1, S10), and reflects the Miocene biotic connectivity between the Andes and Amazonia previously suggested for plants (Antonelli *et al*., 2009a), Poison dart frogs (Santos *et al*., 2009), and birds (Brumfield & Edwards, 2007), among others. This suggests that shifts across elevational zones were not rare, contrary to recent results in Mount Kinabalu in Borneo (Merckx *et al*., 2015).

Surprisingly, dispersal events across the Andes did not decrease during accelerated Andean uplift (Figs 1, 2, S10, S13), suggesting that the uplift of the Andes did not act as a major dispersal barrier for Cymbidieae and Pleurothallidinae orchids, contrary to findings in other plant groups (e.g. Annonaceae, Pirie *et al*., 2006; Rubiaceae, Antonelli *et al*., 2009b; or Fabaceae, Pennington *et al*., 2010). This result probably relates to the biology of orchids, which produce large amounts of dust‐like, wind‐dispersed seeds, allowing for occasional long‐distance dispersal (Arditti & Ghani, 2000; Antonelli *et al*., 2009a; Barthlott *et al*., 2014; Givnish *et al*., 2016; Pérez‐Escobar *et al*., 2017), enabling occasional crossing of the Andes, and perhaps more frequently migration to different elevation zones. Taken together, these findings suggest that the Andes constitutes a semipermeable barrier to biotic dispersal, and that orchids may be more geographically constrained by intrinsic factors, such as fungal symbionts and pollinator mutualists, which differ among elevational zones (Arroyo *et al*., 1982, 1985; Lugo *et al*., 2008) than by distance. The dependence of immigrant orchids on particular fungal or pollinator mutualists, matched to the available pool of mutualists, may greatly determine the success of their establishment in a new area.

Our findings of widespread within‐region speciation as the main biogeographical process (Figs 1, 2, S16, S17), coupled with the apparent widespread permeability of the Andean mountains to lowland migrants, raise the question of the speciation mechanisms underlying these fast speciation rates. We speculate that the habitat heterogeneity, with many adjacent but distinct niches, could have favoured isolation, perhaps via peripatric or parapatric speciation. Be as it may, our work paves the way for microevolutionary studies of orchid speciation in the Andes.

### Accelerated orchid diversification across elevational zones

Gentry's hypothesis (Gentry, 1982) of rapid speciation in the Andes was mainly based on the observation of floristic groups (e.g. ‘Andean‐centred taxa’) with very speciose genera from the lowlands to mid‐elevations in the (mostly Northern) Andes. This matches well the total altitudinal distribution of our respective study groups, with a richness vs elevation plot for > 55% of the 3700 Cymbidieae species based on over 20 000 records (Figs 3a, S1), which reveals that Cymbidieae diversity peaks at low elevations (< 1100 m), whereas Pleurothallidinae diversity (*c*. 10 000 records; Fig. S2) peaks at *c*. 1500 m (Fig. 4a).

The diversification rate shifts are all located within clades that already inhabited the Northern Andes, and temporally match with periods of accelerated Andean uplift in this region (Gregory‐Wodzicki, 2000; Hoorn *et al*., 2010) (Figs 1, 2). The late middle Miocene and early Pliocene are the periods with the fastest documented rates of Andean uplift in the Northern Andes (i.e. Venezuelan Andes and Northern Andes of Colombia; Hoorn *et al*., 1995; Bermúdez *et al*., 2015). In all three Cymbidieae clades, speciation rates peaked at 6 Ma, a time at which the Northern Andes reached *c*. 4000 m, their maximum mean palaeo‐elevation (Bermúdez *et al*., 2015). Contrary to Cymbidieae, we found no correlation between Andean uplift and Pleurothallidinae diversification (Table S11). We hypothesize that this is a result of the rapid diversification of migrating cool pre‐adapted Pleurothallidinae lineages from Central America into already formed montane environments (Hoorn *et al*., 2010). Similar diversification patterns have been reported for *Lupinus*,* Bartsia*, Adoxaceae, Valerianaceae and, more recently, Ericaceae (Donoghue & Sanderson, 2015; Schwery *et al*., 2015; Uribe‐Convers & Tank, 2015).

Gentry proposed that the main mechanism underlying rapid speciation in the Andes was the evolution of novel plant–insect interactions (Gentry, 1982). The Cymbidieae are particularly known among biologists and ecologists because of the rich array of pollination syndromes and sexual systems they have evolved (e.g. sexual and food deceit, food and fragrance reward, dichogamy and environmental sex determination; Gerlach & Schill, 1991; Singer, 2002; Pansarin *et al*., 2009; Gerlach & Pérez‐Escobar, 2014). Our analyses suggest that pollinator syndrome shifts do not match with diversification rate shifts, although our data do not take into account pollinator shifts within given pollinator groups. This is particularly true for the bee pollination syndrome, which is widespread in the tribe and probably overarches several transitions from different types of bees (e.g. oil to euglossine bees as observed in Catasetinae). More field observations of pollinations are therefore needed to evaluate the relative role of pollinator shifts in contributing to Neotropical orchid diversification.

### Conclusion

Based on two extensively sampled orchid phylogenies, combined with statistically robust diversification models, our results reveal that Andean orchid diversification has closely tracked the Andean orogeny. Together with studies in other mega‐diverse regions (Verboom *et al*., 2009; Bruyn *et al*., 2014), our results show that rapid recent speciation has moulded this area of exceptional species richness. In addition, our results highlight the crucial role of Amazonian lowlands, as well as the Antillean and Central American regions, as biotic sources for Andean biodiversity, providing cool pre‐adapted lineages that dispersed into the Andes and further diversified *in situ*.

Contrary to general expectation, the rise of the Andes had little effect on restricting orchid biotic dispersal across the Neotropics. This suggests that mountains are semi‐permeable barriers to lowland organisms, whose dispersal ability is more probably related to intrinsic traits (e.g. seed size, dispersal mechanism, mutualisms). Although both abiotic and biotic processes are clearly responsible for the exceptional species richness of the world's premier biodiversity hotspot (Antonelli & Sanmartín, 2011; Hughes *et al*., 2013; Eiserhardt *et al*., 2017), our results suggest that geological processes played a central and direct role in the diversification process. Finally, as the highest species richness in Cymbidieae is concentrated in the lowlands and the Pleurothallidinae peak is at mid‐elevation, our study shows that Andean uplift dramatically affected the evolutionary assembly of both lowland and mid‐elevation Andean forests, as originally hypothesized by Gentry (1982).

## Author contributions

O.A.P‐E., G.C. and A.A. designed the research; O.A.P‐E., A.P.K., D.B. and G.C. performed the research; O.A.P‐E., G.C., F.L.C. and N.J.M. analysed the data; F.L.C. and D.S. contributed analytical tools; G.C. and O.A.P‐E. wrote the paper with contributions from all authors.

## Supporting information

Please note: Wiley Blackwell are not responsible for the content or functionality of any Supporting Information supplied by the authors. Any queries (other than missing material) should be directed to the *New Phytologist* Central Office.

**Fig. S1** Distribution map of Cymbidieae obtained from collection records.
**Fig. S2** Distribution map of Pleurothallidinae obtained from collection records.
**Fig. S3** Potential conflicting terminals between nuclear‐ and plastid‐derived phylogenies of Cymbidieae as inferred by PACo (Procrustes approach to cophylogeny).
**Fig. S4** Potential conflicting terminals between nuclear‐ and plastid‐derived phylogenies of Pleurothallidinae as inferred by PACo (Procrustes Approach to Cophylogeny).
**Fig. S5** Maximum likelihood phylogeny of Cymbidieae inferred from concatenated nuclear and plastid loci.
**Fig. S6** Maximum likelihood phylogeny of Pleurothallidinae inferred from concatenated nuclear and plastid loci.
**Fig. S7** Chronogram for Orchidaceae obtained under a relaxed clock model.
**Fig. S8** Chronogram for Cymbidieae obtained under a relaxed clock model.
**Fig. S9** Chronogram for Pleurothallidinae obtained under a relaxed clock model.
**Fig. S10** Biogeographical history of Cymbidieae.
**Fig. S11** Ancestral character estimation of altitude on a chronogram of Cymbidieae.
**Fig. S12** Traitgram of altitude change as a function of time in Cymbidieae.
**Fig. S13** Biogeographical history of Pleurothallidinae.
**Fig. S14** Ancestral character estimation of altitude on a chronogram of Pleurothallidinae.
**Fig. S15** Traitgram of altitude change as a function of time in Pleurothallidinae.
**Fig. S16** Frequency of relevant biogeographical events through time in Cymbidieae.
**Fig. S17** Frequency of relevant biogeographical events through time in Pleurothallidinae.
**Fig. S18** Phylorate plot of Cymbidieae and the three best configuration shifts in diversification rates.
**Fig. S19** Tip speciation rates of Cymbidieae clades with diversification rate shifts compared with other lowland‐based clades.
**Fig. S20** Phylorate plot of Pleurothallidinae and the three best configuration shifts in diversification rates.
**Fig. S21** Tip speciation rates of Pleurothallidinae clades with diversification rate shifts compared with other lowland‐based clades.
**Fig. S22** Diversity through time of Cymbidieae clades with diversification rate shifts compared with other lowland‐based clades.
**Fig. S23** Diversity through time of Pleurothallidinae clades with diversification rate shifts compared with other lowland‐based clades.
**Fig. S24** Null distribution of ∆AIC obtained from simulated geographical distribution data of Cymbidieae and Pleurothallidinae.
**Fig. S25** Distribution of Cymbidieae species nested in clades with shifts on diversification rates and lineages with low speciation rates.
**Fig. S26** Distribution of Pleurothallidinae species nested in clades with shifts on diversification rates and lineages with low speciation rates.
**Fig. S27** Density herbarium record of Cymbidieae.
**Fig. S28** Density herbarium record of Pleurothallidinae.
**Fig. S29** Ancestral character estimation of pollination syndromes of Cymbidieae.
**Table S1** Species name and voucher information for material used to build the Cymbidieae dataset
**Table S2** Species name and voucher information for material used to build the Pleurothallidinae dataset
**Table S3** Comparison of different biogeographical models fitted on the Cymbidieae dataset
**Table S4** Comparison of different biogeographical models fitted on the Pleurothallidinae dataset
**Table S5** Bayes factor of different diversification rate models fitted on the Cymbidieae dataset
**Table S6** Bayes factor of different diversification rate models fitted on the Pleurothallidinae dataset
**Table S7** Akaike information criterion (AIC) values for diversification models fitted on Andean‐centred Cymbidieae clades and lowland‐centred lineages
**Table S8** Akaike information criterion (AIC) values for diversification models fitted on Andean‐centred Pleurothallidinae clades and lineages without diversification rate shifts
**Table S9** Akaike information criterion (AIC) values for area diversification models fitted on the Cymbidieae and Pleurothallidinae datasets
**Table S10** Results of palaeo‐elevation‐dependent model of diversification in Northern Andean Cymbidieae clades
**Table S11** Results of palaeo‐elevation‐dependent model of diversification in Northern Andean Pleurothallidinae clades
**Methods S1** Extended materials and methods.
