# Supplementary material for: Recent origin and rapid speciation of Neotropical orchids in the world's richest plant biodiversity hotspot
Source: New Phytol. 2017 Jun 20;215(2):891–905. doi: 10.1111/nph.14629 (PMC5575461; doi:10.1111/nph.14629)
Supplement: Supplementary file 1 — Fig. S1 Distribution map of Cymbidieae obtained from collection records. Fig. S2 Distribution map of Pleurothallidinae obtained from collection records. Fig. S3 Potential conflicting terminals between nuclear‐ and plastid‐derived phylogenies of Cymbidieae as inferred by PACo (Procrustes approach to cophylogeny). Fig. S4 Potential conflicting terminals between nuclear‐ and plastid‐derived phylogenies of Pleurothallidinae as inferred by PACo (Procrustes Approach to Cophylogeny). Fig. S5 Maximum likelihood phylogeny of Cymbidieae inferred from concatenated nuclear and plastid loci. Fig. S6 Maximum likelihood phylogeny of Pleurothallidinae inferred from concatenated nuclear and plastid loci. Fig. S7 Chronogram for Orchidaceae obtained under a relaxed clock model. Fig. S8 Chronogram for Cymbidieae obtained under a relaxed clock model. Fig. S9 Chronogram for Pleurothallidinae obtained under a relaxed clock model. Fig. S10 Biogeographical history of Cymbidieae. Fig. S11 Ancestral character estimation of altitude on a chronogram of Cymbidieae. Fig. S12 Traitgram of altitude change as a function of time in Cymbidieae. Fig. S13 Biogeographical history of Pleurothallidinae. Fig. S14 Ancestral character estimation of altitude on a chronogram of Pleurothallidinae. Fig. S15 Traitgram of altitude change as a function of time in Pleurothallidinae. Fig. S16 Frequency of relevant biogeographical events through time in Cymbidieae. Fig. S17 Frequency of relevant biogeographical events through time in Pleurothallidinae. Fig. S18 Phylorate plot of Cymbidieae and the three best configuration shifts in diversification rates. Fig. S19 Tip speciation rates of Cymbidieae clades with diversification rate shifts compared with other lowland‐based clades. Fig. S20 Phylorate plot of Pleurothallidinae and the three best configuration shifts in diversification rates. Fig. S21 Tip speciation rates of Pleurothallidinae clades with diversification rate shifts compared with other lowland‐based clades. F [file NPH-215-891-s001.pdf]

New Phytologist

***New Phytologist* Supporting Information**

Article title: Recent origin and rapid speciation of Neotropical orchids in the world's richest plant biodiversity hotspot

Authors: Oscar Alejandro Pérez-Escobar[a,1], Guillaume Chomicki[b,1], Fabien L. Condamine[c], Adam P. Karremans[d,e], Diego Bogarín[d,e], Nicholas J. Matzke[f], Daniele Silvestro[g,h], Alexandre Antonelli[g,i]

[a]Identification and Naming Department, Royal Botanic Gardens, Kew, Richmond, Surrey, TW9 3DS, UK. [b]Systematic Botany and Mycology, University of Munich (LMU), 67 Menzinger Str., Munich 80638, Germany. [c]CNRS, UMR 5554 Institut des Sciences de l'Evolution (Université de Montpellier), Place Eugène Bataillon, 34095 Montpellier, France. [d]Lankester Botanical Garden, University of Costa Rica, P.O. Box 302-7050 Cartago, Costa Rica. [e]Naturalis Biodiversity Center, Leiden, 2333 CR, The Netherlands. [f]Division of Ecology, Evolution, and Genetics, Research School of Biology, The Australian National University, Canberra, ACT 2601, Australia. [g]Department of Biological and Environmental Sciences, University of Gothenburg, 413 19 Gothenburg, Sweden; [h]Department of Ecology and Evolution, Biophore, University of Lausanne, 1015 Lausanne, Switzerland; [i]Gothenburg Botanical Garden, Carl Skottsbergs gata 22A, 41319, Gothenburg, Sweden.

[1]These authors contributed equally to this study.

Article acceptance date: 07 April 2017

The following Supporting Information is available for this article:

Methods S1

Supplementary figures S1-S29

Supplementary tables S1-S11

Methods S1 Extended Materials and Methods

Molecular clock dating of Orchidaceae-wide phylogeny

Molecular clock dating relied on the uncorrelated lognormal relaxed clock model implemented in BEAST v.2 (Bouckaert *et al.*, 2014). We also used the GTR + G nucleotide substitution model with 6 rate categories, a Yule tree prior, and a Markov chain Monte Carlo (MCMC) chain length of 100 million generations, sampling every 10,000 generations. Four fossil calibrations were used. The oldest known fossil of the Asparagales (105 million yr; Walker & Walker, 1984) was used as a maximal calibration point for the Orchidaceae, following Gustafsson *et al.*, (2010), and was given a large normal prior (offset = 105.3; stdev = 8). A gamma distribution was used on each of the three ingroup fossil constraints (the offset value was set to the minimum age of each fossil and the standard deviation was set such that the maximum age was included in the 97.5% quantile). The fossil orchid *Meliorchis caribea* (15–20 million yr) has been assigned to the Goodyerinae (Ramírez *et al.*, 2007), and provided a minimum age for the subtribe (offset = 15; stdev= 5). Two recently discovered orchid macrofossils (*Dendrobium winikaphyllum* and *Earina fouldenensis*; both 20–23 million yr; Conran et al., 2009) were used as minimum constraints within Epidendroideae, with gamma priors (offset = 20; stdev= 4.5). The trees were summarized using TREEANNOTATOR v. 1.8.0 (part of the BEAST package), with a 10% burn-in and a 0.98 posterior probability limit. For the Cymbidieae and the Pleurothallidinae, we used the concatenated nuclear-plastid datasets, using parameters as described above, except the MCMC chain length, which consisted of two runs 100 million generations combined with LogCombiner (part of the BEAST software).

Ancestral range estimation

To ensure that all taxa sampled in our Cymbideae and Pleurothallidinae phylogenies were represented by at least one distribution record, we further included manually in our geographical record databases the type localities of species without available herbariums specimens. Distribution maps of Cymbideae and Pleurothallidinae were generated on the software ARCGIS (Esri). To better understand diversity distribution patterns in Pleurothallidiane and Cymbidiae, we further generated density herbarium records for both clades based on the

distribution data compiled from herbarium and online repositories (see above). Data stored at online repositories such as GBIF is often redundant, and herbarium records are subject to sampling intensity (i.e. poorly sampled areas *vs*. extensively sampled areas) (Hijmans & Elith, 2016). To account for these biases, we filtered distribution record data by randomly sampling one distribution record for each 0.50 × 0.50 arc degree cell following Hijmans & Elith' (2016) protocol. Density herbarium records maps (Figs. S27, S28) were generated by extrapolating the number of occurrences per grid cell (1 × 1 arc degree) to the geographical distribution range of Cymbidieae and Pleurothallidinae.

We generated 50 biogeographical stochastic maps in BioGeoBEARS, and plotted the relative frequencies of all four biogeographic processes over time for all geographic areas together and separately. Ancestral range estimations and stochastic character mapping were performed on the chronogram obtained in the dating analysis. BioGeoBEARS requires fully bifurcating trees (i.e. trees without negative branches or polytomies) as input data. Therefore, we pruned from our Cymbidieae dated phylogeny the branch leading to all Coleopsidinae species (4 tips) because it formed a polytomy together with the clade including all Maxillariinae sampled taxa. We ran our BioGeoBEARS analyses in a high performance Linux cluster at the Leibniz Supercomputing Centre (LRZ, Munich, Germany).

Ancestral character state estimation

We carried out Maximum Likelihood estimations of discrete (i.e. pollination syndromes) and continuous (i.e. altitude) characters on the maximum clade credibility trees obtained from BEAST analysis. We relied on the functions *make.simmap* and *contMap* of the R package PHYTOOLS (Revell, 2012) for the estimation of discrete and continuous characters, respectively. To account for phylogenetic uncertainty, we executed every ACS estimation on 1000 randomly sampled, post burn-in trees derived from the BEAST analyses. For pollination syndromes' ACS estimation, a single rate model (ER) fit best our data over Symmetric rate (SYM) and All Rate Dependent (ARD) models, and probability scores of every state at nodes were summarized across the 1000 phylogenies as posterior probability values. A comparison of likelihood values from all implemented models revealed that SYM is the fittest model for pollination syndrome

ACS. In addition, to better understand how altitudinal range has varied over time in Pleurothallidinae and Cymbidieae, we estimated the variation of mean altitude values as a continuous character over Ma, using the function phenogram of the R package PHYTOOLS (Revell, 2012).

Paleo-elevation-dependent diversification

We developed and applied four models in RPANDA in which rates were modified by an exponential function of paleo-environment: (*i*) speciation rate varies with the environmental variable and no extinction; (*ii*) speciation rate varies with the environmental variable and extinction rate is constant; (*iii*) speciation rate is constant and extinction rate varies with the environmental variable; and (*iv*) speciation and extinction rates both vary with the environmental variable. These models were compared to simpler diversification models, namely a pure speciation model (Yule) and a constant rate birth-death models. Speciation and extinction rates inferred under the best model were plotted through time. The strength of the correlation between diversification rates and the paleo-environmental variables is indicated by the α (speciation) and β (extinction) parameters; these parameters additionally determine the degree to which the resulting diversification curve approximates the input paleo-environmental data.

Rates of species diversification in RPANDA

We further cross-validated rates of species diversification rates obtained in BAMM by estimating rate variation through time in the four clades for which BAMM detected shifts of diversification rates (see *results*) using the R package RPANDA v.1.1 (Morlon *et al.*, 2016). We compared rate variation of these clades by including in the analysis predominantly lowland based lineages (Catasetinae in Cymbidieae, and *Specklinia* in Pleurothallidinae), in which no shifts of diversification rates were detected. We fit four models following Morlon et al. (2011), with different settings of constant and variable speciation and extinction rates as follows: (*i*) M_1: constant speciation and extinction rates; (*ii*) M_2: constant extinction rates but variable speciation rates; (*iii*) M_3: constant speciation rates but variable extinction rates; (*iv*) M_4: variable speciation and extinction rates.

Geographic state-dependent analyses

We tested three different models: (*i*) M_a: free speciation and extinction rates in A, B and between regions (i.e. AB); (*ii*) M_b: within-region speciation not allowed (i.e. set to 0) and (*iii*) M_c: independent regional speciation and extinction are equal in A and B set to be equal (sA =sB; xA=xB). We chose an epsilon value (speciation/extinction ratio) of 0.114, which is specific for the group of study (estimated for higher monocots by Magallón & Sanderson, [2001]), to generate a starting point for parameter estimation, as suggested in the manual. We summarized the posterior distributions of samples to assess variation in net diversification, turnover, and transition rates across areas. We further simulated neutral characters by randomly reshuffling the coded areas onto the phylogeny to determine whether the diversification rates associated with biogeographic areas detected in GeoSSE could be artefacts because of Type I errors biases in SSE models (Rabosky & Goldberg, 2015). We carried 1000 iterations, estimating the AICc of the best model for each iteration to get the AICc null distribution and compare it with our real dataset AIC model value (i.e. ΔAIC).

New Phytologist

References

Bouckaert R, Heled J, Kühnert D, Vaughan T, Wu CH, Xie D, Suchard MA, Rambaut A, Drummond AJ. **2014**. BEAST 2: a software platform for Bayesian evolutionary analysis. *PLoS Computational Biology* **10**: 1–6.

Gustafsson ALS, Verola CF, Antonelli A. **2010**. Reassessing the temporal evolution of orchids with new fossils and a Bayesian relaxed clock, with implications for the diversification of the rare South American genus *Hoffmannseggella* (Orchidaceae : Epidendroideae). *BMC evolutionary biology* **10**: 177–190.

Hijmans RJ, Elith J. **2016**. Species distribution modeling with R. *R package version 0.8. https://cran.r-project.org/package=raster.*

Magallón S, Sanderson MJ. **2001**. Absolute diversification rates in angiosperm clades. *Evolution* **55**: 1762–1780.

Morlon H, Lewitus E, Condamine FL, Manceau M, Clavel J, Drury J. **2016**. RPANDA: An R package for macroevolutionary analyses on phylogenetic trees. *Methods in Ecology and Evolution* **7**: 589–597.

Morlon H, Parsons TL, Plotkin JB. **2011**. Reconciling molecular phylogenies with the fossil record. *Proceedings of the National Academy of Sciences of the United States of America* **108**: 16327-16332.

Rabosky DL, Goldberg EE. **2015**. Model inadequacy and mistaken inferences of trait-dependent speciation. *Systematic Biology* **64**: 340–355.

Ramírez SR, Gravendeel B, Singer RB, Marshall CR, Pierce NE. **2007**. Dating the origin of the Orchidaceae from a fossil orchid with its pollinator. *Nature* **448**: 1042–1045.

Revell LJ. **2012**. Phytools: an R package for phylogenetic comparative biology (and other things). *Methods in Ecology and Evolution* **3**: 217–223.

Walker JW, Walker AG. **1984**. Ultrastructure of lower Cretaceous angiosperm pollen and early evolution of flowering plants. *Annals of the Missouri Botanical Garden* **71**: 464–521.

Supplementary figures

Fig. S1 Distribution map of Cymbidieae obtained from ca 19000 records sourced from herbaria collections and GBIF (see methods).

Fig. S2 Distribution map of Pleurothallidinae obtained from ca 10000 records sourced from herbaria collections and GBIF (see methods).

Fig. S3 Potential conflicting Operational Terminal Units (OTUs) between nuclear and plastid derived phylogenies of Cymbidieae as inferred by PACo. Terminals with normalized squared residual values higher than the cut-off threshold (red line) are potentially conflicting taxa.

Fig. S4 Potential conflicting Operational Terminal Units (OTUs) between nuclear and plastid derived phylogenies of Pleurothallidinae as inferred by PACo. Terminals with normalized squared residual values higher than the cut-off threshold (red line) are potentially conflicting taxa. Blue bars represent 95% empirical confidence intervals are shown.

Fig. S5 Maximum Likelihood phylogeny of the orchid tribe Cymbidieae inferred from concatenated nuclear and plastid loci. Likelihood Bootstrap Support (LBS) values > 75 are shown at nodes of the tree (black pie diagrams indicate LBS of 100). Sub-tribe members of Cymbidieae are colour-coded. Pictures enclosed on circles show selected representatives of (a) Cymbidiinae (*Grammatophyllum measuresianum*); (b) Catasetinae (*Cycnoches egertonianum*); (c) Eulophiinae (*Eulophia streptopetala*); (d) Zygopetaliinae (*Zygopetalum* aff. *brachypetalum*); (e) Coeliopsidinae (*Peristeria cerina*); (f) Stanhopeinae (*Stanhopea maculosa*); (g) Maxillariinae (*Sudamerlycaste cinnabarina*); (h) Oncidiinae (*Trichoceros* sp.). Photos: O. Pérez.

Fig. S6 Maximum Likelihood phylogeny of the orchid subtribe Pleurothallidinae inferred from concatenated nuclear and plastid loci. Likelihood Bootstrap Support (LBS) values > 75 are shown at nodes of the tree (black pie diagrams indicate LBS of 100). Generic members of Pleurothallidinae are colour-coded. Pictures enclosed on circles show selected representatives of (a) *Lepanthes* (*Lepanthes* sp.); (b) *Dracula* (*D. astuta*); (c) *Masdevallia* (*M. utricularia*); (d) *Muscarella* (*M. exesilabia*); (e) *Platystele* (*P. porquinqua*); (f) *Pabstiella* (*P. ephemera*); (g) *Pleurothallis* (*P. adventurae*); (h) *Dresslerella* (*D. pilosissima*); (i) *Myoxanthus* (*M. colothrix*); (j) *Acianthera* (*A. cogniauxiana*). Photos: A. Karremans, D. Bogarín and O. Pérez.

Fig. S7 Chronogram for Orchidaceae obtained under a relaxed clock model, applied to a concatenated nuclear ITS, and plastid *mat*K, *trn*L-F, and *rbc*L loci. The Most Recent Common Ancestors (MRCAs) of Orchidaceae, Cymbidiee and Pleurothallidinae are highlighted with stars. Mean absolute ages and 95% Confidence Intervals (blue bars) are shown. Time scale is provided in million years ago (MYA).

Fig. S8 Chronogram of Cymbidieae obtained under a relaxed clock model, applied to concatenated nuclear and plastid loci. 95% Confidence Intervals (blue bars) are shown. Time scale is provided in million years ago (MYA). Sub-tribal members of Cymbidieae are colour-coded.

Fig. S9 Chronogram of Pleurothallidinae obtained under a relaxed clock model, applied to concatenated nuclear and plastid loci. 95% Confidence Intervals (blue bars) are shown. Time scale is provided in million years ago (MYA). Generic members of Pleurothallidinae are colour-coded.

Fig. S10 Biogeographic history of Cymbidieae orchids. Pie diagrams at nodes indicate the probability of estimated ancestral area as inferred by BioGeoBEARS. Grey pies indicate nodes with ambiguous ancestral areas. Geographical ranges of every taxon are shown as vertical bars in front of the terminals. Time scale is provided in million years ago (MYA). The Most Recent Common Ancestor (MRCA) of Cymbidieae is indicated with a star. (Inset) Coded areas for biogeographical analysis. Political division obtained from DIVA-GIS (http://www.diva-gis.org/gdata).

Fig. S11 Ancestral character estimation of altitude as a continuous character on a chronogram of Cymbidieae. Changes in diversification rates are indicated with red circles. Sub-tribal members of Cymbidieae are colour-coded. Ancestral areas of ancestors estimated to have inhabited in Amazonia and Northern Andes are shown. Time scale is provided in million years ago (MYA). Note that almost all shifts on diversification rates took place at Andean based clades.

Fig. S12 Ancestral character estimation of altitude as a function of time (MYA). The graph shows variation of mean elevation values across time in Cymbidieae. Note the elevation value of ~900

m reconstructed at the Cymbidieae Most Recent Common Ancestor (MRCA), and mean elevation values of the Andean ancestors (colour-coded) as inferred by BioGeoBEARS centred between ~900 – 1300 m.

Fig. S13 Biogeographic history of Pleurothallidinae orchids. Pie diagrams at nodes indicate the probability of estimated ancestral area as inferred by BioGeoBEARS. Grey pies indicate nodes with ambiguous ancestral areas. Geographical ranges of every taxon are shown as vertical bars in front of the terminals. Time scale is provided in million years ago (MYA). The Most Recent Common Ancestor (MRCA) of Pleurothallidinae is indicated with a star. (Inset) Coded areas for biogeographical analysis. Political division obtained from DIVA-GIS (http://www.diva-gis.org/gdata).

Fig. S14 Ancestral character estimation of altitude as a continuous character on a chronogram of Pleurothallidinae. Changes in diversification rates are indicated with red circles. Generic members of Pleurothallidinae are colour-coded. Time scale is provided in million years ago (MYA). Ancestral areas of ancestors estimated to have inhabited in Central America and Northern Andes are shown. Note that all shifts on diversification rates took place at Andean based clades.

Fig. S15 Ancestral character estimation of altitude as a function of time (MYA). The graph shows variation of mean elevation values across time in Pleurothallidinae. Note the elevation value of ~1100 m reconstructed at the Pleurothallidinae Most Recent Common Ancestor (MRCA), and mean elevation values of Andean ancestors (colour-coded) as inferred by BioGeoBEARS centred between ~1200 – 1350 m.

Fig. S16 Frequency of relevant biogeographical events (i.e. founder speciation event, local extinction, within region speciation and vicariance) through time in A) Cymbidieae, and B) Northern Andean Cymbidieae clades as inferred by Biogeographical Stochastic Mapping (BSM). Time scale is provided in million years ago (MYA).

Fig. S17 Frequency of relevant biogeographical events (i.e. founder speciation event, local extinction, within region speciation and vicariance) through time in A) Pleurothallidiane, B) Northern Andean, and C) Central American Pleurothallidinae clades as inferred by Biogeographical Stochastic Mapping (BSM). Time scale is provided in million years ago (MYA).

Fig. S18 Phylorate plot of Cymbidieae and the tree best configuration shifts in diversification rates as inferred in BAMM (see methods). Colour intensity across branches is proportional to diversification rates. Posterior probabilities (*f*) of every configuration shift is provided. Black circles indicate the location of rate shifts, with circle size proportional to the marginal probability of the shift. Sub-tribal members of Cymbidieae are colour-coded.

Fig. S19 Tip speciation rates of Cymbidieae Andean clades with diversification rate shifts compared with other lowland based clades as inferred in BAMM (see methods). Grey bars represent minimum and maximum speciation rate values. Black circles represent outliers.

Fig. S20 Phylorate plot of Pleurothallidinae and the tree best configuration shifts in diversification rates as inferred in BAMM (see methods). Colour intensity across branches is proportional to diversification rates. Posterior probabilities (*f*) of every configuration shift is provided. Black circles indicate the location of rate shifts, with circle size proportional to the marginal probability of the shift. Generic members of Pleurothallidinae are colour-coded.

Fig. S21 Tip speciation rates of Pleurothallidinae Andean clades with diversification rate shifts compared with *Specklinia* clade (no diversification rate shifts) as inferred in BAMM. Grey bars represent minimum and maximum speciation rate values. Black circles represent outliers.

Fig. S22 Diversity through time of Cymbidieae Andean clades with diversification rate shifts compared with other lowland based clades as inferred in RPANDA (see methods).

Fig. S23 Diversity through time of Pleurothallidinae Andean clades with diversification rate shifts compared with *Specklinia* clade (no diversification rate shifts) as inferred in RPANDA.

Fig. S24 Null distribution of delta Akaike Informative Criterion (ΔAIC) obtained by randomly shuffling geographical distribution data of (A) Cymbidieae and (B) Pleurothallidinae in GeoSSE.

Fig. S25 Distribution of Cymbidieae species nested in clades with shifts on diversification rates (i.e. with high speciation rates) and lineages with low speciation rates. A) Maxillariinae; B) Oncidiinae; C) Zygopetalinae.

Fig. S26 Distribution of Pleurothallidinae species nested in clades with shifts on diversification rates (and hence high speciation rates) and lineages with low speciation rates (i.e. *Acianthera* species). A) *Lepanthes* + *Lepanthopsis*; B) *Masdevallia* + *Dracula*; C) *Pabstiella* + *Pleurothallis* + *Stelis*.

Fig. S27 Density herbarium record of Cymbidieae, based on ~1900 filtered distribution records. Colour intensity represent number of herbarium records per grid-cell (0.50 x 0.50 arc degrees; see methods).

Fig. S28 Density herbarium record of Pleurothallidinae, based on ~700 filtered distribution records. Colour intensity represent number of herbarium records per grid-cell (0.50 x 0.50 arc degrees; see *Methods*).

Fig. S29 Ancestral Character Estimation of pollination syndromes of Cymbidieae. Bars at nodes represent probabilities of states estimated at nodes. Coded character states for every taxon sampled in our phylogeny are shown as vertical bars in front of every taxon name. Shifts in diversification rates as inferred in BAMM are indicated with orange circles. Sub-tribe members of Cymbidieae are colour-coded.

Supplementary tables

Table S1 Species names and voucher information (GI - GenBank accession numbers) for material used to build Cymbidieae dataset. Taxa newly sequenced are indicated in bold letters. Names in bold correspond to newly sequenced taxa.

Table S2 Species names and voucher information (GenBank accession numbers) for material used to build Pleurothallidinae dataset. Taxa newly sequenced are indicated in bold letters. Names in bold correspond to newly sequenced taxa.

Table S3 Comparison of different biogeographic models fitted on Cymbidieae dataset as implemented in BioGeoBEARS package. Akaike Informative Criterion (AIC) values, including model weights and the corresponding ratios are provided for each model.

Table S4 Comparison of different biogeographic models fitted on Pleurothallidinae dataset as implemented in BioGeoBEARS package. Akaike Informative Criterion (AIC) values, including model weights and the corresponding ratios are provided for each model.

Table S5 Bayes Factor for a model with up to 10 diversification rate shifts compared to a null model with 0 shifts as inferred from Cymbidieae dataset.

Table S6 Bayes Factor of diversification rate shifts as inferred from Pleurothallidinae dataset.

Table S7 Akaike Informative Criterion (AIC) values for four diversification models fitted on

Andean-centred Cymbidieae clades with diversification shift rates compared with a lowland-centred lineage (Catasetinae) as inferred in RPANDA package. AIC values from the fittest model are highlighted in bold.

Table S8 Akaike Informative Criterion (AIC) values for four diversification models fitted on Andean-centred Pleurothallidinae clades with diversification shift rates compared with a clade without diversification shift rates (*Specklinia*) as inferred in RPANDA package. AIC values from the fittest model are highlighted in bold.

Table S9 AIC values for three area diversification models fitted on Cymbidieae and Pleurothallidinae dataset as inferred in GeoSSE. AIC values from the fittest model are highlighted in bold.

Table S10 Results of the paleo-elevation-dependent model of diversification on the four Northern Andean Cymbidieae clades (A-D). Models are as follows: two null models (a Yule model and a constant birth-death model); BAltiVar has only speciation that varies with past elevation; BAltiVarDcst has speciation that varies with past elevation and constant extinction; BcstDAltiVar has constant speciation and extinction; that varies with past elevation; and BAltiVarDAltiVar has both speciation and extinction that vary with past elevation. EXPO means exponential variation of the rates. Values are the means calculated from the fit of maximum likelihood models on a random sample of 100 dated trees (obtained from the BEAST dating analysis). The Akaike weight (ω) is used to compare all the models together to select the best-fit model (highlighted in bold).

Table S11 Results of the paleo-elevation-dependent model of diversification on three Northern Andean Pleurothallidinae clades (A-C). Models are as follows: two null models (a Yule model and a constant birth-death model); BAltiVar has only speciation that varies with past elevation; BAltiVarDcst has speciation that varies with past elevation and constant extinction; BcstDAltiVar has constant speciation and extinction; that varies with past elevation; and BAltiVarDAltiVar has both speciation and extinction that vary with past elevation. EXPO means exponential variation of the rates. Values are the means calculated from the fit of maximum likelihood models on a random sample of 100 dated trees (obtained from the BEAST dating analysis). The Akaike weight (ω) is used to compare all the models together to select the best-fit model (highlighted

in bold).

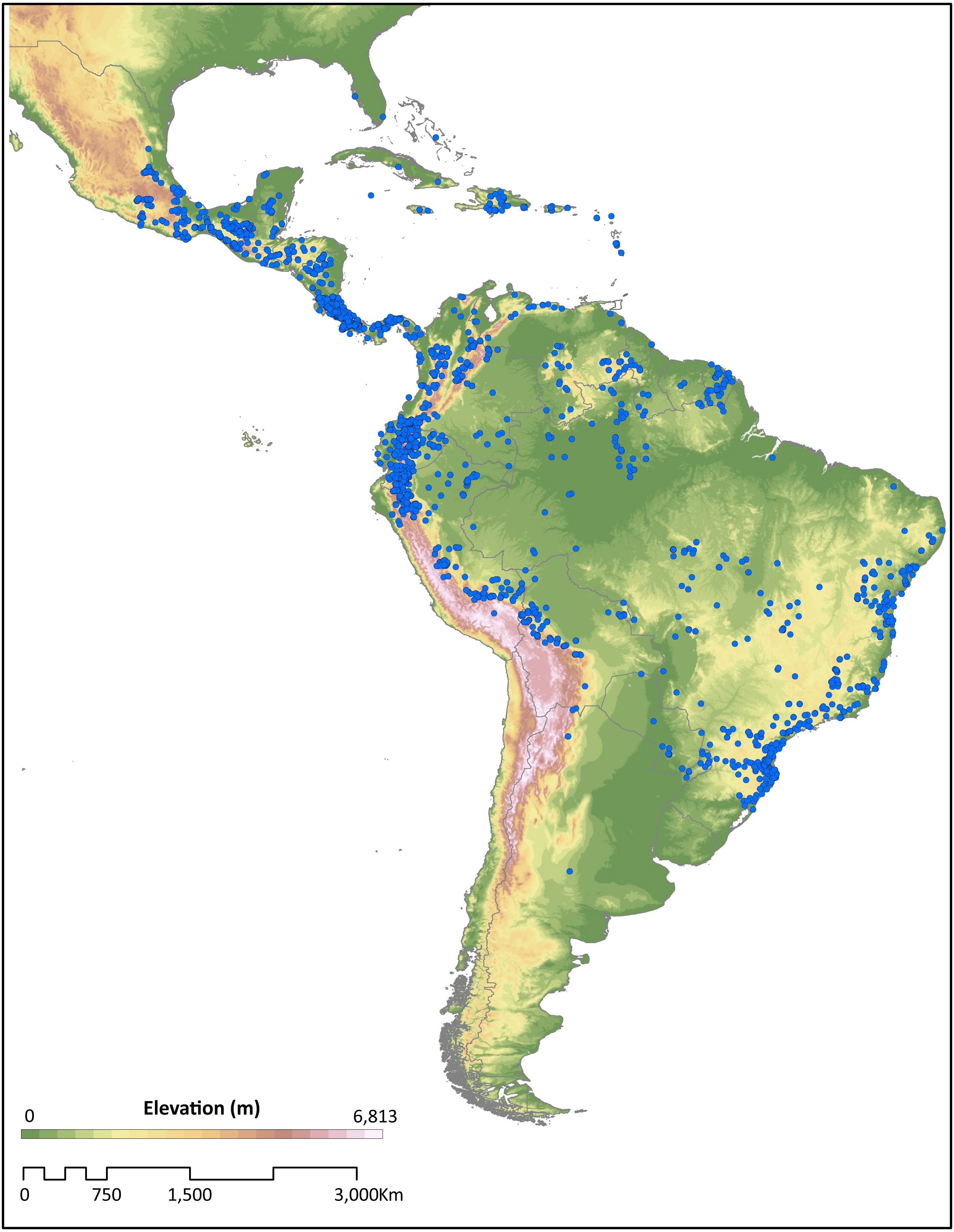

Figure S1. Distribution records of Cymbidieae (~19000 records)

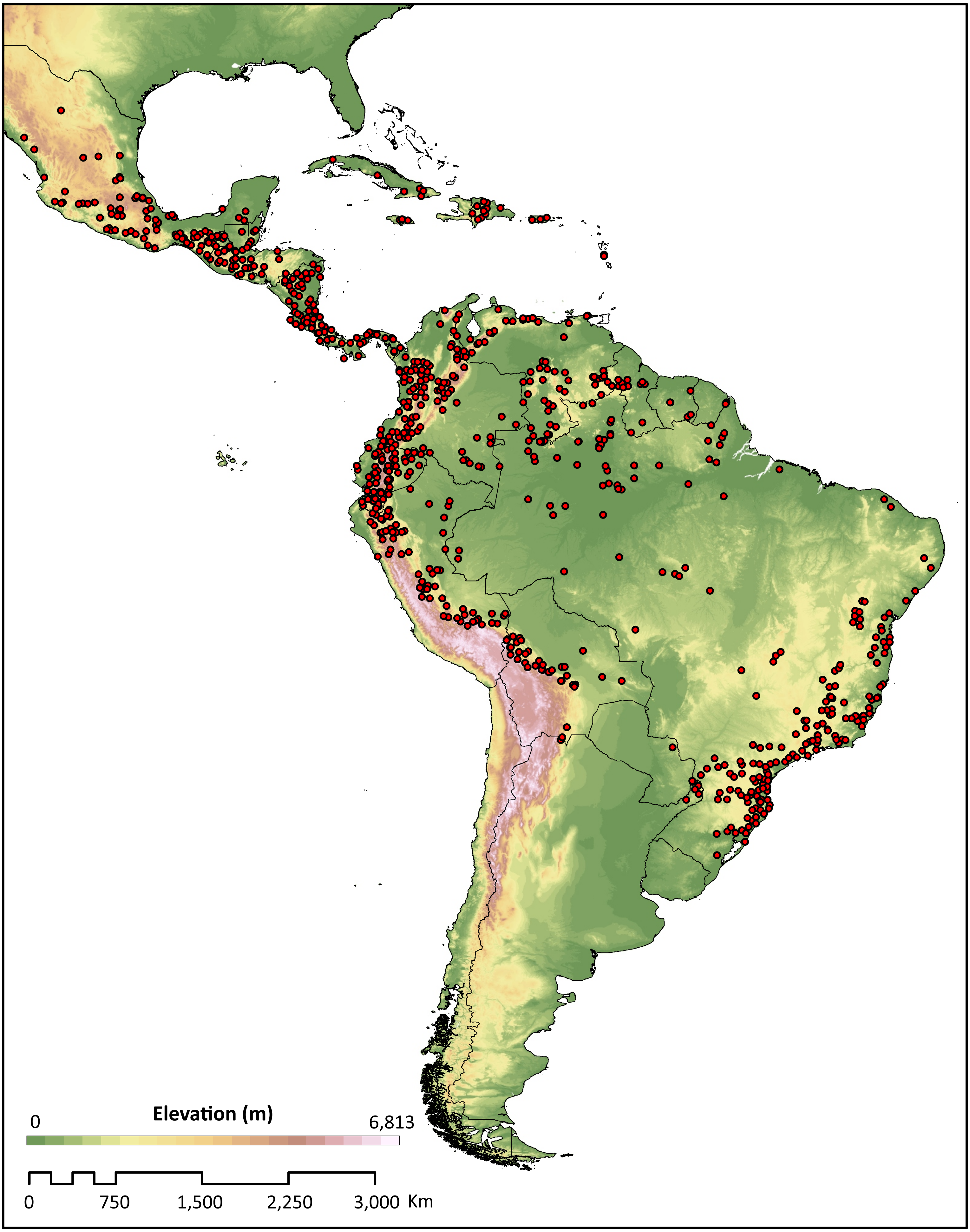

Figure S2. Distribution records of Pleurothallidinae (~10000 records)

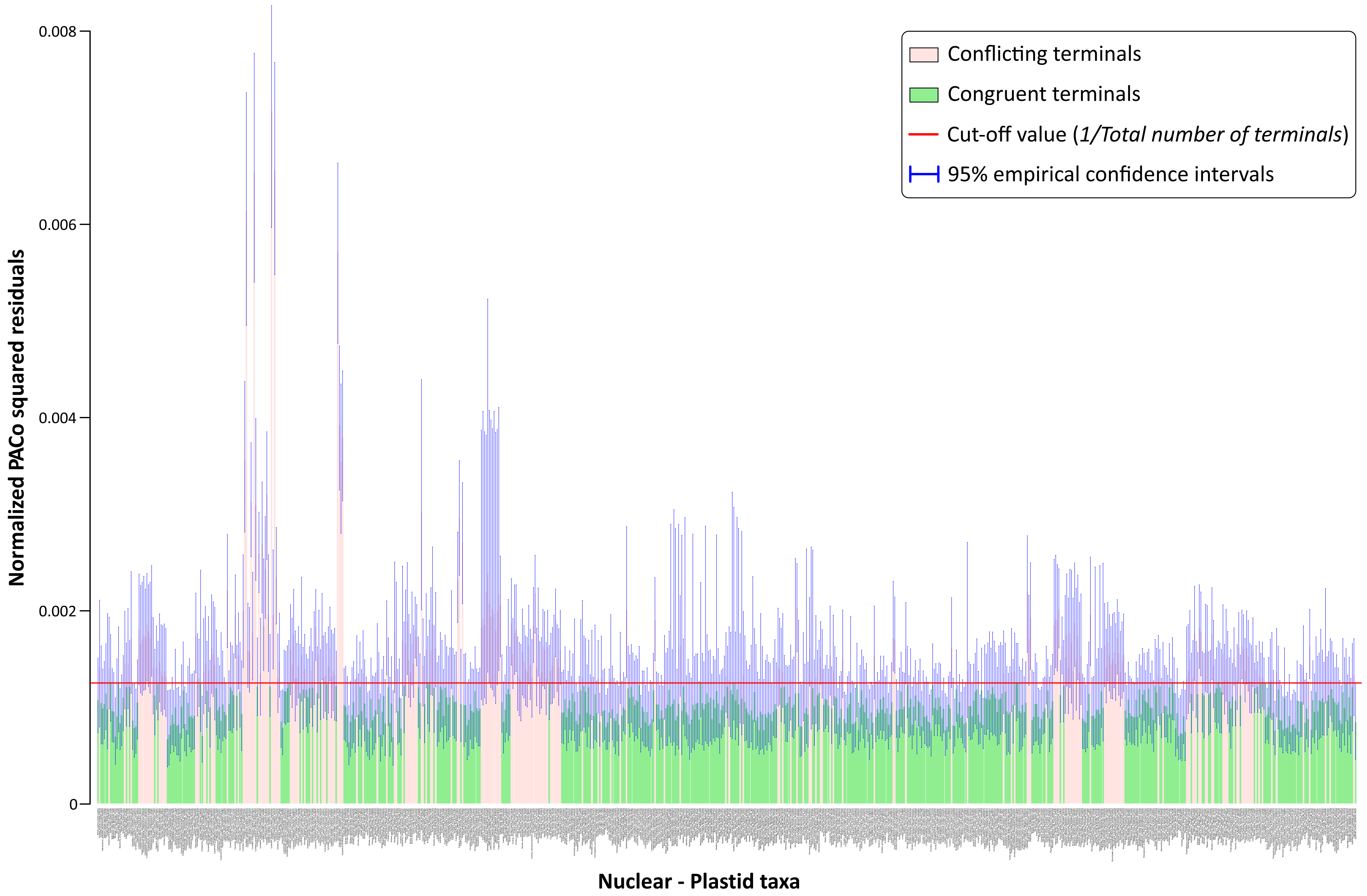

Figure S3. Potential conflicting OTUs between nuclear and plastid derived phylogenies in Cymbidieae

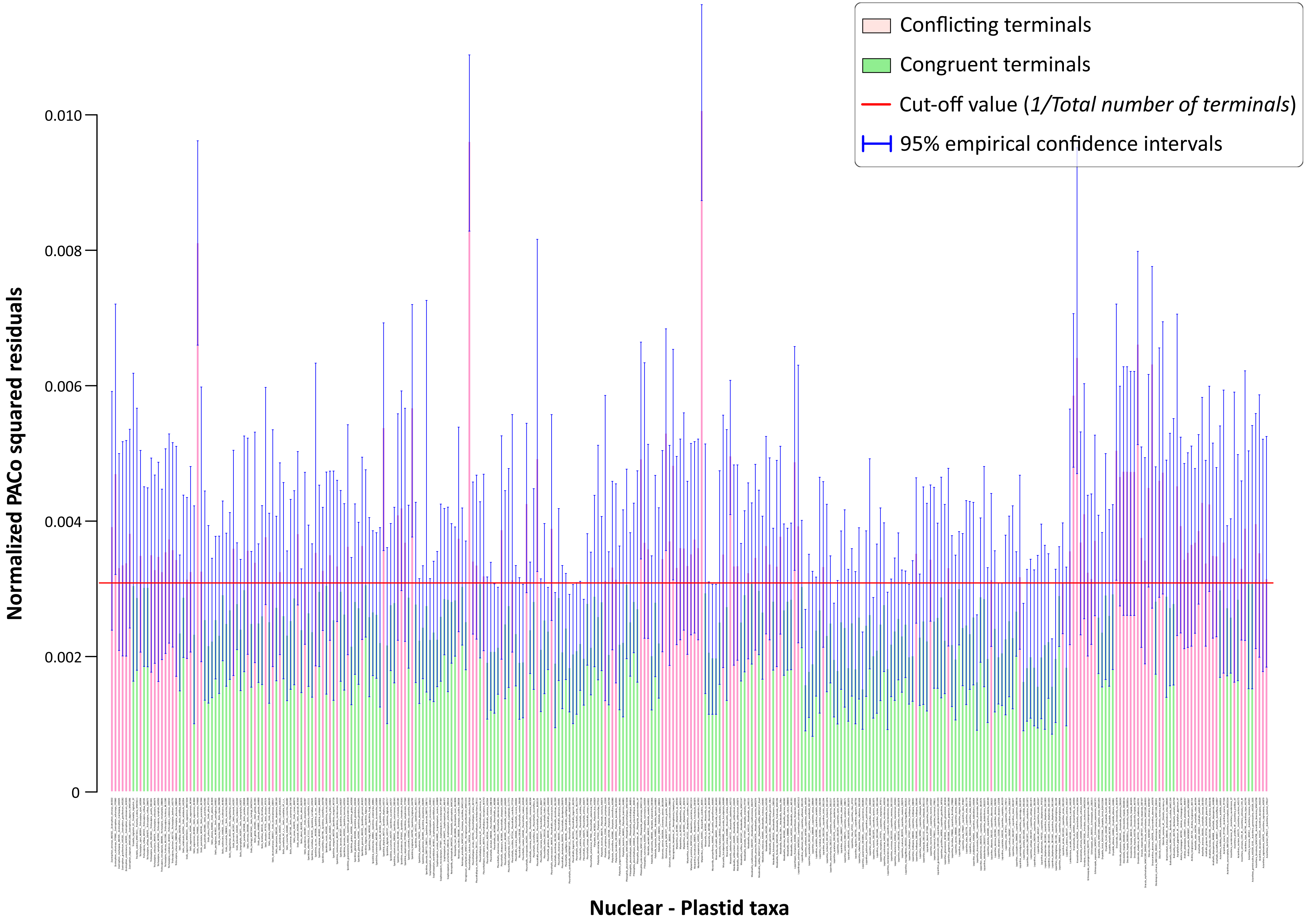

Figure S4. Potential conflicting OTUs between nuclear and plastid derived phylogenies in Pleurothallidinae

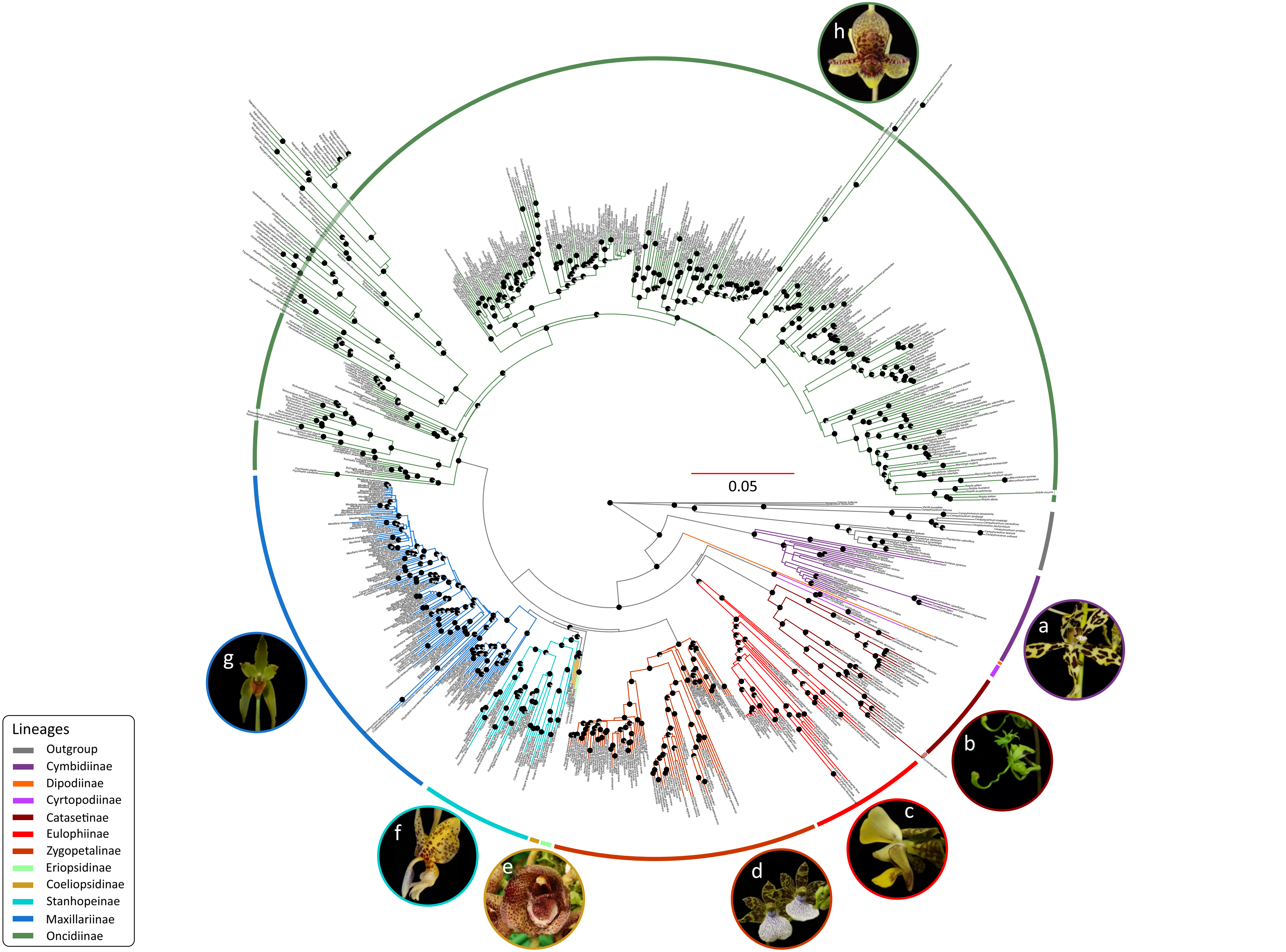

Figure S5. ML phylogeny of the orchid tribe Cymbidieae inferred from concatenated, non-conflicting nuclear and plastid loci. LBS >75 are shown at nodes of the tree (black pie diagrams indicate LBS of 100). Photos: O. Pérez

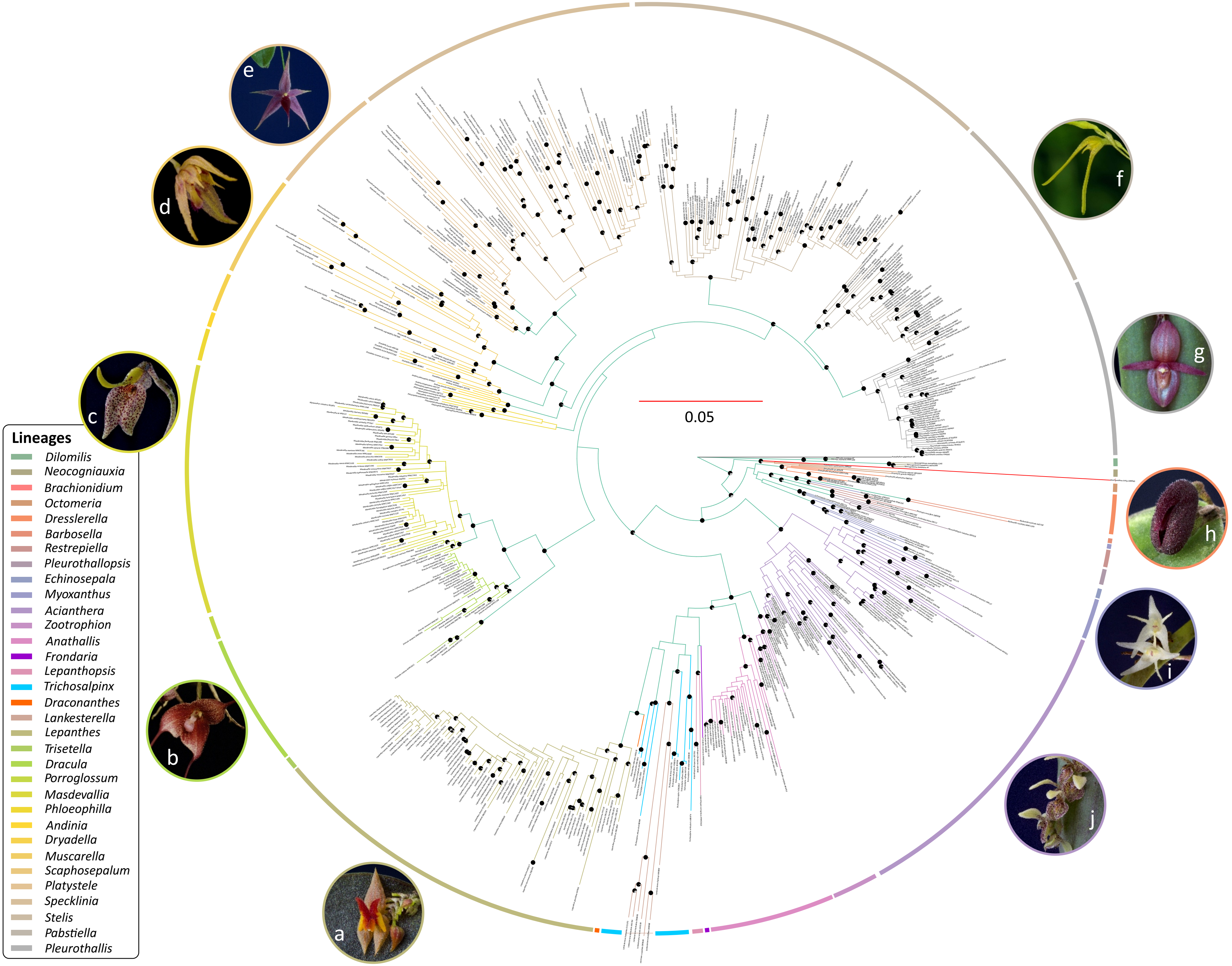

Figure S6. ML phylogeny of the orchid subtribe Pleurothallidinae inferred from concatenated, non-conflicting nuclear and plastid loci. LBS >75 are shown at nodes of the tree (black pie diagrams indicate LBS of 100). Photos: D. Bogarín, A. Karremans and O. Pérez

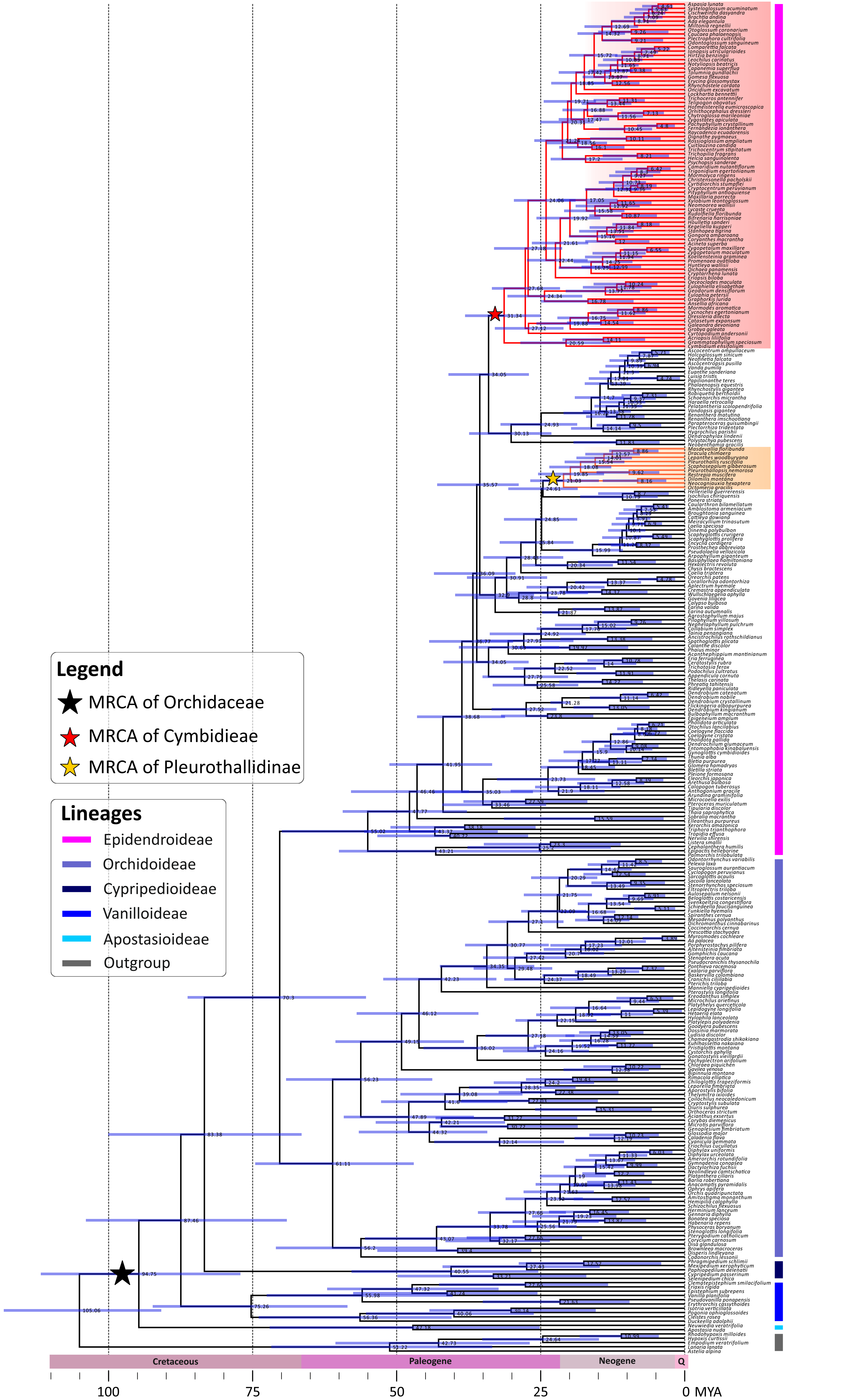

Figure S7. Chronogram of the Orchidaceae obtained a lognormal relaxed clock model

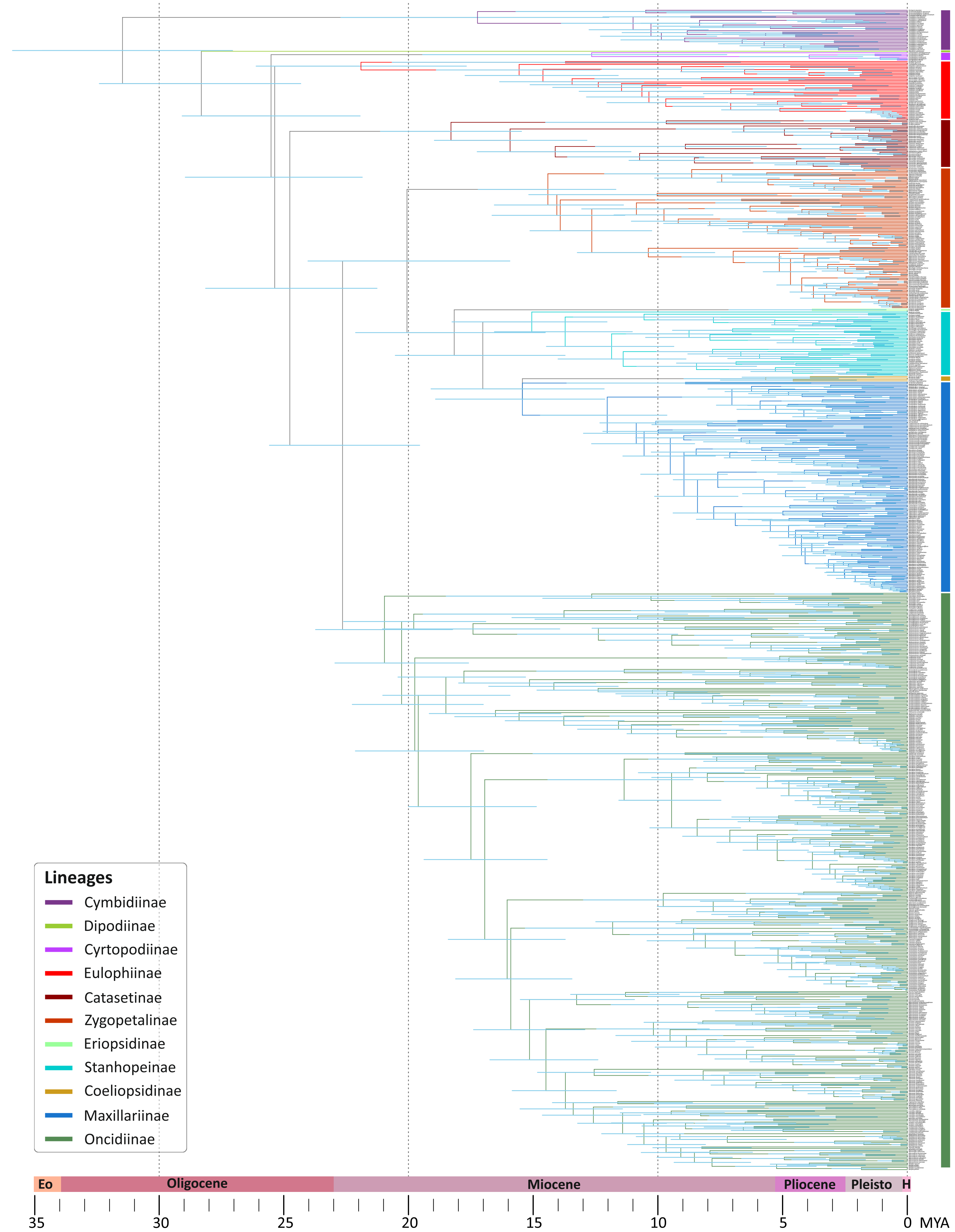

Figure S8. Chronogram of the Cymbidieae obtained under a lognormal relaxed clock model

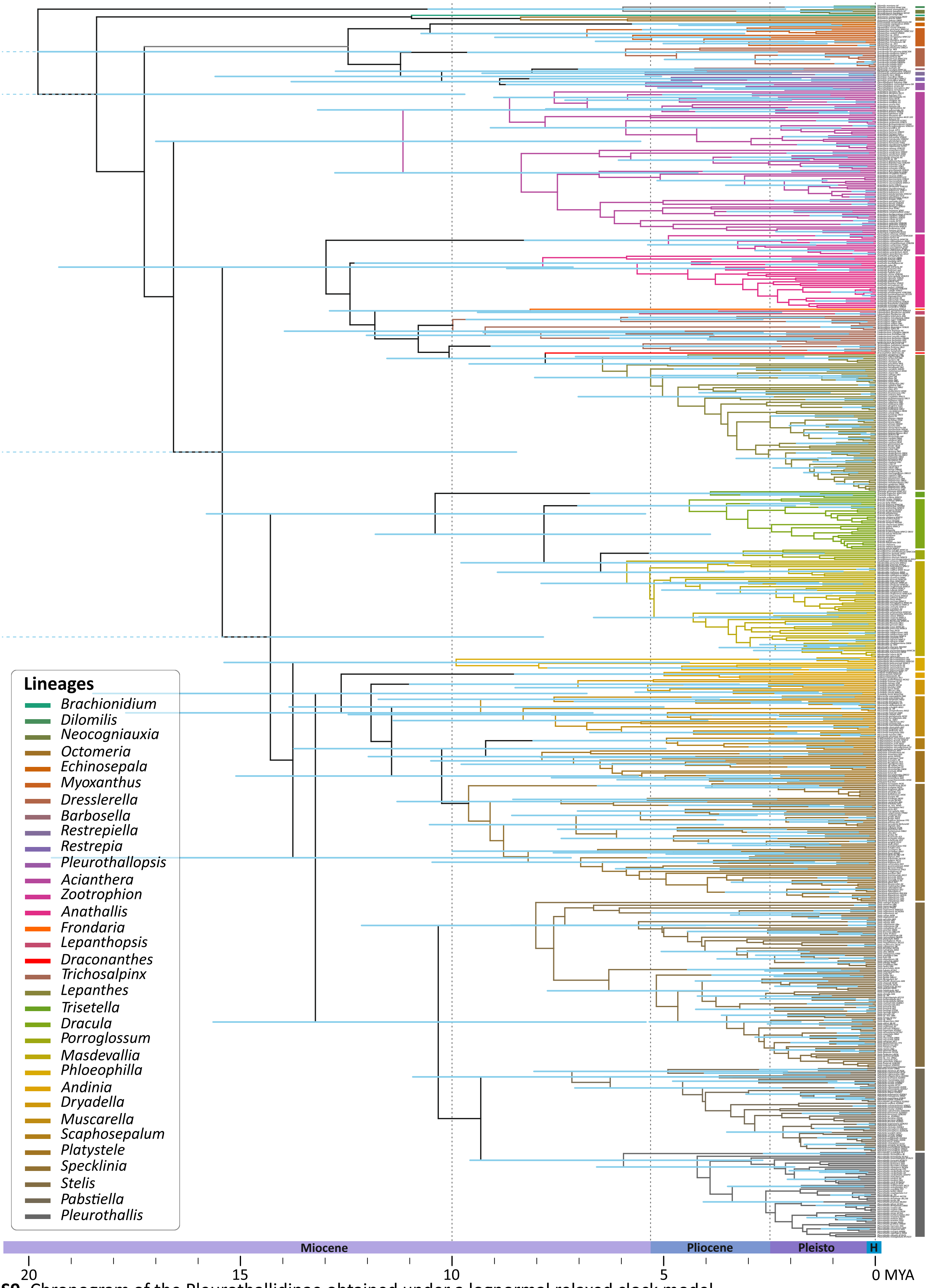

Figure S9. Chronogram of the Pleurothallidinae obtained under a lognormal relaxed clock model

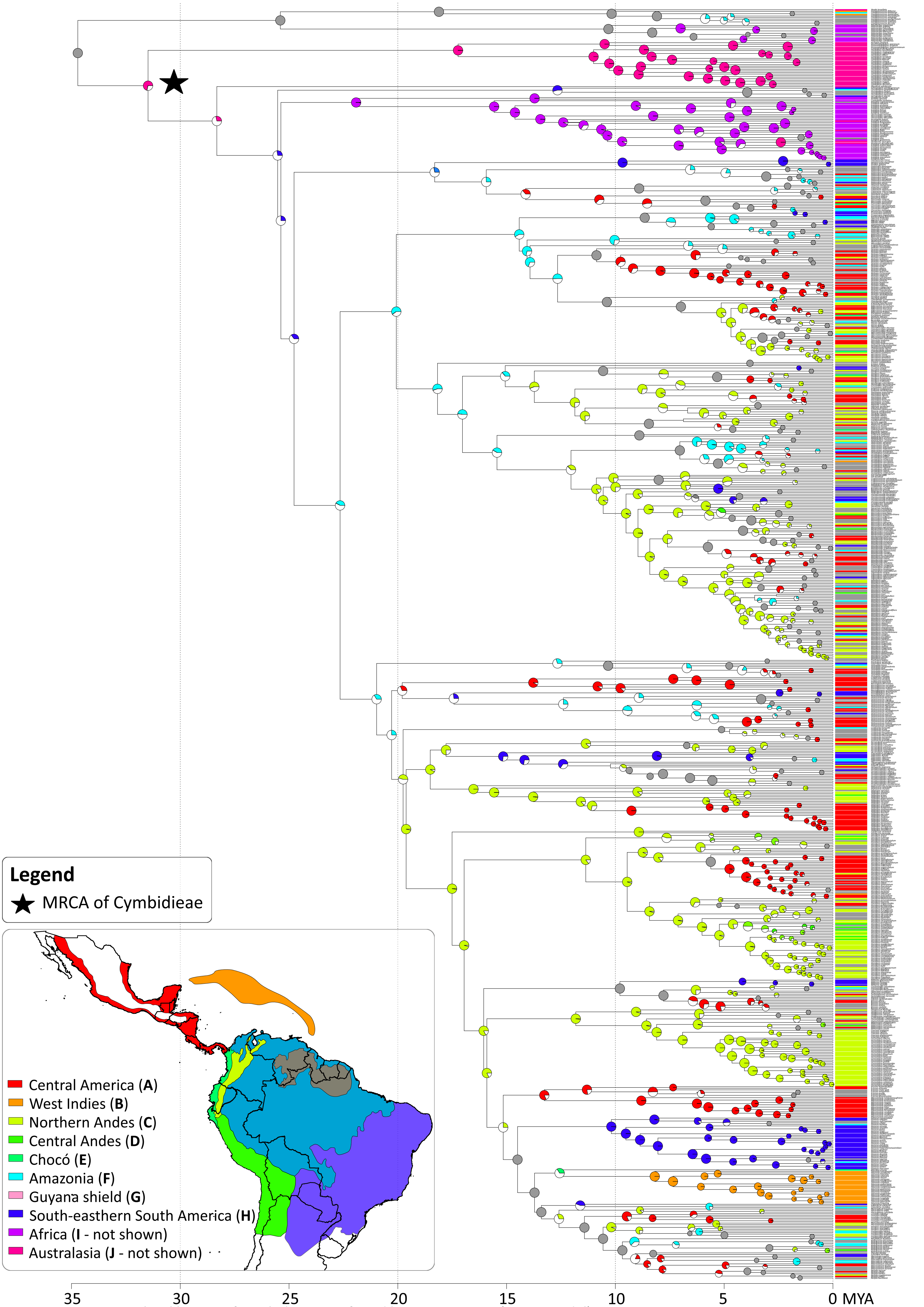

Figure S10. Biogeographical history of Cymbidieae as inferred by BioGeoBEARS (DEC+J model)

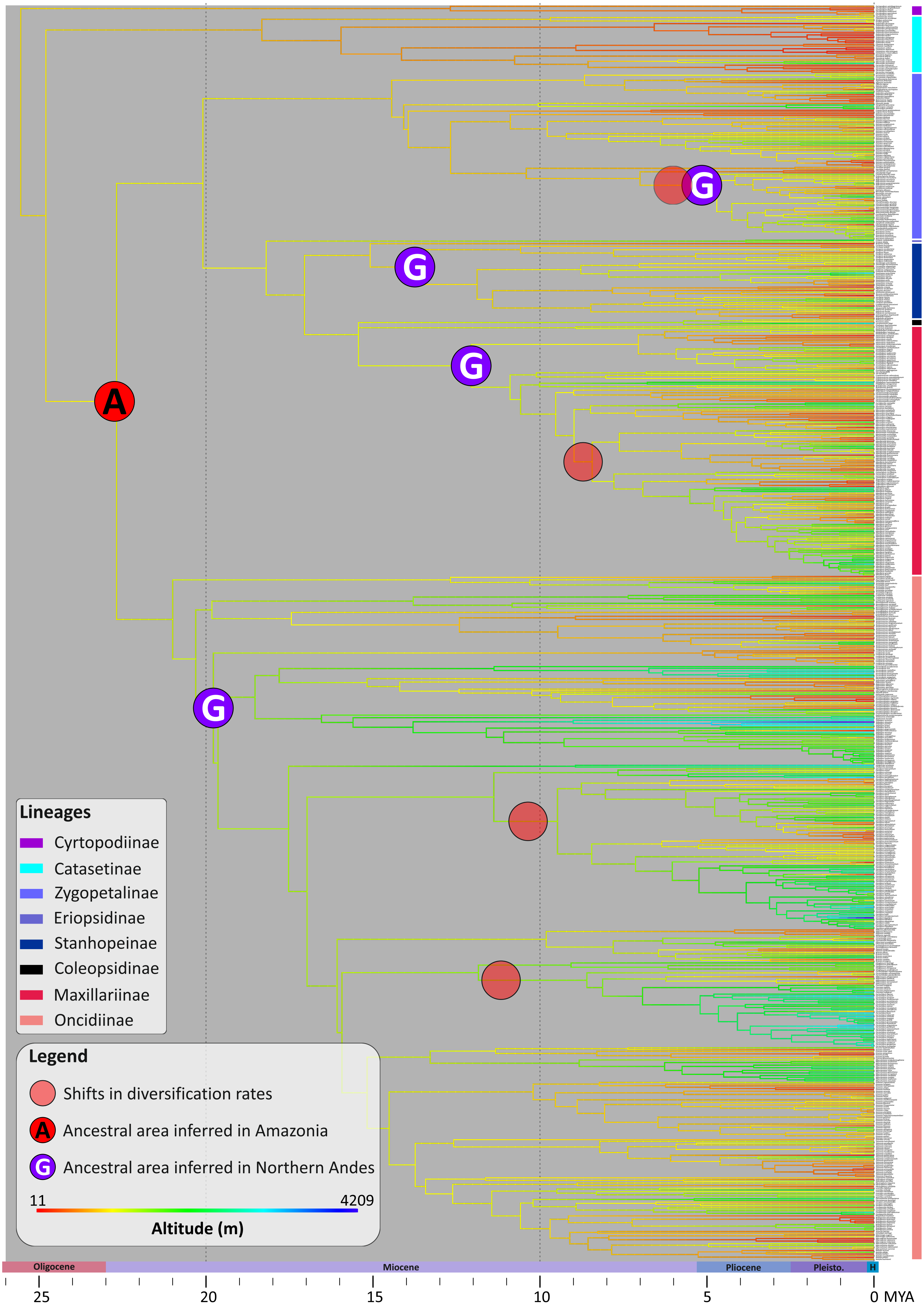

Figure S11. Ancestral character reconstruction of altitude as a continuous character in Cymbidieae

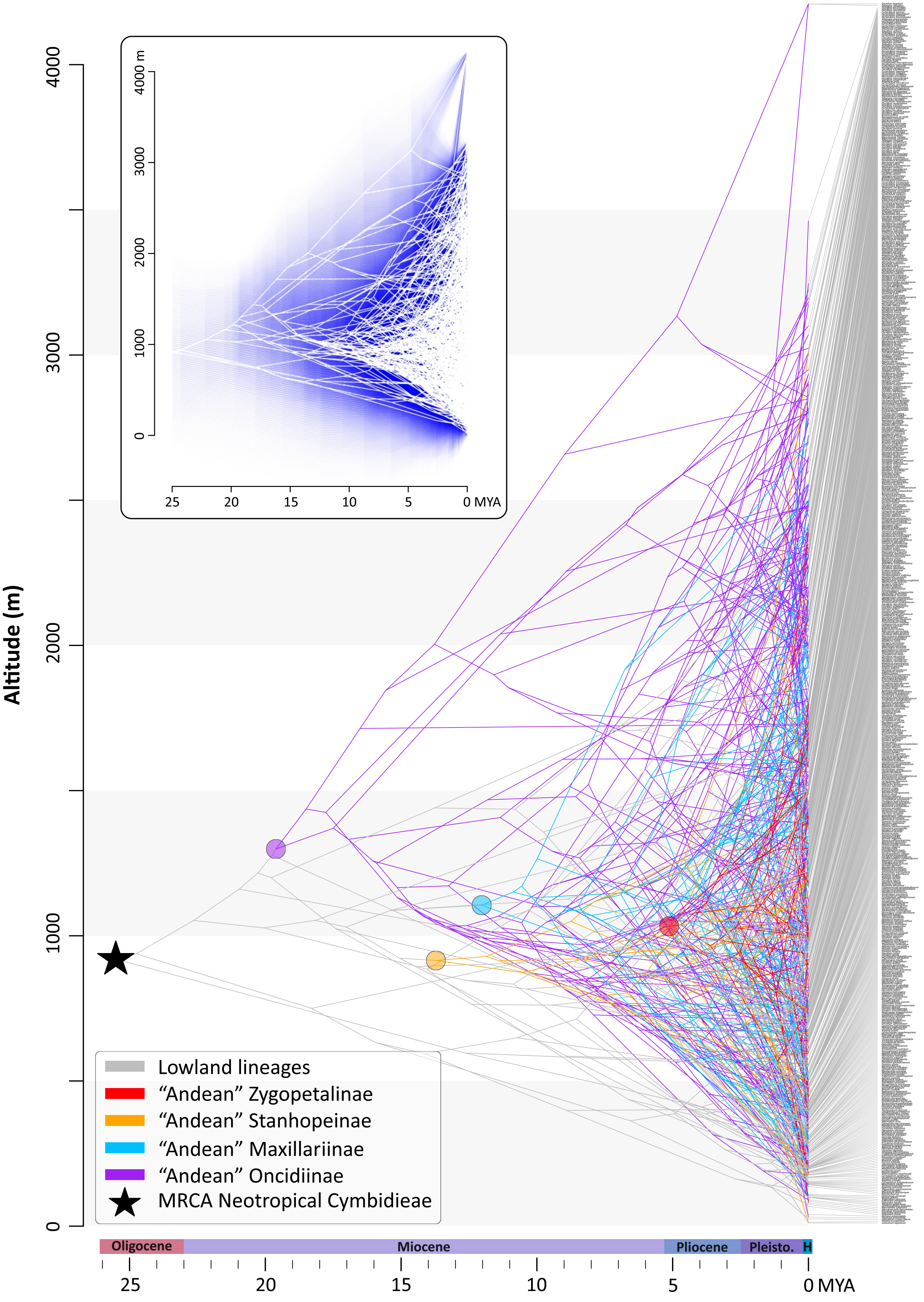

Figure S12. Traitgram of altitude change as a function of time in Cymbidieae

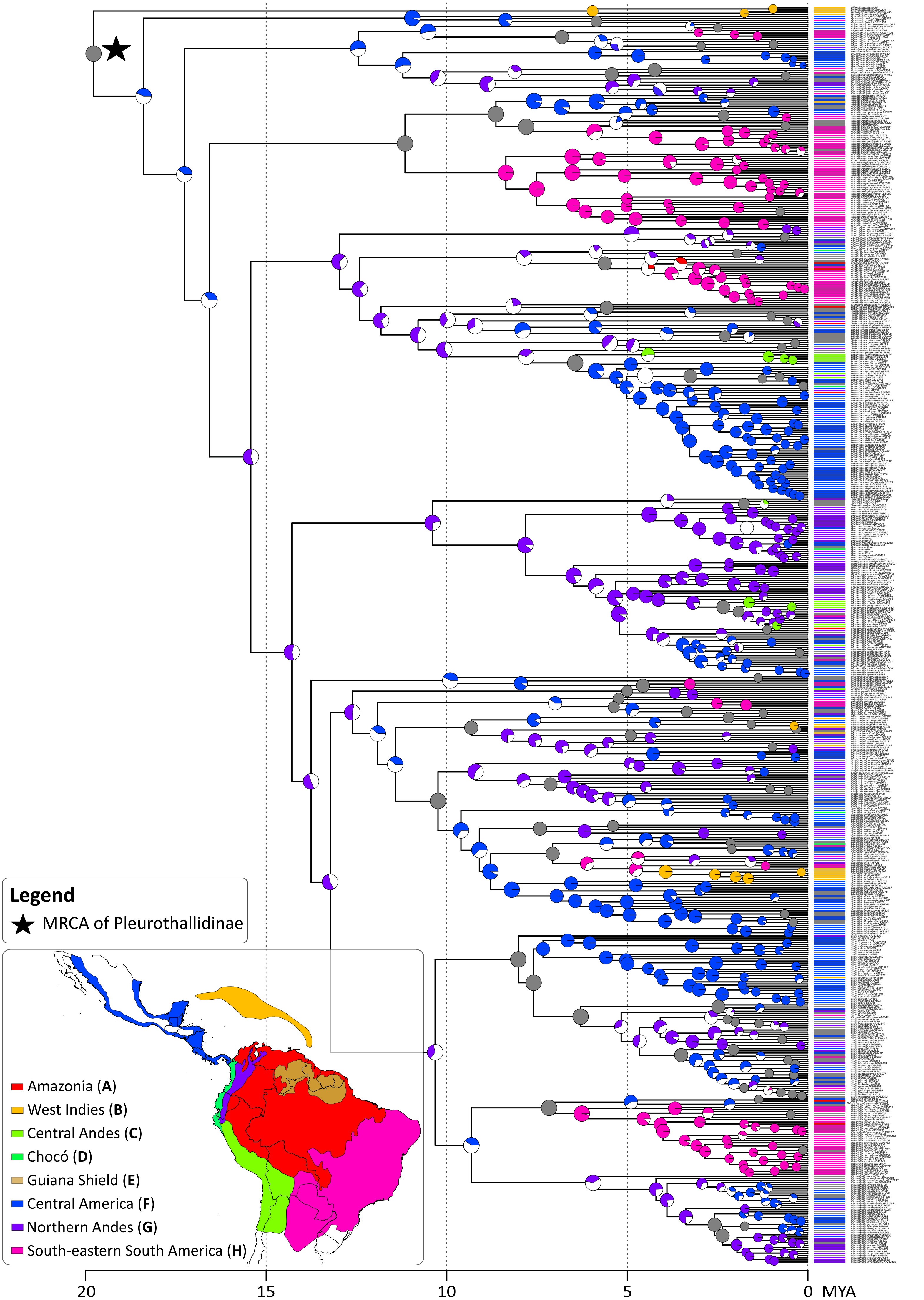

Figure S13. Biogeographical history of Pleurothallidinae as inferred by BioGeoBEARS (DEC+J model)

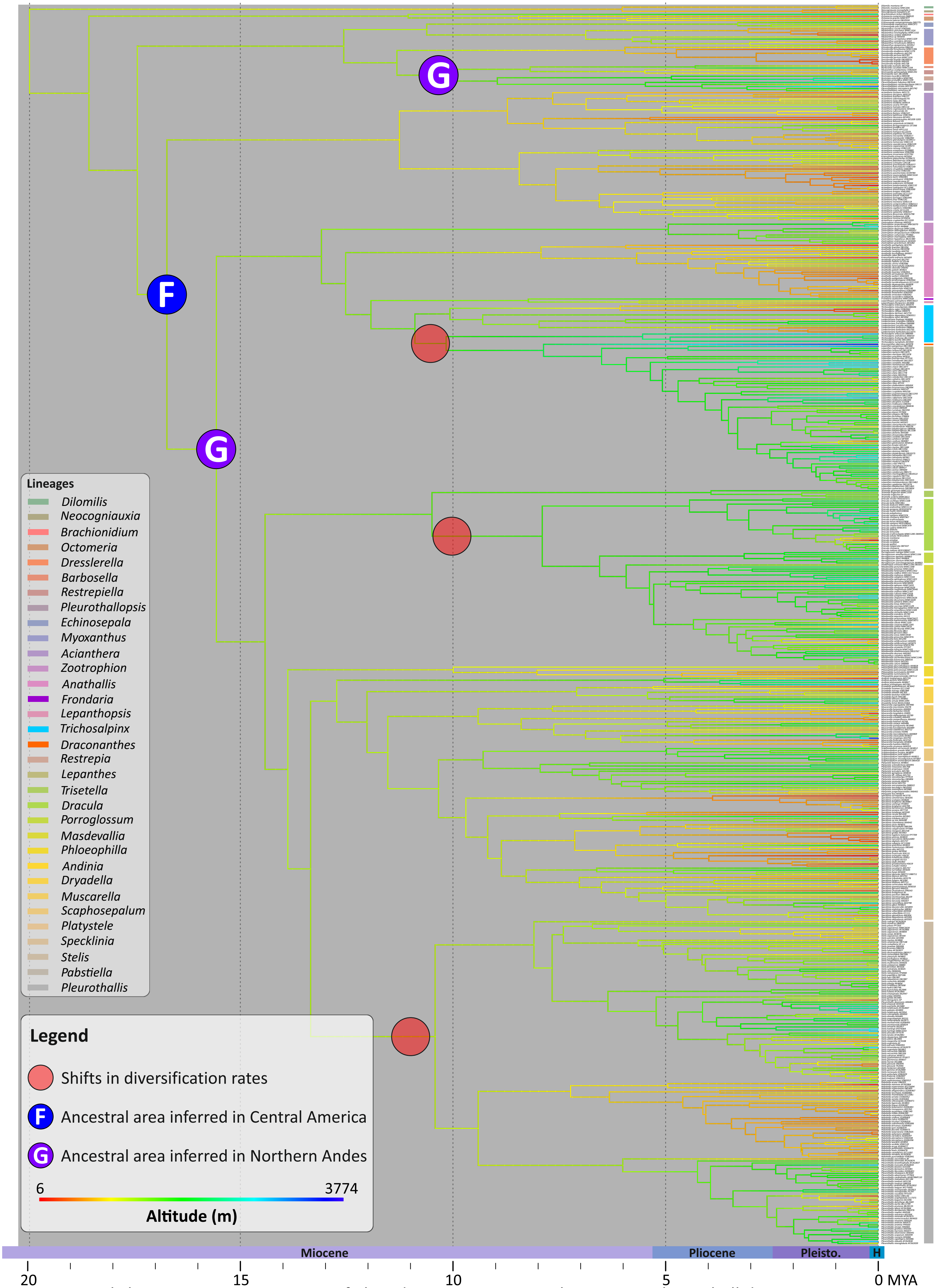

Figure S14. Ancestral character reconstructioon of altitude as a continuous character in Pleurothallidinae

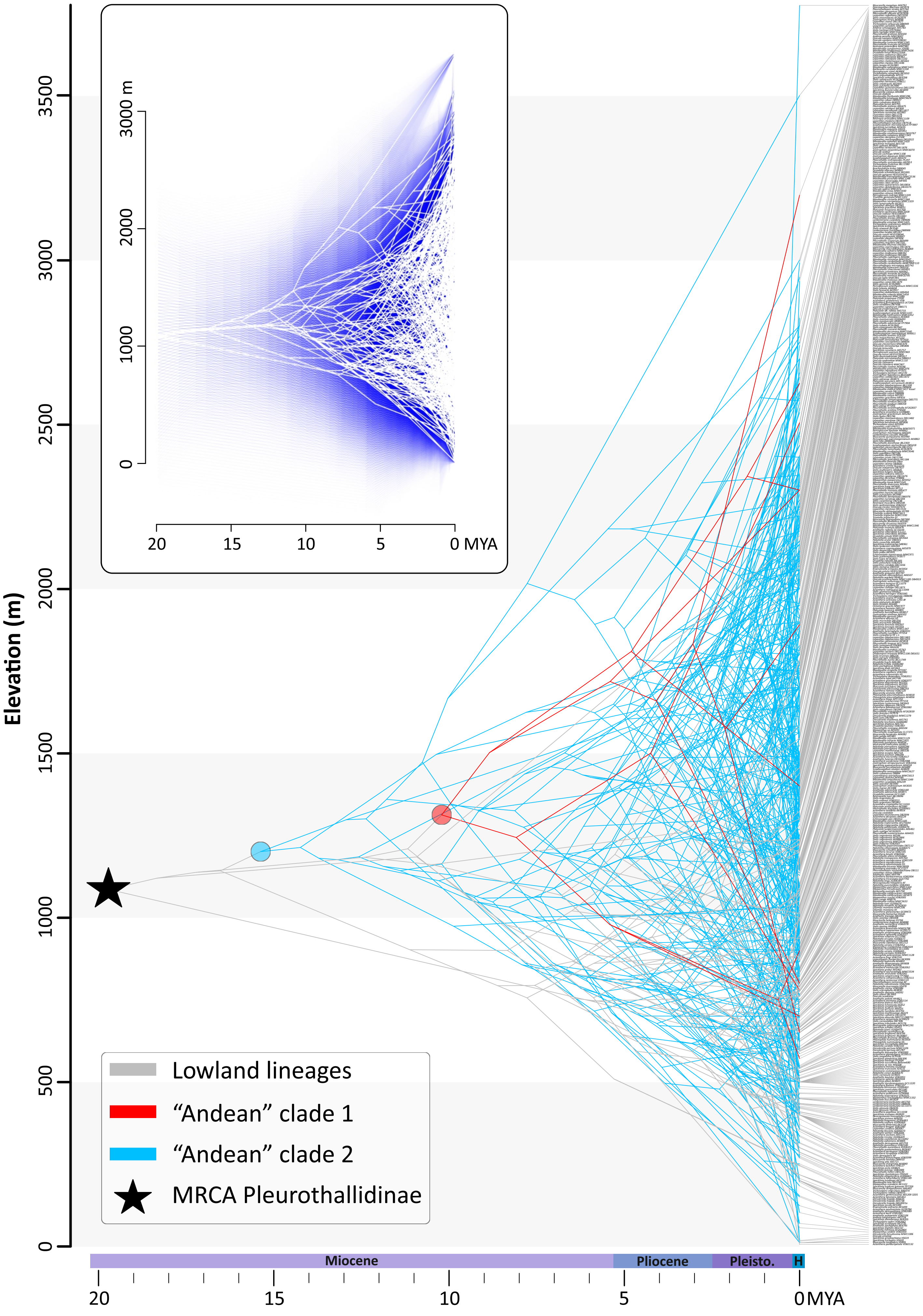

Figure S15. Traitgram of altitude change as a function of time in Pleurothallidinae

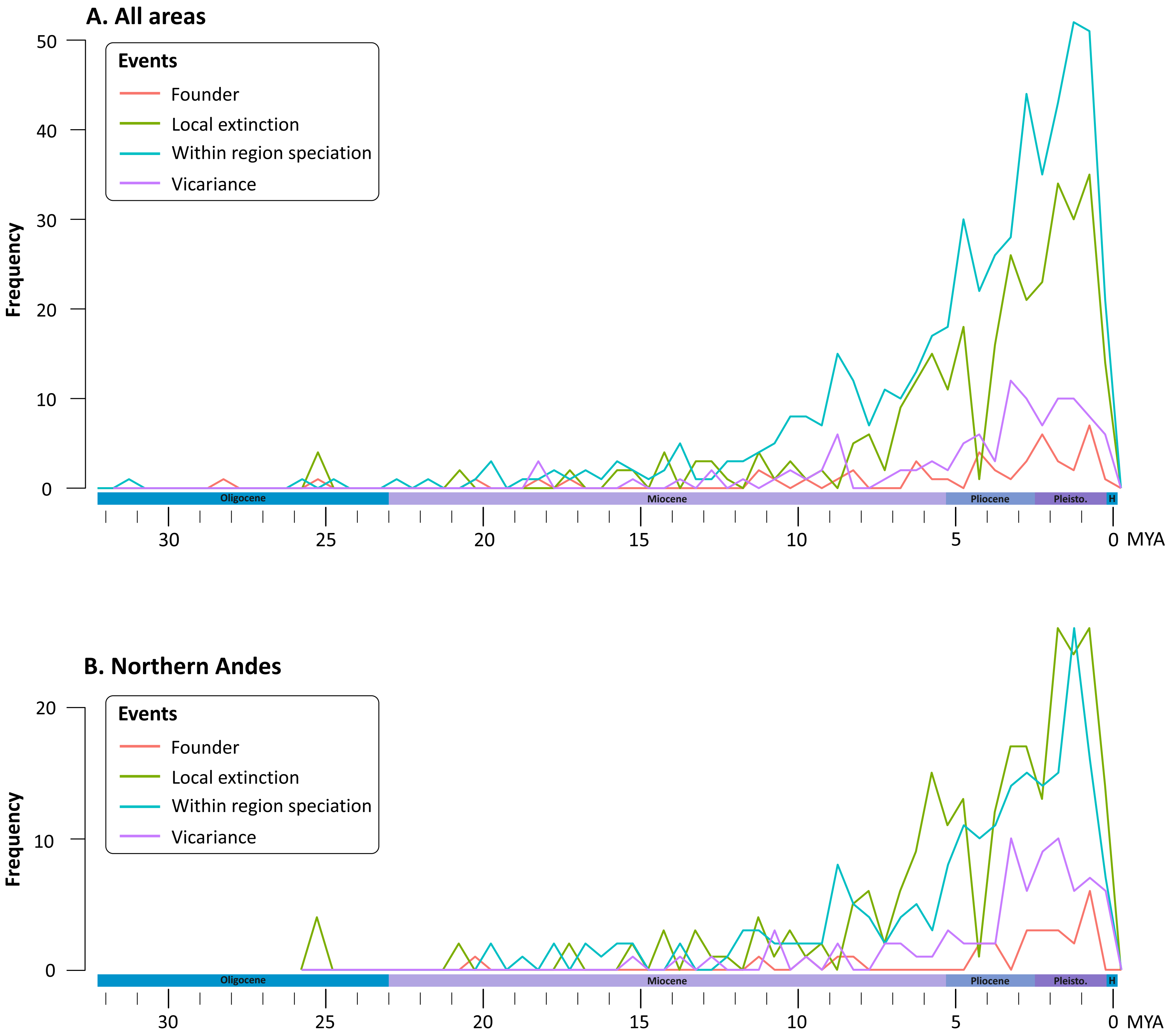

Figure S16. Frequency of relevant biogeographical events through time in Cymbidieae as inferred by Biogeographical Stochastic Mapping.

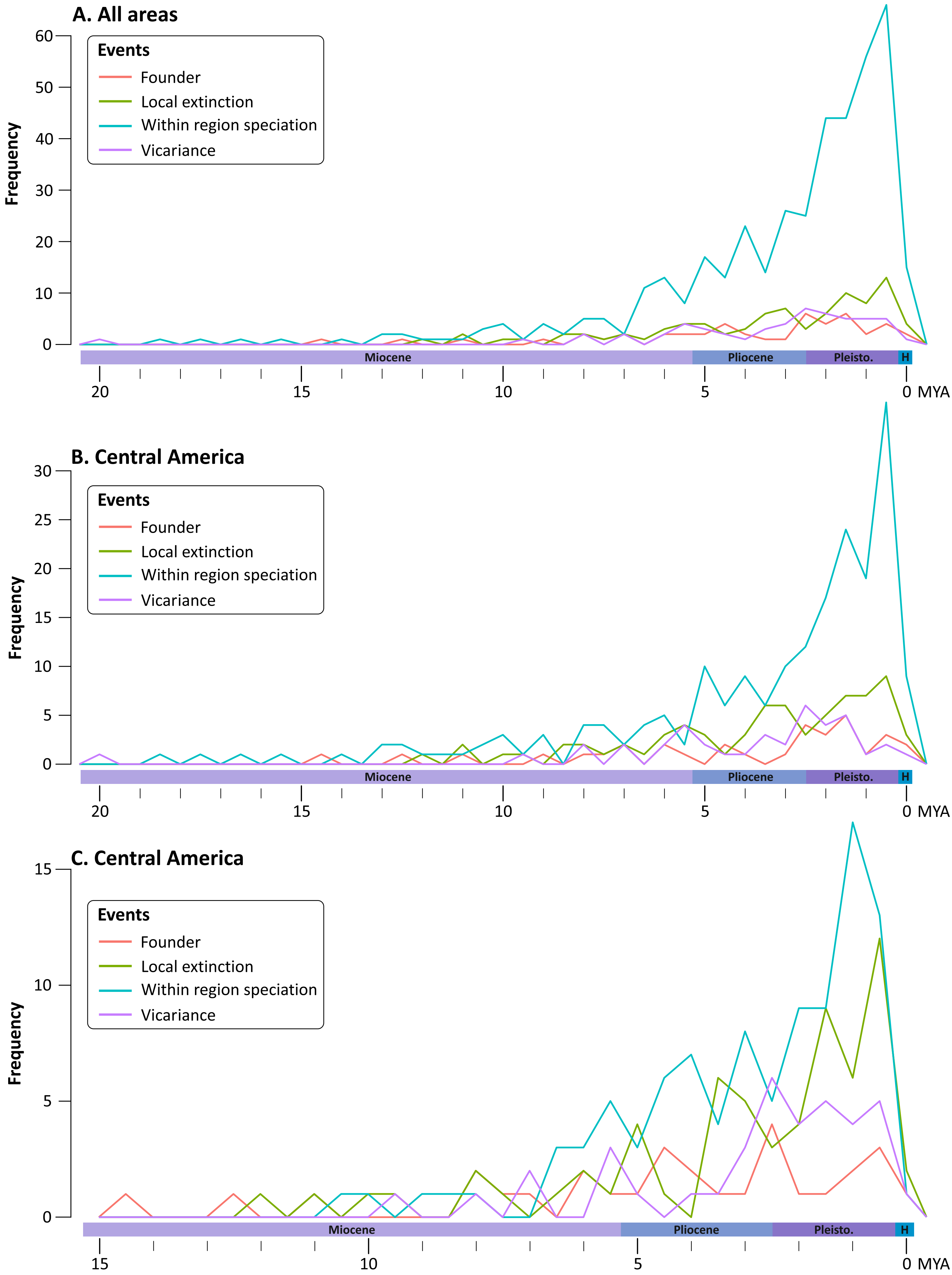

Figure S17. Frequency of relevant biogeographical events through time in Pleurothallidinae as inferred by Biogeographical Stochastic Mapping.

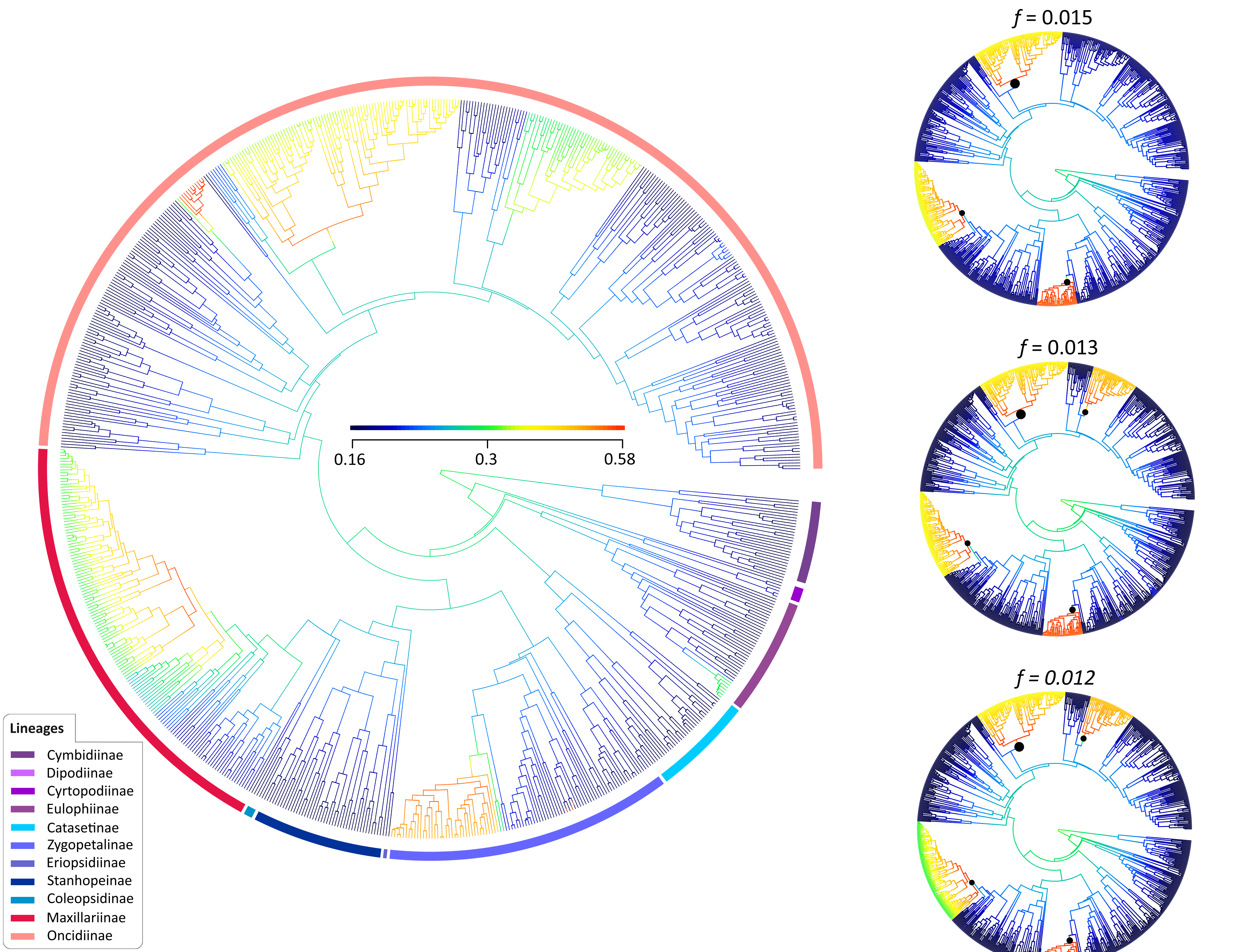

Figure S18. Phylorate plot of Cymbidieae and the three best configuration shifts in diversification rates

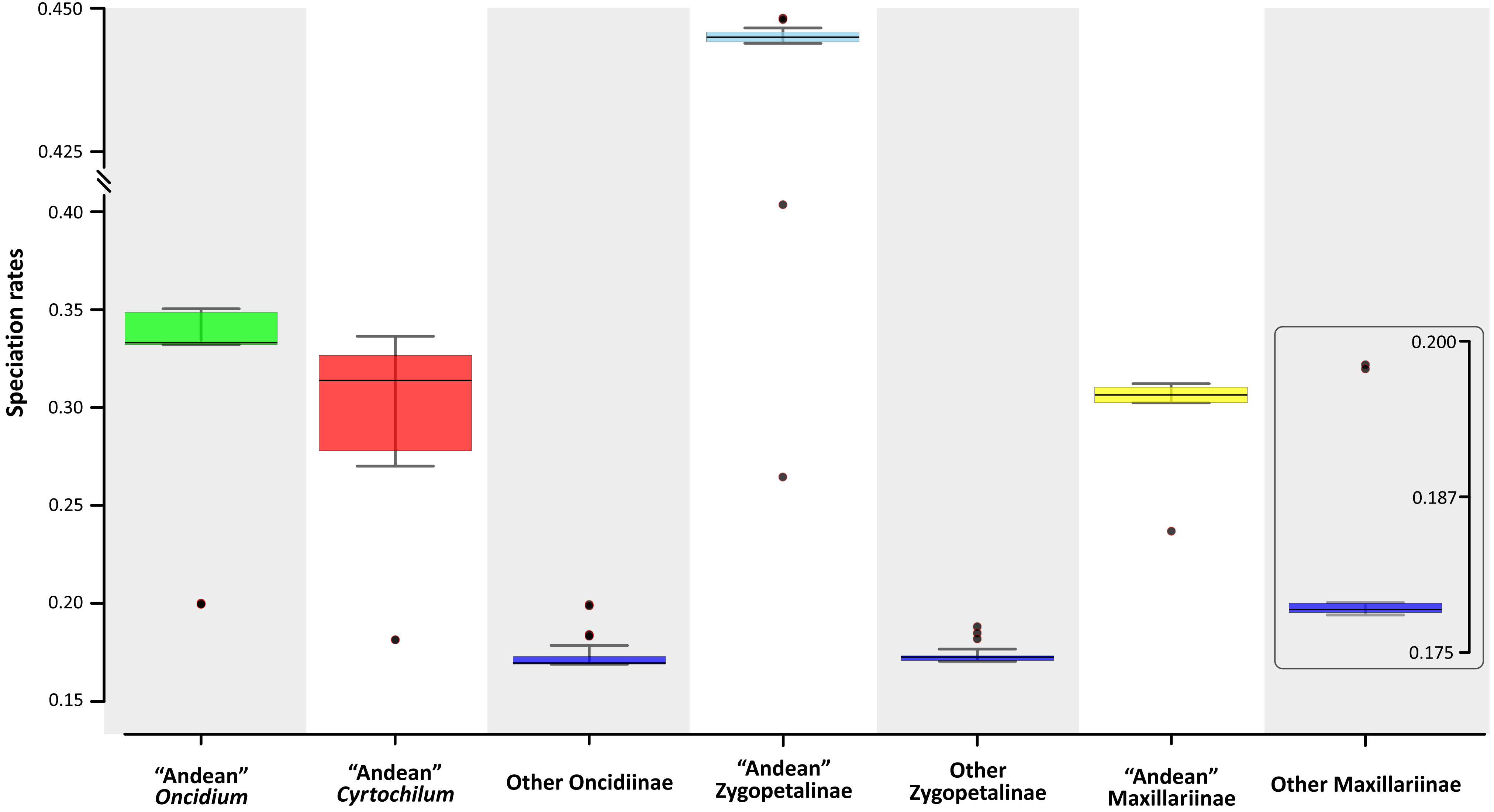

Figure S19. Tip speciation rates of Cymbidieae clades with diversification rate shifts *vs.* sister clades (no diversification rate shifts) as inferred in BAMM

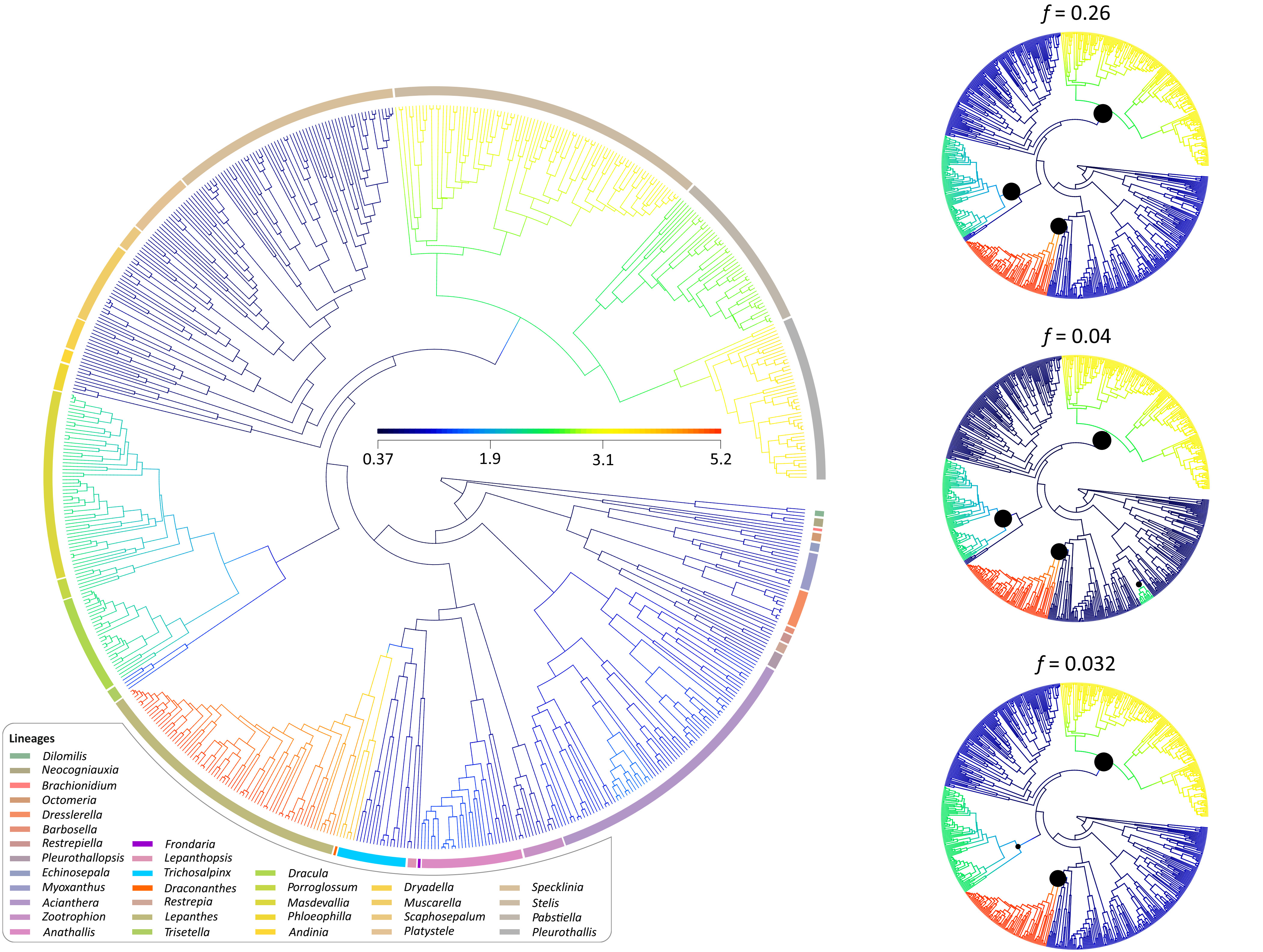

Figure S20. Phylorate plot of Pleurothallidinae and the three best configuration shifts in diversification rates

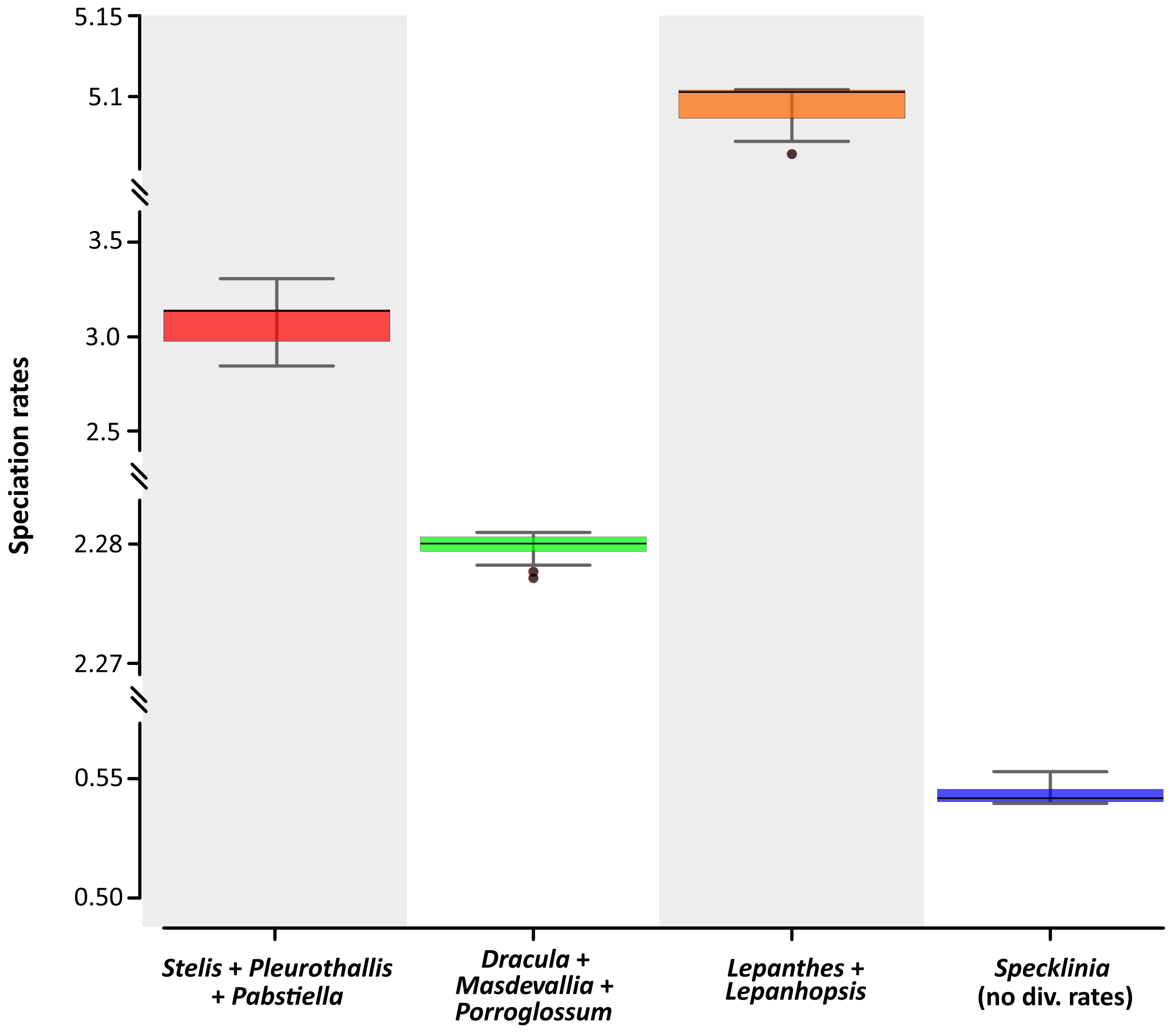

Figure S21. Tip speciation rates of Pleurothallidinae clades with diversification rate shifts *vs.* sister clades (no diversification rate shifts) as inferred in BAMM

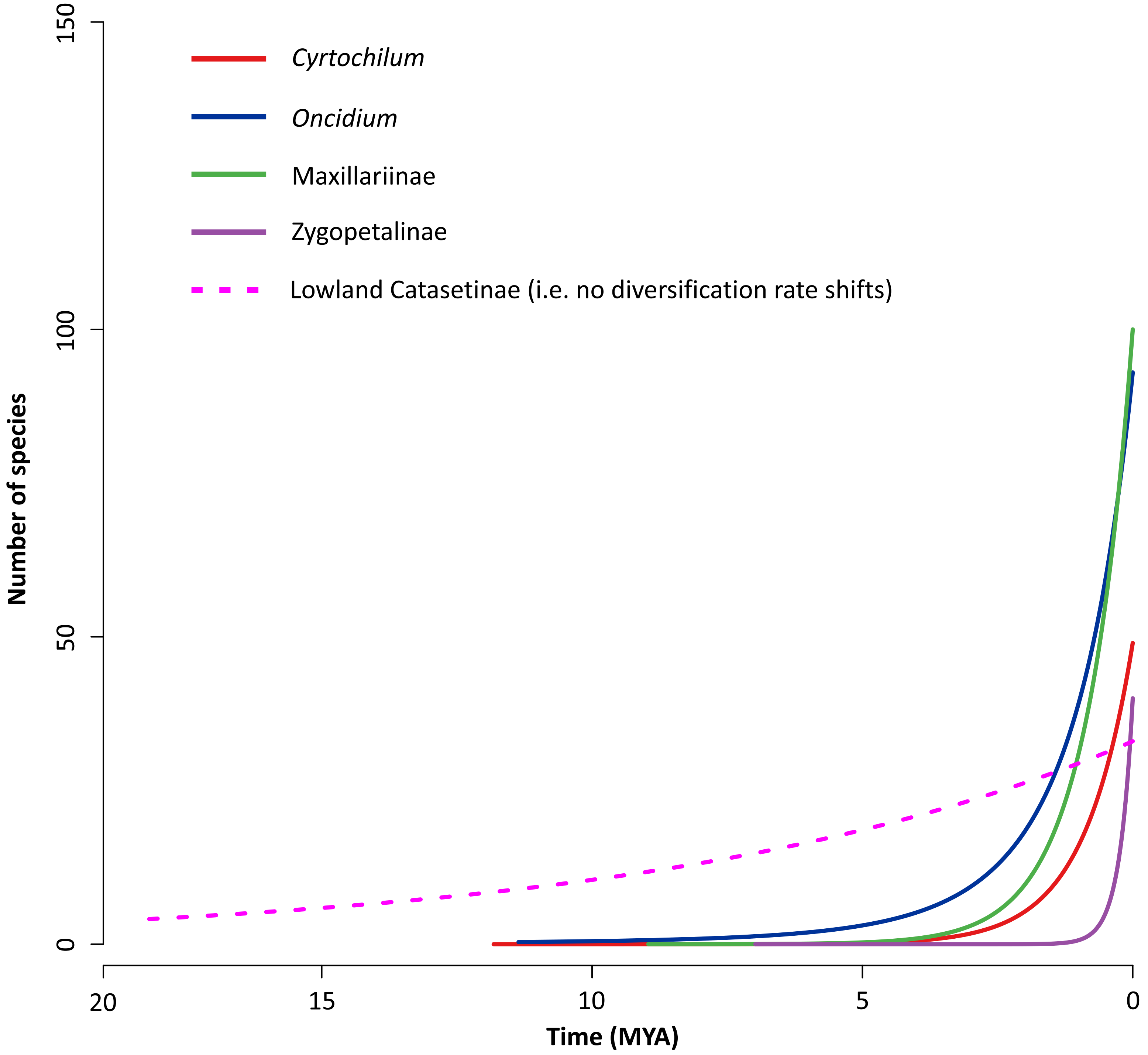

Figure S22. Diversity through time of clades with diversification rate shifts *vs.* Catasetinae (no diversification rate shifts)

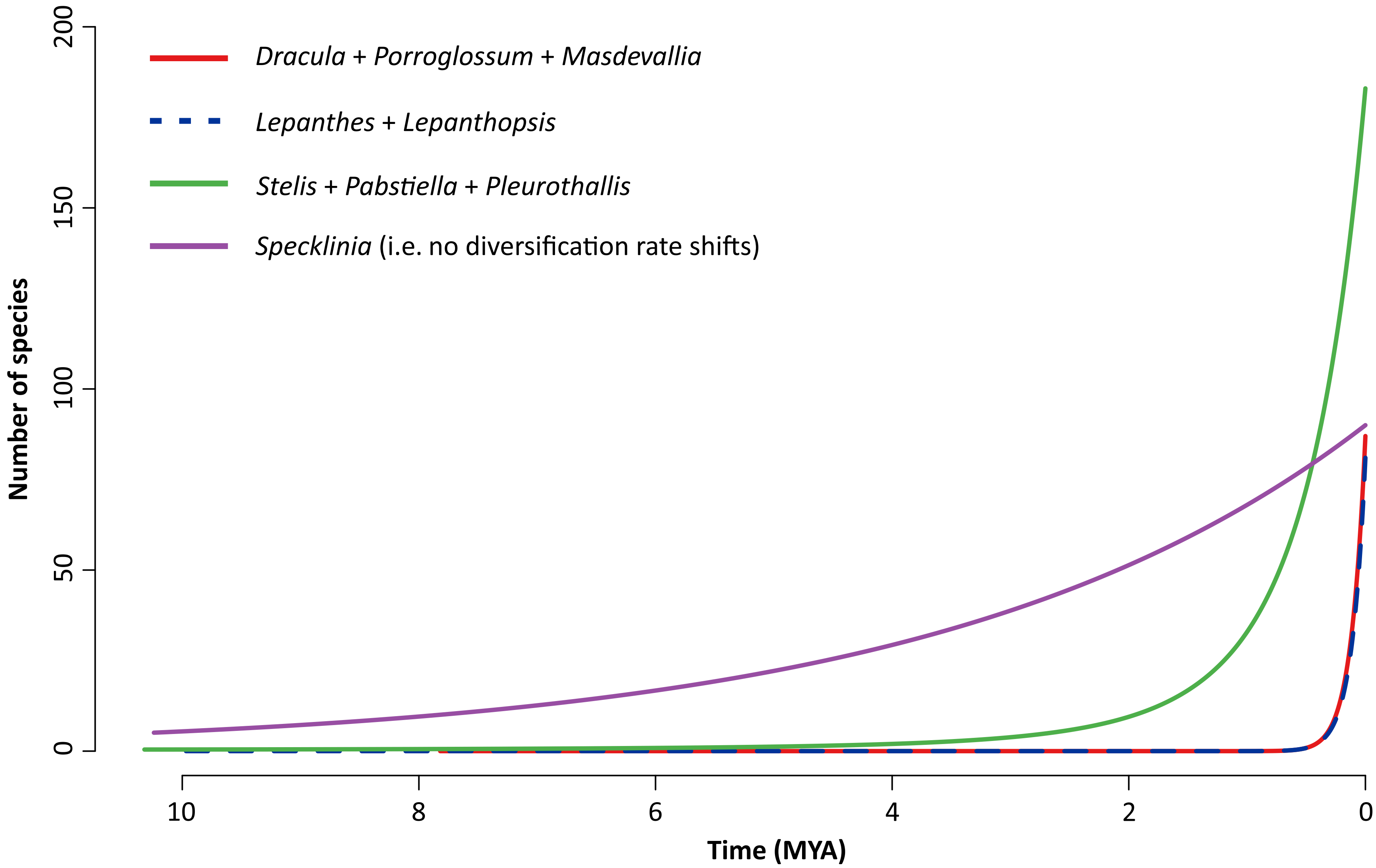

Figure S23. Diversity through time of clades with diversification rate shifts *vs. Specklinia* (no diversification rate shifts)

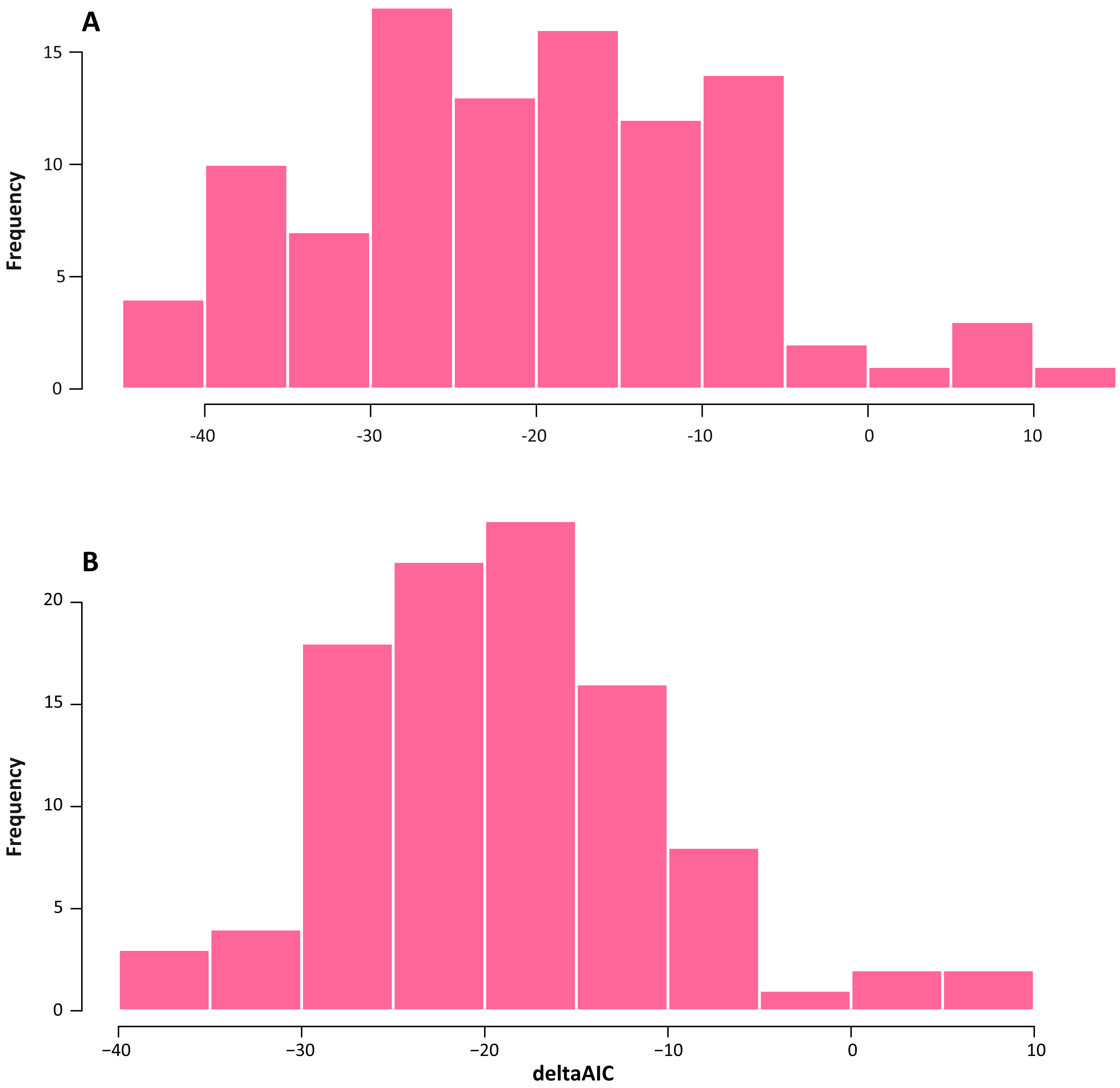

Figure S24. Null distribution of dAIC obtained from randomly shuffled geographic distribution data of A) Cymbidieae and B) Pleurothallidinae

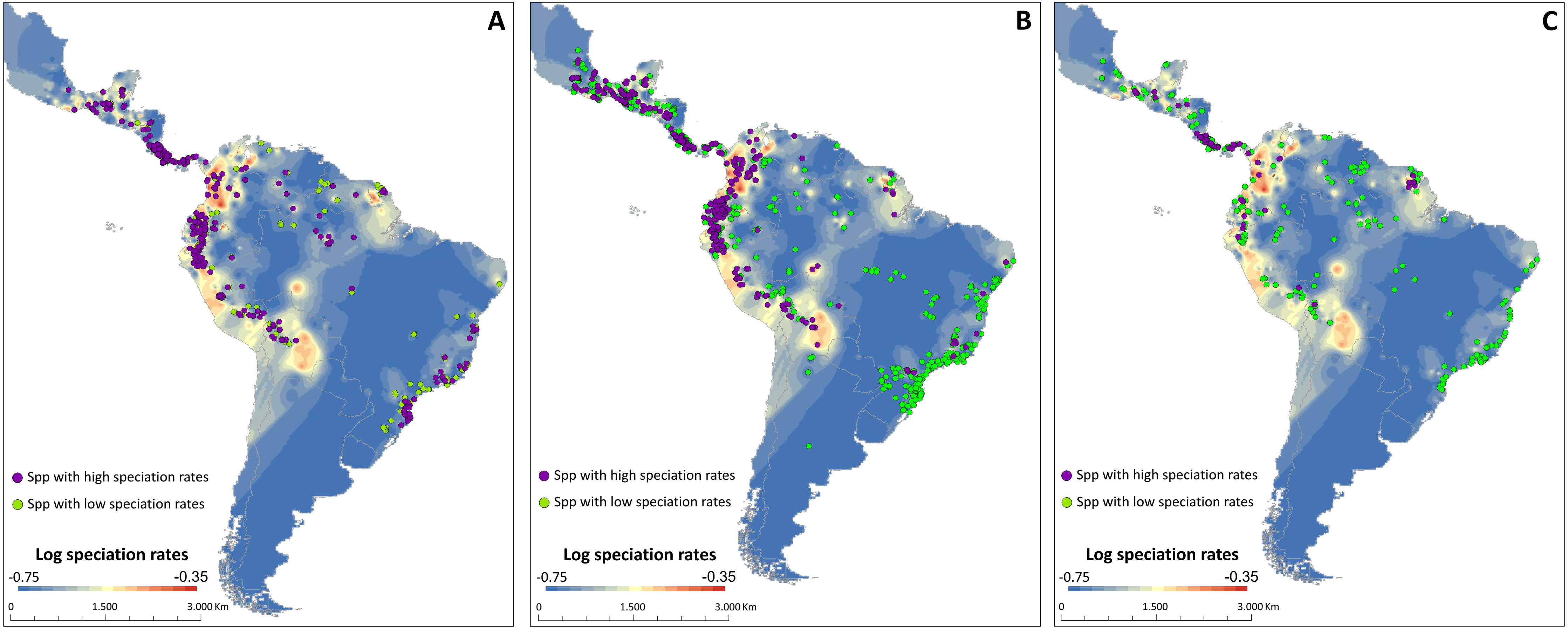

Figure S25. Distribution of Cymbidieae species included in clades with high and low speciation rates. **A**) Maxillariinae; **B**) Oncidiinae; **C**) Zygopetalinae

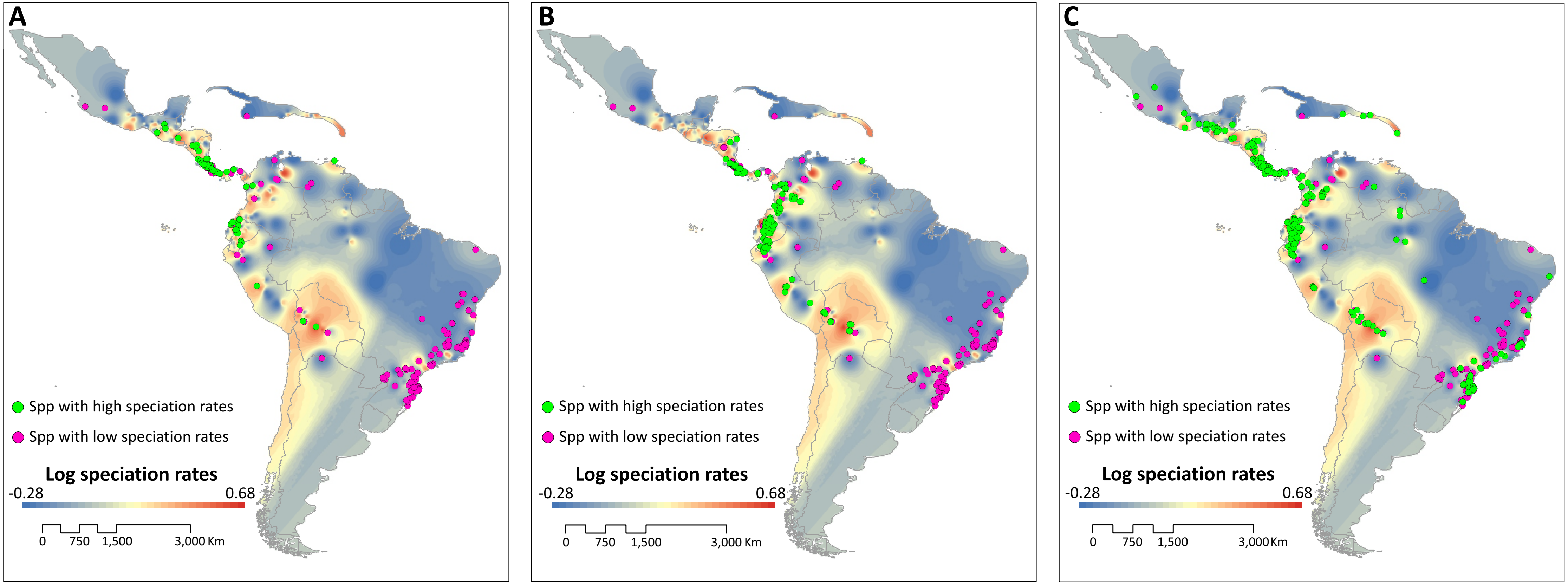

Figure S26. Distribution of Pleurothallidinae species included in clades with high and low speciation rates (i.e. *Acianthera*). **A**) *Lepanthes* + *Lepanthopsis*; **B**) *Masdevallia* + *Dracula* + *Porroglossum*; **C**) *Pabstiella* + *Pleurothallis* + *Stelis*

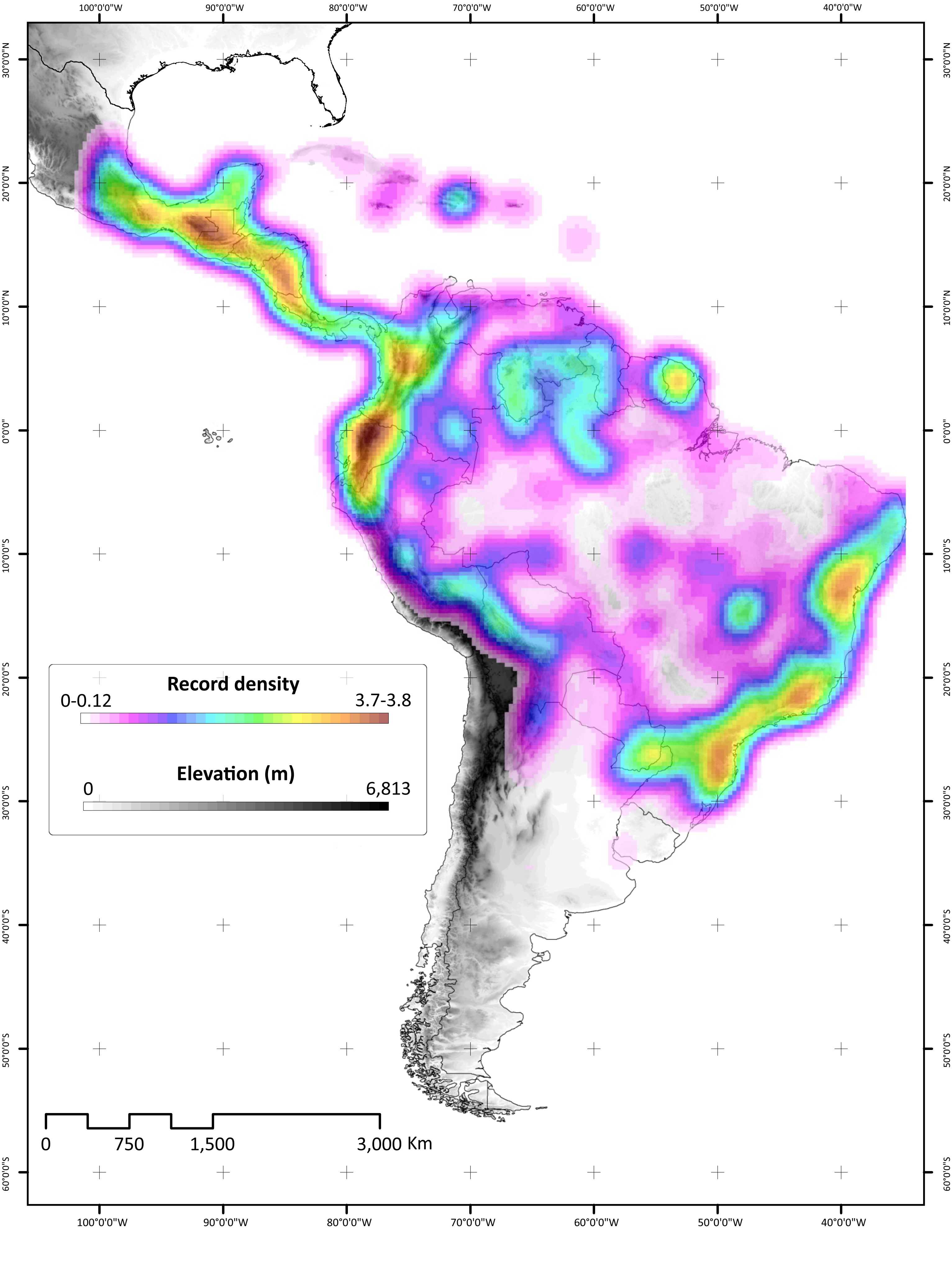

Figure S27. Density record map of Cymbidieae (occurrences/grid cell; ~1900 records)

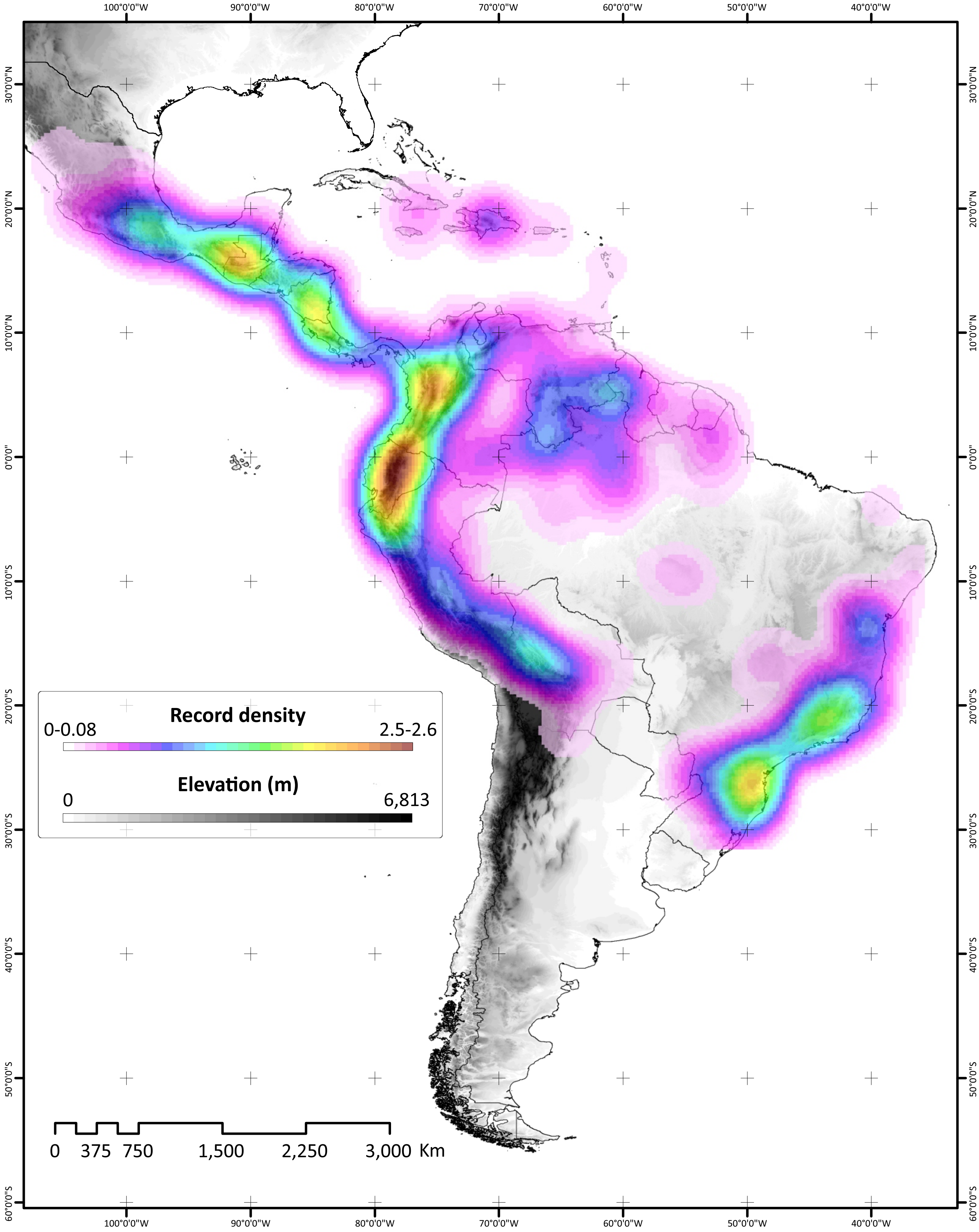

Figure S28. Density record map of Pleurothallidinae (occurrences/grid cell; ~700 records)

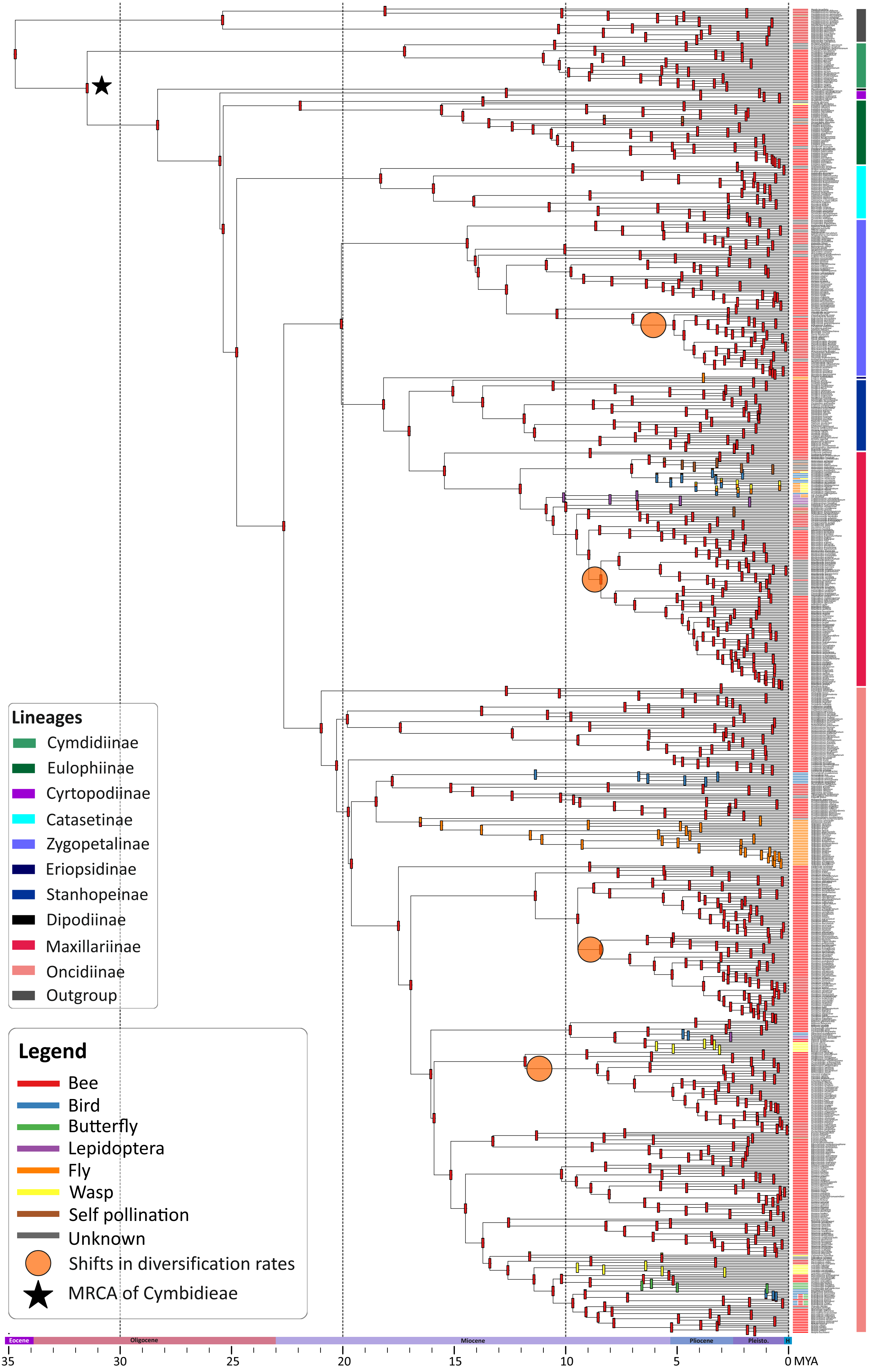

Figure S29. Ancestral character reconstruction of pollination syndromes of Cymbidieae

Table S1

Scientific Name	ITS	*mat* K	*psb* A	*trn* L-F	*ycf1*
Acineta superba	745672340	13603647	-	745672403	564292250
Acriopsis javanica	22087059	22087027	-	-	-
Acrolophia bolusii	570759250	659898138	-	-	-
Aetheorhyncha andreettae	58042649	58200520	-	-	564291976
Aganisia fimbriata	58042723	58200668	-	58042625	564292012
Aganisia pulchella	58042724	58200670	-	58042626	564292010
Ansellia africana	22087058	126789232	-	-	-
Aspasia epidendroides	13492692	15004382	224967628	13492771	224981872
Aspasia lunata	126789407	126789173	224967631	223370507	224981874
Batemannia colleyi	8699095	13603575	-	9789375	-
Batemannia lepida	58042707	58200636	-	-	564291850
Benzingia cornuta	58042644	58200510	-	58042558	564292104
Benzingia reichenbachiana	-	167889473	-	9789357	564291930
Braemia vittata	8699132	13603649	-	9789412	564291880
Brasiliorchis gracilis	78095047	78094364	-	-	564292056
Brasiliorchis schunkeana	115394628	78094524	-	-	564292046
Brassia allenii	13492690	15004378	224968219	13492769	224982264
Brassia andina	223371620	223371109	224969098	223370549	224997968
Brassia arcuigera	13492693	13603695	224968534	13492772	224982464
Brassia aurantiaca	13492689	13603681	224968552	13492768	224997606
Brassia caudata	13492694	167889858	224967634	13492773	-
Brassia horichii	223371392	167889670	224968261	223370518	224997411
Calypso bulbosa	21780260	154968253	-	33325705	-
Camaridium bradeorum	78095382	78094653	-	-	564292140
Camaridium cucullatum	115394610	78094433	-	-	564291912
Camaridium nutantiflorum	78095389	78094835	-	9789363	564292142
Camaridium vestitum	78094929	167889662	-	-	564291812
Campylocentrum fasciola	28376254	28376304	-	27447629	-
Campylocentrum filiforme	28376256	28376306	-	28376283	-

Campylocentrum jamaicense	28376259	28376314	-	28376286	-
Campylocentrum lansbergii	28376257	28376308	-	28376284	-
Campylocentrum micranthum	27447622	167889479	-	27447630	187372607
Campylocentrum pachyrrhizum	28376261	28376316	-	28376287	-
Campylocentrum poeppigii	28376262	28376320	-	28376289	-
Campylocentrum sullivanii	71912783	71913002	-	71912664	-
Campylocentrum tyrridion	28376265	71913004	-	71912665	-
Capanemia superflua	13492720	15004438	224967305	13492799	224981662
Catasetum collare	937352664	-	-	-	937352933
Catasetum expansum	-	564291804	-	-	564292254
Catasetum macrocarpum	937352668	-	-	-	937352937
Catasetum x roseoalbum	937352671	-	-	-	937352939
Caucaea andigena	223371540	223371023	224968834	-	224982660
Caucaea nubigena	17182112	223371010	224968795	-	224982634
Caucaea olivacea	17182114	223371034	224968867	17182276	224997814
Caucaea phalaenopsis	17182108	17182215	224967296	17182277	224981658
Caucaea radiata	17182107	17182217	224968120	17182278	224982198
Chaubardia klugii	58042690	58200602	-	58042602	564291960
Chaubardia surinamensis	58042691	58200604	-	58042603	564291822
Chaubardiella pubescens	58042661	58200544	-	58042573	564292036
Chaubardiella subquadrata	58042662	58200546	-	58042574	564292018
Chaubardiella tigrina	58042663	58200548	-	58042575	564291826
Chondrorhyncha rosea	58042631	58200484	-	-	564291974
Chondroscaphe amabilis	58042683	58200588	-	58042595	564291986
Chondroscaphe eburnea	58042632	58200486	-	58042546	564292020
Chondroscaphe flaveola	58042686	58200594	-	58042598	564291844
Christensonella echinophyta	78095136	156901521	-	156708209	-
Christensonella ferdinandiana	78095068	78094385	-	156708192	564291910
Christensonella nardoides	78095274	78094558	-	156708219	564292108
Christensonella pacholskii	78095294	78094576	-	156708218	564292078
Christensonella pumila	78095022	78094387	-	156708210	-
Christensonella subulata	78095240	78094459	-	156708214	-

Chytroglossa marileoniae	78095240	223371133	224969176	223370555	224982880
Cirrhaea fuscolutea	745672356	564291786	-	745672416	564292220
Cirrhaea seidelii	745672358	564291748	-	745672417	564291870
Cischweinfia colombiana	223371485	223370970	224968648	-	224982536
Cischweinfia dasyandra	13492698	13603693	224967676	13492777	224981904
Cischweinfia jarae	223371382	225580278	224968222	-	224982266
Clowesia dodsoniana	-	564291802	-	-	564292252
Clowesia russelliana	937352672	-	-	-	937352941
Cochleanthes flabelliformis	58042682	58200586	-	58042594	564292230
Coeliopsis hyacinthosma	8699096	126789105	167890792	9789376	564291878
Comparettia barkeri	223371363	223370853	-	-	224982200
Comparettia falcata	223371283	167889493	224969104	223370550	224982834
Comparettia langkastii	223371481	225580320	224968636	223370530	224982528
Comparettia macroplectron	13492712	15004422	224967433	13492791	224981748
Comparettia schaeferi	223371358	225580260	224968099	223370515	224982184
Coryanthes elegantium	8699112	13603609	-	9789392	564291840
Coryanthes macrantha	8699111	13603607	-	9789391	564291842
Cryptarrhena guatemalensis	58042700	58200622	-	58042612	564292156
Cryptarrhena lunata	58042699	13603537	-	58042611	161597648
Cryptocentrum calcaratum	78095160	13603549	-	9789362	-
Cryptocentrum peruvianum	78095218	78094511	-	-	564292064
Cryptocentrum pseudobulbosum	78095219	78094586	-	-	-
Cryptocentrum standleyi	78095248	78094533	-	-	-
Cuitlauzina candida	126789418	126789190	224967487	223370563	224981786
Cuitlauzina egertonii	223371284	225580138	224967715	223370508	224981930
Cuitlauzina pendula	223371242	126789191	224967505	223370506	224981798
Cuitlauzina pulchella	223371215	223370731	224967287	-	224981652
Cyanaeorchis arundinae	576890493	576890497	-	-	973601556
Cyanaeorchis minor	576890495	576890501	-	-	-
Cycnoches cooperi	937352678	564291806	-	-	564292256
Cycnoches egertonianum	756808976	167889956	-	-	937352955
Cycnoches lehmannii	937352689	564291736	-	-	564291860

Cycnoches manoelae	937352692	564291712	-	-	564291824
Cycnoches pachydactylon	937352693	564291720	-	-	564291836
Cymbidiella pardalina	22087056	22087024	-	-	-
Cymbidium atropurpureum	22087064	22087030	-	-	-
Cymbidium bicolor	21427017	295147555	-	-	-
Cymbidium cyperifolium	21427040	619498906	-	-	-
Cymbidium devonianum	22087082	22087044	-	-	564291854
Cymbidium eburneum	21427030	22087035	-	-	653816625
Cymbidium ensifolium	11182035	18027045	-	-	-
Cymbidium erythrostylum	22087090	22087048	-	-	-
Cymbidium faberi	11182037	619498890	-	-	-
Cymbidium finlaysonianum	22087081	296916858	-	-	-
Cymbidium floribundum	21427019	22087032	-	-	-
Cymbidium goeringii	11182045	22087047	-	480993406	-
Cymbidium hookerianum	21427025	22087036	-	-	-
Cymbidium insigne	11182032	22087041	-	-	-
Cymbidium iridioides	21427023	22087033	-	-	-
Cymbidium kanran	11182041	480993344	-	480993407	-
Cymbidium lancifolium	11182047	22087040	-	-	-
Cymbidium lowianum	11182033	22087042	-	-	-
Cymbidium madidum	22087060	22087038	-	-	-
Cymbidium maguanense	619498876	619498939	-	-	-
Cymbidium rectum	22087061	22087028	-	-	-
Cymbidium serratum	319890852	619498886	-	-	-
Cymbidium sinense	11182034	22087039	-	-	482662084
Cymbidium wenshanense	21427029	619498937	-	-	653816618
Cymbidium whiteae	22087075	22087039	-	-	-
Cyrtidiorchis alata	78095026	78094352	-	-	564291846
Cyrtidiorchis stumpflei	223371250	223370762	224967559	-	224981836
Cyrtochiloides ochmatochila	17182104	17182220	224967442	17182280	224981754
Cyrtochiloides panduriformis	17182106	167889497	224967472	17182281	224981774
Cyrtochiloides riopalenqueana	223371403	223370899	224968339	-	224982344

Cyrtochilum angustatum	13492959	223370752	224967481	-	224981780
Cyrtochilum aurantiacum	13492964	17182218	224968111	17182279	224982192
Cyrtochilum aureum	13492966	223371580	224967565	-	224982744
Cyrtochilum caespitosum	13492965	223370851	224968114	-	224982194
Cyrtochilum cimiciferum	13492731	16596775	224967269	16596779	224981640
Cyrtochilum cordatum	223371251	223370763	224967562	-	224981838
Cyrtochilum cumandae	223371268	223370779	224967613	-	224981864
Cyrtochilum edwardii	13492728	16596773	224967550	16596776	224981830
Cyrtochilum flexuosum	223371276	223370784	224967649	-	224981884
Cyrtochilum funis	223371579	223371064	224968957	-	224982740
Cyrtochilum gargantua	13492946	223371130	224969164	-	224982872
Cyrtochilum gracile	13492949	223371011	224968798	-	224982636
Cyrtochilum graminoides	13492967	17182228	224968150	17182284	224982218
Cyrtochilum halteratum	13492958	223370787	224967742	-	224981946
Cyrtochilum ioplocon	13492951	17182232	224967763	17182286	224981960
Cyrtochilum longipes	16596795	223370901	224968345	-	224982348
Cyrtochilum loxense	16596794	223370797	224967838	-	224982008
Cyrtochilum macranthum	126789406	126789178	224967898	-	224982048
Cyrtochilum meirax	17182117	17182234	224968609	17182287	224982512
Cyrtochilum murinum	223371487	223370971	224968654	-	224982540
Cyrtochilum myanthum	13492940	223370990	224968732	223370533	224982592
Cyrtochilum ornatum	223371532	223371015	224968810	-	224982644
Cyrtochilum pardinum	13492961	17182226	224967553	17182283	224981832
Cyrtochilum ramosissimum	13492956	223370746	224967445	-	224981756
Cyrtochilum revolutum	13492962	223370739	224967382	-	224981714
Cyrtochilum rhodoneurum	126789404	126789179	224967700	16596778	224981920
Cyrtochilum serratum	223371218	45758377	224967646	-	224996981
Cyrtochilum tricostatum	223371507	17182230	224967535	17182285	224981820
Cyrtochilum trifurcatum	223371266	223370777	224967607	-	224981860
Cyrtochilum trilingue	223371682	223371174	224969299	-	224982958
Cyrtochilum viminale	223371536	223371019	224968822	-	224982652
Cyrtopodium aliciae	215253654	13603713	215253674	-	-

Cyrtopodium andersonii	22087057	126789236	-	-	564291862
Cyrtopodium flavum	-	564291798	-	-	564292248
Cyrtopodium longibulbosum	-	564291774	-	-	564292110
Cyrtopodium punctatum	8699164	13603713	-	9789444	KY988621
Cyrtopodium saintlegerianum	KY988620	58200490	-	-	KY988622
Daiotyla albicans	58042634	58200490	-	58042548	161597650
Dichaea acroblephara	161597395	161597461	-	161597592	161597652
Dichaea ancoraelabia	161597399	161597465	-	161597594	161597654
Dichaea caveroi	161597401	126789217	-	161597595	161597658
Dichaea cryptarrhena	161597406	161597479	-	161597600	161597668
Dichaea dammeriana	161597408	161597483	-	161597602	161597672
Dichaea ecuadorensis	161597409	161597485	-	161597603	161597674
Dichaea eligulata	161597411	161597489	-	161597604	161597676
Dichaea elliptica	161597412	161597491	-	161597605	161597678
Dichaea fragrantissima	161597414	161597493	-	161597606	161597680
Dichaea glauca	161597416	161597497	-	161597607	161597684
Dichaea globosa	161597418	161597501	-	161597609	161597688
Dichaea hystricina	161597420	161597505	-	161597611	161597692
Dichaea lankesteri	161597423	161597511	-	161597614	161597698
Dichaea longa	161597425	161597515	-	161597615	161597702
Dichaea morrisii	161597427	161597519	167890798	161597616	161597706
Dichaea muyuyacensis	161597428	161597523	-	161597618	161597709
Dichaea neglecta	161597430	13603529	-	161597619	161597712
Dichaea obovatipetala	161597431	161597528	-	161597620	161597714
Dichaea oxyglossa	161597433	161597532	-	161597622	161597718
Dichaea panamensis	161597434	161597534	-	161597623	161597720
Dichaea pendula	161597437	161597540	-	161597625	161597726
Dichaea poicillantha	161597438	161597542	-	161597626	161597728
Dichaea potamophila	161597441	161597548	-	161597629	161597734
Dichaea riopalenquensis	161597444	161597552	-	161597631	161597736
Dichaea rubroviolacea	161597445	161597554	-	161597632	161597738
Dichaea sarapiquiensis	161597446	161597556	-	161597633	161597740

Dichaea squarrosa	161597453	161597570	-	161597640	161597754
Dichaea trichocarpa	161597455	161597574	-	161597642	161597758
Dichaea trulla	161597456	161597576	-	161597643	161597760
Dichaea tuerckheimii	161597459	161597582	-	161597646	161597765
Dichaea viridula	161597460	161597584	-	161597647	161597767
Dipodium paludosum	KY988619	126789234	-	-	KY988623
Dressleria dilecta	8699163	126789238	-	9789443	187372619
Dressleria fragrans	-	564291734	-	-	564291858
Dressleria helleri	-	564291732	-	-	564291856
Echinorhyncha litensis	58042657	58200536	-	58042569	-
Eloyella thienii	84782511	84782579	224968675	84657368	224982554
Embreea herrenhusana	-	564291716	-	-	564291832
Embreea rodigasiana	8699110	13603605	-	9789390	564291830
Epidendrum nocturnum	12330827	745790796	-	256682035	-
Eriopsis biloba	78095313	13603707	224968570	-	187372643
Eriopsis rutidobulbon	8699161	37222993	-	9789441	-
Erycina crista galli	223371470	167890000	220684420	13492790	224997632
Erycina echinata	13492708	15004414	224967466	13492787	224981768
Erycina glossomystax	223371607	223371094	224969053	223370545	224982802
Erycina hyalinobulbon	13492707	15004412	224967355	13492786	187372645
Erycina pumilio	13492710	15004418	224967724	13492789	224981936
Erycina pusilla	126789394	126789195	224967340	13492788	224981686
Erycina zamorensis	223371483	223370969	224968642	-	224982532
Eulophia aculeata	570759290	659898101	-	-	-
Eulophia acutilabra	570759295	659898127	-	-	-
Eulophia alta	215253655	-	215253675	-	-
Eulophia angolensis	570759273	659898099	-	-	KY988624
Eulophia calanthoides	570759237	659898131	-	-	-
Eulophia chlorantha	570759280	659898149	-	-	-
Eulophia coeloglossa	570759302	659898095	-	-	-
Eulophia cucullata	570759287	659898130	-	-	-
Eulophia ensata	570759298	659898106	-	-	-

Eulophia flava	387234530	353442294	-	-	-
Eulophia flavopurpurea	570759271	659898093	-	-	-
Eulophia foliosa	570759261	659898125	-	-	-
Eulophia graminea	21427048	223371180	224969317	-	224982970
Eulophia guineensis	570759301	13603715	-	9789445	187372647
Eulophia hereroensis	570759281	659898090	-	-	-
Eulophia hians	570759233	659898084	-	-	-
Eulophia horsfallii	570759263	659898133	-	-	-
Eulophia leontoglossa	570759282	659898157	-	-	-
Eulophia livingstoniana	570759284	659898081	-	-	-
Eulophia longisepala	570759259	659898110	-	-	-
Eulophia macowanii	570759245	659898145	-	-	-
Eulophia mechowii	570759255	659898126	-	-	-
Eulophia meleagris	570759277	659898107	-	-	-
Eulophia ovalis	570759246	659898088	-	-	-
Eulophia parvilabris	570759268	659898155	-	-	-
Eulophia petersii	570759238	126789230	-	-	564291868
Eulophia ruwenzoriensis	215253657	659898096	-	-	-
Eulophia schweinfurthii	570759265	659898158	-	-	-
Eulophia tabularis	570759248	659898116	-	-	-
Eulophia tenella	570759241	659898144	-	-	-
Eulophia tuberculata	570759286	659898100	-	-	-
Euryblema anatonum	58042650	58200522	-	58042562	-
Euryblema andreae	58042665	126789210	-	58042577	-
Fernandezia breviconnata	223371254	223370766	224967571	-	224981844
Fernandezia crystallina	223371611	223371098	224969065	223370546	224997946
Fernandezia cuencae	84782522	223371100	224969071	-	564292112
Fernandezia ecuadorensis	223371656	223371148	224969221	223370560	224982908
Fernandezia ionanthera	223371257	13603669	224967580	9789422	224981848
Fernandezia sanguinea	223371547	223371030	224968855	-	224982674
Fernandezia tica	223371474	223370965	224968297	223370527	224982316
Galeandra baueri	215253636	-	215253658	-	-

Galeandra beyrichii	215253649	-	215253687	-	-
Galeandra blanchetii	215253638	-	215253659	-	-
Galeandra devoniana	215253640	37222994	215253661	-	564291864
Galeandra greenwoodiana	215253642	-	215253663	-	-
Galeandra junceaoides	215253643	-	215253664	-	-
Galeandra magnicolumna	215253644	-	215253665	-	-
Galeandra minax	215253645	-	215253666	-	-
Galeandra paraguayensis	215253651	-	215253671	-	-
Galeandra santarena	215253641	-	215253662	-	-
Galeandra stangeana	215253646	-	215253667	-	-
Galeandra styllomisantha	215253652	-	215253672	-	-
Galeottia burkei	58042704	58200630	-	-	564292004
Galeottia ciliata	58042706	58200634	-	-	564292006
Galeottia colombiana	58042703	58200628	-	-	564291998
Galeottia fimbriata	58042709	58200640	-	58042613	-
Galeottia grandiflora	58042710	58200642	-	58042614	-
Geodorum densiflorum	21427047	353442306	-	-	-
Geodorum recurvum	675912725	675912487	-	675912354	-
Gomesa barbata	223371452	223370945	224968507	-	224982446
Gomesa chrysostoma	223371429	223370920	224968432	-	224982398
Gomesa ciliata	223371238	223370751	224967475	-	224981776
Gomesa cogniauxiana	223371430	223370921	224968435	-	224982400
Gomesa concolor	223371344	223370837	224968060	-	224982156
Gomesa crispa	223371436	223370927	224968453	-	224982412
Gomesa dasytyle	13492722	15004442	224967517	13492801	224996901
Gomesa eleutherosepala	223371224	223370738	224967367	223370502	224981704
Gomesa flexuosa	13492723	15004444	166244770	13492802	224981742
Gomesa forbesii	223371450	223370943	224968501	-	224982442
Gomesa gardneri	223371691	223371184	224969329	-	224982978
Gomesa glaziovii	223371454	223370947	224968513	-	224982450
Gomesa gomezoides	-	15004445	224967499	13492803	224996887
Gomesa hookeri	223371448	223370941	224968495	-	224982438

Gomesa imperatorismaximiliani	223371228	223370741	224967400	-	224981726
Gomesa lietzei	223371646	223371138	224969191	223370558	224982890
Gomesa longipes	223371407	223370903	224968351	-	224982352
Gomesa macronyx	223371220	223370734	224967328	-	224981678
Gomesa macropetala	223371445	223370936	224968480	-	224982428
Gomesa planifolia	126789402	126789169	224967325	13492804	224981676
Gomesa praetexta	223371433	223370924	224968444	-	224982406
Gomesa pubes	223371211	225580068	224967263	-	224981636
Gomesa radicans	223371645	223371137	224969188	223370557	224982888
Gomesa recurva	223371456	223370950	224968522	-	224982456
Gomesa sarcodes	223371443	223370934	224968474	-	224982424
Gomesa sessilis	223371455	223370948	224968516	-	224982452
Gomesa spiloptera	223371449	223370942	224968498	-	224982440
Gomesa varicosa	223371453	223370946	224968510	-	224982448
Gomesa venusta	223371379	223370871	224968207	-	224982256
Gomesa viperina	223371368	223370862	224968171	-	224982232
Gomesa warmingii	223371247	223370760	224967544	-	224981826
Gomesa welteri	223371437	223370928	224968456	-	224982414
Gomesa widgrenii	223371679	223371171	224969290	-	224982954
Gongora amparoana	8699137	13603659	-	9789417	564291938
Gongora armeniaca	8699138	13603661	-	9789418	564291872
Gongora escobariana	8699135	13603655	-	9789415	564291898
Gongora gratulabunda	8699134	13603653	-	9789414	-
Gongora ilense	8699136	13603657	-	9789416	564292222
Gongora portentosa	8699141	13603667	-	9789421	564291886
Gongora sphaerica	8699140	13603665	-	9789420	564291866
Gongora tridentata	8699139	13603663	-	9789419	564291950
Grammatophyllum measuresianum	-	223370967	-	-	-
Grammatophyllum scriptum	-	22087022	-	-	-
Grammatophyllum speciosum	22087055	13603717	-	22087023	-
Grandiphyllum auricula	223371684	223371176	220684492	223370529	224982962
Grandiphyllum divaricatum	223371476	223370967	224968615	223370528	224982516

Grandiphyllum hians	223371223	223370737	224967352	-	224981694
Graphorkis lurida	-	37222996	-	-	-
Grobya amherstiae	215253656	-	-	-	-
Grobya galeata	22087054	22087022	-	-	-
Heterotaxis brasiliensis	78095094	78094412	-	-	-
Heterotaxis equitans	758294107	758293985	-	-	564292100
Heterotaxis maleolens	78094917	167889651	-	-	564292176
Heterotaxis santanae	115394627	78094912	-	-	564292178
Heterotaxis sessilis	78095349	78094801	-	-	564292114
Heterotaxis valenzuelana	115394630	78094425	-	-	564292224
Heterotaxis violaceopunctata	58042727	13603553	-	58042628	161597769
Hintonella mexicana	223371468	223370961	224967988	223370525	224982108
Hofmeisterella eumicroscopica	13492681	15004360	224967436	13492760	224981750
Horichia dressleri	8699114	13603613	-	9789394	564291884
Houlletia sanderi	8699123	13603631	-	9789403	564291956
Houlletia tigrina	8699122	13603629	-	9789402	564291954
Houlletia wallisii	8699121	13603627	-	9789401	-
Huntleya gustavi	58042694	58200610	-	58042606	-
Huntleya wallisii	58042692	161597586	-	58042604	161597771
Inti bicallosa	758294140	758293929	-	-	564292038
Inti chartacifolia	78094920	78094725	-	-	187372657
Ionopsis minutiflora	13492719	15004436	224967685	13492798	224981910
Ionopsis satyrioides	13492718	15004434	224967682	13492797	224981908
Ionopsis utricularioides	13492717	15004432	224967343	13492796	224981688
Ixyophora viridisepala	58042659	58200540	-	58042571	564292040
Kefersteinia excentrica	58042651	167889576	-	58042563	564292218
Kefersteinia guacamayoana	58042652	58200526	-	58042564	-
Kefersteinia maculosa	58042655	58200532	-	58042567	564292048
Kefersteinia microcharis	58042654	167889580	-	58042566	564291820
Kefersteinia trullata	58042653	58200528	-	58042565	564292050
Kegeliella atropilosa	745672343	126789205	-	745672405	564291888
Kegeliella kupperi	8699116	13603617	-	9789396	564291882

Koellensteinia boliviensis	58042721	58200664	-	9789359	-
Lacaena spectabilis	745672348	126789206	-	745672410	564291896
Leochilus carinatus	126789401	126789167	224967284	223370499	224981650
Leochilus labiatus	223371355	225580258	224968090	-	224982176
Leochilus leiboldi	223371280	223370786	224967694	223370524	224981916
Leochilus leochilinus	223371232	225580092	224967415	223370503	224981736
Leochilus oncidioides	223371231	223370744	224967412	-	224981734
Leochilus tricuspidatus	223371662	223371154	224969239	-	224982920
Lockhartia acuta	223371196	223370715	224967216	-	224981602
Lockhartia amoena	126789419	126789101	224967189	13492765	224981584
Lockhartia bennettii	223371192	223370711	224967204	223370540	224981594
Lockhartia chocoensis	223371189	167889604	224967195	-	224981588
Lockhartia grandibractea	223371194	223370713	224967210	-	224981598
Lockhartia hercodonta	223371188	167889606	224967192	-	224981586
Lockhartia micrantha	223371193	223370712	224967207	223370494	224981596
Lockhartia oerstedii	13492685	13603677	224967213	13492764	224981600
Lockhartia parthenoglossa	223371199	223370719	224967227	-	224981610
Lueddemannia pescatorei	8699128	13603641	-	9789408	564292180
Lycomormium fiskei	8699097	223370860	-	-	-
Macradenia brassavolae	223371241	225580104	224967502	-	224981796
Macradenia rubescens	223371366	223370860	224968162	223370517	224982226
Macradenia tridentata	223371426	223370917	224968423	-	224982392
Macroclinium aurorae	223371647	223371139	224969194	-	224982892
Macroclinium bicolor	13492721	223371093	224967299	13492800	224981660
Macroclinium dalstroemii	223371606	225580266	224969050	-	224982800
Macroclinium robustum	223371365	225580266	224968129	-	224982204
Mapinguari auyantepuiensis	78095270	78094555	-	-	564292068
Mapinguari longipetiolatus	78095168	78094472	-	-	564291814
Maxillaria acostae	78094973	78094328	-	-	564292144
Maxillaria aequiloba	78095212	78094509	-	-	-
Maxillaria angustissima	78094993	78094686	-	-	564292162
Maxillaria arachnitiflora	78095002	167889634	-	-	-

Maxillaria bolivarensis	78095280	78094563	-	-	-
Maxillaria brachybulbon	78095201	167889636	-	-	564292032
Maxillaria bradei	78095088	78094406	-	-	-
Maxillaria canarina	269930073	564291790	-	-	564292240
Maxillaria carolii	78095512	78094773	-	-	-
Maxillaria chionantha	78094947	78094310	-	-	564292148
Maxillaria crocea	78095042	78094359	-	-	-
Maxillaria dalessandroi	78095305	78094585	-	-	564292202
Maxillaria dillonii	78095373	564291780	-	-	564292196
Maxillaria ecuadorensis	78095198	78094496	-	-	564292160
Maxillaria elegantula	78094963	78094321	-	-	564292124
Maxillaria endresii	78094949	78094311	-	-	-
Maxillaria fractiflexa	78095013	78094342	-	-	-
Maxillaria gentryi	78095286	78094570	-	-	564292154
Maxillaria grandis	78095307	78094587	-	-	-
Maxillaria grayi	78095486	564291782	-	-	564292198
Maxillaria henchmannii	78095238	78094523	-	-	-
Maxillaria hennisiana	78094952	78094643	-	-	564292122
Maxillaria huebschii	78095509	78094770	-	-	-
Maxillaria jostii	78095031	78094355	-	-	-
Maxillaria lepidota	78095302	78094582	-	-	-
Maxillaria leucaimata	78095046	78094363	-	-	-
Maxillaria longipes	78095016	78094345	-	-	564292172
Maxillaria longipetala	78095506	78094879	-	-	564292026
Maxillaria longissima	78095208	78094504	-	-	564292168
Maxillaria lueri	78094942	78094306	-	-	564292132
Maxillaria luteograndiflora	78095131	78094447	-	-	-
Maxillaria meridensis	78094976	78094495	-	-	564292090
Maxillaria modesta	78095134	78094451	-	-	-
Maxillaria molitor	78095308	78094588	-	-	-
Maxillaria pachyacron	78094960	78094318	-	-	-
Maxillaria patens	78095200	78094497	-	-	-

Maxillaria pauciflora	78095032	78094356	-	-	-
Maxillaria platypetala	78095497	78094758	-	-	564292208
Maxillaria porrecta	78094924	78094301	-	-	564292128
Maxillaria pulla	78095320	78094597	-	-	564292092
Maxillaria ramonensis	78095038	78094857	-	-	-
Maxillaria reichenheimiana	78094992	78094672	-	-	-
Maxillaria ringens	78094986	78094833	-	-	-
Maxillaria rodrigueziana	78095000	167889666	-	-	-
Maxillaria rotundilabia	78095345	78094618	-	-	-
Maxillaria sanderiana	78095210	78094506	-	-	-
Maxillaria setigera	78095082	78094399	-	-	-
Maxillaria silvana	78095455	78094722	-	-	564292170
Maxillaria speciosa	78095014	78094343	-	-	-
Maxillaria splendens	607840125	607840037	-	-	564292216
Maxillaria striata	78095206	78094502	-	-	-
Maxillaria virguncula	78095443	78094708	-	-	564292094
Maxillariella alba	78095110	167889632	-	-	-
Maxillariella anceps	78095457	78094910	-	-	-
Maxillariella caespitifica	78094974	78094822	-	-	-
Maxillariella diuturna	78095469	78094914	-	-	-
Maxillariella elatior	115394612	78094522	-	-	564292044
Maxillariella guareimensis	78095504	78094765	-	-	-
Maxillariella lawrenceana	78095390	78094661	-	-	-
Maxillariella linearifolia	78095035	78094854	-	-	-
Maxillariella longibracteata	78095292	78094575	-	-	-
Maxillariella oreocharis	78094943	78094307	-	-	564292150
Maxillariella pardalina	78095004	78094333	-	-	-
Maxillariella procurrens	115394622	78094507	-	-	564292080
Maxillariella punctata	78095494	126789222	-	-	564292186
Maxillariella robusta	78095131	78094447	-	-	-
Maxillariella sanguinea	78095020	78094849	-	-	-
Maxillariella tenuifolia	78095221	78094512	-	-	-

Maxillariella variabilis	115394631	126789220	-	-	564292166
Miltonia candida	13492688	126789259	-	13492767	224981664
Miltonia flavescens	13492687	15004372	224967652	13492766	224981886
Miltonia phymatochila	223371243	223371136	224967514	223370556	224981804
Miltonia regnellii	126789410	13603679	224968546	9789427	224982472
Miltoniopsis bismarkii	17182101	17182212	224968684	17182275	224982560
Miltoniopsis phalaenopsis	13492938	17182210	224967850	17182274	224982016
Miltoniopsis roezlii	223371692	223371186	224969332	223370564	224998124
Miltoniopsis vexillaria	13492937	223370748	224967457	-	224981764
Miltoniopsis warscewiczii	13492727	15004451	224967278	13492806	224981646
Mormodes andreettae	862568769	126789104	-	-	-
Mormodes aromatica	756808981	37223003	-	-	-
Mormodes vinacea	-	126789225	-	-	-
Mormolyca dressleriana	758294097	758293875	-	-	-
Mormolyca gracilipes	758294100	758293833	-	-	-
Mormolyca hedwigiae	758294114	758293829	-	-	-
Mormolyca peruviana	78095337	78094610	-	-	564292106
Mormolyca polyphylla	758294168	758293885	-	-	564292182
Mormolyca richii	758294160	758293849	-	-	564292072
Mormolyca ringens	758294098	126789243	-	-	564292190
Mormolyca rufescens	115394626	607840043	-	-	-
Mormolyca schlimii	758294150	126789218	-	-	564292076
Mormolyca schweinfurthiana	78095173	78094475	-	-	-
Mormolyca suareziorum	758294122	78094728	-	-	-
Mormolyca tenuibulba	758294101	758293827	-	-	-
Neogardneria murrayana	58042714	58200650	-	58042617	564292008
Nitidobulbon cymbidioides	78094926	78094802	-	-	-
Nitidobulbon nasutum	78095108	78094424	-	-	564292042
Nitidobulbon proboscideum	78094918	758293861	-	-	564291810
Notylia albida	223371634	223371126	224969152	-	224982864
Notylia barkeri	13492716	15004430	224967272	13492795	224981642
Notylia buchtienii	223371364	225580264	224968126	-	224982202

Notylia ecuadorensis	223371498	223370982	224968708	223370532	224982576
Notylia incurva	343527126	323388393	-	-	-
Notylia pittieri	223371202	223370722	224967236	-	224981616
Oeceoclades bernetii	570759278	659898092	-	-	-
Oeceoclades maculata	570759258	384584652	-	-	564292242
Oeceoclades ugandae	570759240	659898123	-	-	-
Oliveriana brevilabia	223371342	126789175	224968054	223370514	224982152
Oliveriana ecuadorensis	223371425	-	224968420	-	224982390
Oncidium alexandrae	13492877	223370798	224967775	-	224981968
Oncidium altissimum	13492894	225580224	224967937	223370512	224982074
Oncidium andradeanum	223371619	223371108	224969095	-	224982828
Oncidium ariasii	223371592	223371079	224969008	-	224982772
Oncidium aristuliferum	13492932	223370858	224968153	-	224982220
Oncidium aspidorhinum	13492861	223370802	224967886	-	224982040
Oncidium astranthum	13492871	225580204	224967883	-	224982038
Oncidium aurarium	223371375	223370867	224968195	-	224982248
Oncidium baueri	13492895	223371038	224967949	-	224982082
Oncidium blandum	13492879	225580150	224967739	-	224981944
Oncidium boothianum	223371556	223371039	224967706	-	224982692
Oncidium bracteatum	13492914	225580136	224967706	-	224981924
Oncidium buchtienoides	126789426	126789188	224968156	-	224982222
Oncidium cariniferum	13492887	225580188	224967832	-	224982004
Oncidium chrysomorphum	13492888	17182242	224967844	17182291	224982012
Oncidium cinnamomeum	13492846	225580166	224967781	-	224981972
Oncidium cirrhosum	13492850	223371072	224967373	-	224981708
Oncidium constrictum	13492855	225580174	224967811	-	224981990
Oncidium crinitum	13492856	225580210	224967907	-	224982054
Oncidium cristatum	13492859	225580216	224967916	-	224982060
Oncidium crocidipterum	13492880	225580130	224967703	-	224981922
Oncidium cuculligerum	13492934	223370857	224968144	-	224982214
Oncidium dactyliferum	223371267	223370778	224967610	-	224981862
Oncidium echinops	223371391	223371096	224968258	-	224982290

Oncidium endocharis	13492907	223370759	224967541	-	224981824
Oncidium ensatum	13492921	225580234	224967952	-	224982084
Oncidium epidendroides	13492869	223370823	224967796	223370534	224981980
Oncidium fuscatum	13492900	126789117	224967391	-	224981720
Oncidium ghiesbreghtianum	126789424	126789182	224967427	13492813	224981744
Oncidium gloriosum	13492857	225580212	224967910	-	224982056
Oncidium gramineum	13492927	223371005	224967991	-	224982110
Oncidium hallii	13492867	223370710	224967198	-	224981954
Oncidium harryanum	126789422	126789189	224967358	13492819	187372666
Oncidium hastatum	13492904	223370808	224967904	-	224982052
Oncidium hastilabium	13492922	223370736	224967337	-	224981684
Oncidium heteranthum	13492917	223371041	224968888	-	224982696
Oncidium heterodactylum	223371537	223371020	224968825	-	224982654
Oncidium hyphaematicum	13492916	223370816	224967970	223370513	224982096
Oncidium imitans	223371203	223370723	224967239	-	224981618
Oncidium incurvum	13492915	223371131	224968588	-	224982498
Oncidium isthmi	13492896	223371047	224967940	-	224982076
Oncidium kegeljani	223371553	223371036	224968873	-	224982686
Oncidium laeve	13492884	225580082	224967346	-	224981690
Oncidium lancifolium	223371590	223371077	224969002	-	224982768
Oncidium lehmannianum	223371416	223370912	224968378	223370520	224982368
Oncidium leucochilum	13492736	15004469	224967313	13492815	224981668
Oncidium lindleyoides	13492843	223370750	224967469	-	224981770
Oncidium luteopurpureum	13492849	223370753	224967490	-	224981784
Oncidium maculatum	13492898	225580230	224967946	-	224982080
Oncidium manuelariasii	223371406	223370902	224968348	-	224982350
Oncidium mirandum	13492864	225580218	224967919	-	224982062
Oncidium multistellare	126789421	126789186	224967508	13492821	224981800
Oncidium nevadense	13492841	225580110	224967547	-	224981828
Oncidium nobile	13492875	225580176	224967814	-	224981992
Oncidium noezlianum	126789420	126789184	224967385	13492820	224981716
Oncidium oblongatum	13492912	225580220	224967925	-	224982066

Oncidium obryzatoides	223371421	167890002	224968399	-	224982382
Oncidium obryzatum	13492909	223371062	224968951	-	224982736
Oncidium oliganthum	13492890	225580072	224967281	-	224981648
Oncidium ornithorhynchum	13492737	13603689	224967661	13492816	224981894
Oncidium oxyceras	223371517	223371001	224968765	-	224982614
Oncidium panamense	223371666	223371158	224969251	-	224982928
Oncidium picturatissimum	223371574	223371059	224968942	-	224982730
Oncidium planilabre	13492910	225580208	224967448	-	224997163
Oncidium poikilostalix	13492743	15004483	224967364	13492822	224981702
Oncidium polycladium	223371652	167889688	224969209	-	224982900
Oncidium portilloides	13492862	225580252	224968048	-	224982148
Oncidium portmannii	13492874	223370995	224967769	-	224981964
Oncidium povedanum	13492863	225580274	224968192	-	224982246
Oncidium praestanoides	13492851	225580246	224968042	-	224982144
Oncidium reflexum	13492891	223370756	224967511	-	224981802
Oncidium reichenheimii	13492733	15004463	224967316	13492812	224981670
Oncidium retusum	13492913	223370809	224967293	-	224981656
Oncidium reversoides	13492848	225580186	224967829	-	224982002
Oncidium rhynchanthum	13492854	223370754	224967493	-	224981788
Oncidium schroederianum	13492886	223370958	224967871	-	224982030
Oncidium spectatissimum	13492872	225580184	224967826	-	224982000
Oncidium sphacelatum	13492735	15004467	224967664	13492814	224981896
Oncidium stenoglossum	13492885	225580084	224967349	-	224981692
Oncidium storkii	223371422	167890004	224968402	-	224982384
Oncidium tenuifolium	223371404	223370900	224968342	-	224982346
Oncidium tigroides	13492889	126789187	224968921	-	224982718
Oncidium tipuloides	223371328	223370822	224967997	-	224982114
Oncidium toachicum	13492918	225580100	224967484	-	224981782
Oncidium trinasutum	223371341	225580254	224968051	-	224982150
Oncidium tripudians	13492852	225580180	224967820	-	224981996
Oncidium unguiculatum	223371492	225580332	224968681	-	224982558
Oncidium unguiculoides	13492926	225580190	224967835	-	224982006

Oncidium velleum	13492866	225580182	224967823	-	224981998
Oncidium vulcanicum	13492836	225580148	224967736	-	224982130
Oncidium wallisii	13492853	225580088	224967379	-	224981712
Oncidium warszewiczii	223371237	225580098	224967463	-	224981772
Oncidium wyattianum	13492842	225580192	224967847	-	224982014
Ornithidium adendrobium	78095153	167889630	-	-	-
Ornithidium aggregatum	523586034	523586244	-	-	564292102
Ornithidium coccineum	115394608	78094814	-	-	564292192
Ornithidium conduplicatum	78094980	78094828	-	-	-
Ornithidium fulgens	78095164	167889647	-	-	564292134
Ornithidium giganteum	78095199	78094542	-	-	564292058
Ornithidium gualaquizense	93359519	93359537	-	-	-
Ornithidium mapiriense	78095510	78094771	-	-	-
Ornithidium multicaule	78095496	78094757	-	-	564292206
Ornithidium pittieri	78095378	78094905	-	-	-
Ornithidium repens	78095009	78094338	-	-	-
Ornithidium rigidum	78095055	78094372	-	-	-
Ornithidium serrulatum	78095021	78094850	-	-	564292184
Ornithidium sophronitis	78095249	78094534	-	-	564292054
Ornithocephalus bicornis	223371585	223371070	224968978	223370544	224982754
Ornithocephalus ciliatus	223371582	223371067	224968969	-	224997882
Ornithocephalus cochleariformis	223371651	223371143	224969206	-	224998040
Ornithocephalus dalstroemii	223371206	223371103	224969116	-	224997980
Ornithocephalus dressleri	223371615	223371104	224969083	223370547	224997958
Ornithocephalus escobarianus	13492680	15004358	224967245	13492759	224981622
Ornithocephalus inflexus	13492678	13603671	224967201	13492757	224981592
Ornithocephalus iridifolius	84782516	223370854	224968132	-	224982206
Ornithocephalus myrticola	84782518	223370855	224968135	-	224982208
Ornithocephalus polyodon	223371586	223371071	224968981	-	224982756
Ornithocephalus suarezii	223371581	223371066	224968966	223370543	224982746
Otoglossum candelabrum	223371631	223371121	224969137	223370553	224997994
Otoglossum chiriquense	13492732	15004461	224967376	13492811	224981710

Otoglossum globuliferum	126789412	126789170	224967233	17182290	224981614
Otoglossum harlingii	13492971	17182238	224968693	17182289	224982566
Otoglossum scansor	223371649	223371141	224969200	-	224982896
Otostylis lepida	58042726	58200674	-	-	564292002
Pabstia jugosa	58042716	58200654	-	58042619	-
Pabstia viridis	58042708	58200638	-	-	-
Paphinia cristata	169303232	745672375	-	745672411	187372672
Paphinia neudeckeri	8699127	13603639	-	9789407	564291946
Peristeria elata	745672341	13603581	-	-	-
Peristeria lindenii	8699099	13603583	-	-	-
Pescatoria cerina	58042669	167889692	-	58042581	564291972
Pescatoria coronaria	58042671	58200564	-	58042583	564291940
Pescatoria lamellosa	58042670	58200562	-	58042582	564291942
Pescatoria lawrenceana	126789430	126789212	-	58042578	564291990
Pescatoria lehmannii	13492754	126789213	-	13492833	564292188
Pescatoria pulvinaris	58042667	58200556	-	58042579	564291980
Phymatidium falcifolium	223371383	225580280	224968225	-	224982268
Pityphyllum huancabambae	78095301	78094896	-	-	564292082
Pityphyllum saragurense	93359510	93359528	-	-	564292232
Plectrophora alata	223371384	225580282	224968228	-	224982270
Plectrophora cultrifolia	223371516	223371000	224968762	-	224982612
Plectrophora triquetra	223371515	223370999	224968759	-	224982610
Polycycnis aurita	8699124	13603633	-	9789404	-
Polycycnis gratiosa	745672342	13603635	-	9789405	187372694
Polycycnis surinamensis	8699126	13603637	-	9789406	-
Polyotidium huebneri	223371479	225580316	224968624	-	224982522
Polystachya albescens	564733141	270484620	-	-	564733269
Polystachya calluniflora	298263948	270484657	-	-	564733293
Polystachya concreta	298263995	270484674	-	71912655	-
Polystachya cultriformis	298263953	270484716	-	-	564291816
Polystachya galeata	12330783	270484757	-	37540165	-
Polystachya longiscapa	71912779	270484804	-	71912654	-

Polystachya mauritiana	300836841	558760899	-	558761112	-
Polystachya melliodora	298263971	270484813	-	558761076	-
Polystachya modesta	298263972	270484816	-	71912656	187372696
Polystachya pubescens	297660158	270484879	-	-	-
Polystachya pyramidalis	564733134	564733230	-	-	564733299
Promenaea ovatiloba	58042718	37223014	-	58042621	-
Promenaea stapelioides	58042719	58200660	-	58042622	161597773
Promenaea xanthina	58042717	58200656	-	58042620	564291936
Psychopsis limminghei	223371681	225580318	224969296	-	224998098
Psychopsis papilio	13492753	13603699	224967697	13492832	224981918
Psychopsis sanderae	223371219	223370733	224967322	223370501	224981674
Pterostemma antioquiense	223371417	225580292	224968381	223370521	224982370
Pterostemma benzingii	223371633	223371123	224969143	-	224997998
Rhetinantha aciantha	78095235	78094815	-	-	-
Rhetinantha acuminata	78095347	78094620	-	-	564292158
Rhetinantha divaricata	78095317	78094594	-	-	-
Rhetinantha friedrichsthalii	78095084	78094862	-	-	-
Rhetinantha notylioglossa	78095053	78094370	-	-	564291906
Rhetinantha scorpoidea	78094997	78094844	-	-	-
Rhynchostele bictoniensis	13492700	126789171	224967290	13492779	224981654
Rhynchostele candidula	223371311	223370807	224967901	-	224982050
Rhynchostele cervantesii	223371293	225580158	224967760	-	224981958
Rhynchostele cordata	223371291	164513219	224967751	-	224981952
Rhynchostele galeottiana	223371309	223370804	224967892	-	224982044
Rhynchostele londesboroughiana	13492701	15004400	224967529	13492780	224981816
Rhynchostele maculata	223371295	223370791	224967778	-	224981970
Rhynchostele madrensis	223371310	223370805	224967895	-	224982046
Rhynchostele majalis	223371302	223370799	224967859	-	224982022
Rhynchostele pygmaea	223371290	223370789	224967748	-	224981950
Rhynchostele rossii	223371292	225580156	224967757	-	224981956
Rhynchostele stellata	223371289	223370788	224967745	-	224981948
Rhynchostele uroskinneri	223371308	223370803	224967889	-	224982042

Rodriguezia batemanii	223371512	223370996	224968750	223370535	224982604
Rodriguezia chasei	223371491	225580330	224968678	-	224982556
Rodriguezia delcastilloi	13492714	15004426	224968573	13492793	224982488
Rodriguezia lanceolata	13492713	15004424	224967334	13492792	224981682
Rodriguezia leeana	223371565	223371048	224968909	-	224982710
Rodriguezia lehmannii	223371576	223371061	224968948	-	224982734
Rodriguezia pulchra	223371497	223370981	224968705	-	224982574
Rodriguezia satipoana	13492715	15004428	224968576	13492794	224982490
Rossioglossum ampliatum	121763188	13603703	224967257	223370496	224981632
Rossioglossum insleayi	223371304	225580200	224967874	-	224982032
Rossioglossum krameri	126789416	126789193	224967397	-	224981724
Rossioglossum oerstedii	223371285	225580140	224967718	223370509	224981932
Rossioglossum schlieperianum	126789415	126789194	224967266	223370497	224981638
Sauvetrea chicana	93359518	564291796	-	-	564292236
Sauvetrea laevilabris	78095273	78094557	-	-	564292070
Schlimmia stevensonii	8699119	13603623	-	9789399	-
Schunkea vierlingii	223371361	225580262	224968108	-	224982190
Scuticaria hadwenii	8699080	13603545	-	9789360	564291944
Scuticaria salesiana	78095324	78094600	-	18175464	564292098
Sievekingia colombiana	8699108	13603601	-	9789388	-
Sievekingia herrenhusana	8699109	13603603	-	9789389	-
Solenidium lunatum	223371380	225580276	224968210	-	224982258
Solenidium portillae	223371493	223370977	224968690	223370531	224982564
Soterosanthus shepheardii	745672344	13603611	-	745672406	187372723
Stanhopea anfracta	8699106	13603597	-	9789386	564292226
Stanhopea annulata	8699100	13603585	-	9789380	187372727
Stanhopea cirrhata	8699120	13603625	-	9789400	564291924
Stanhopea ecornuta	8699101	13603587	-	9789381	564291928
Stanhopea jenischiana	223371658	223371150	224969227	-	224982912
Stanhopea pulla	8699107	13603599	-	9789387	564291926
Stanhopea saccata	8699103	13603591	167890836	9789383	-
Stanhopea tigrina	223371245	13603593	-	9789384	187372729

Stenia bismarckii	58042637	126789214	-	58042551	564291988
Stenia calceolaris	58042636	58200494	-	58042550	564291992
Stenia glatzii	58042638	58200498	-	58042552	-
Stenia pallida	58042639	58200500	-	58042553	-
Stenotyla lankesteriana	58042679	58200580	-	58042591	564291970
Stenotyla lendyana	58042680	58200582	-	58042592	564291966
Stenotyla picta	58042678	58200578	-	58042590	564291994
Systeloglossum acuminatum	13492699	15004396	224967712	13492778	224981928
Systeloglossum bennettii	223371235	223370749	224967460	-	224981766
Telipogon acicularis	84782528	84782594	-	84657374	224997277
Telipogon ampliflorus	84782541	223370891	224968306	-	224982322
Telipogon ariasii	84782543	84782606	224968309	84657380	224982324
Telipogon barbozae	84782529	84782596	224968408	84657375	224997510
Telipogon biolleyi	84782544	84782608	224968312	84657381	224982326
Telipogon bombiformis	84782545	223370887	224968291	223370519	224982312
Telipogon bullpenensis	84782531	84782598	224968411	84657376	224997512
Telipogon butcheri	84782546	84782610	224968285	84657382	224982308
Telipogon caulescens	84782547	223370938	224968273	84657383	224982300
Telipogon chiriquensis	84782549	223370885	224968279	-	224982304
Telipogon hystrix	84782532	84782600	224968633	84657377	224982526
Telipogon klotzscheanus	84782557	223370939	224968489	-	224982434
Telipogon maduroi	84782558	223370888	224968294	-	224982314
Telipogon medusae	84782559	223370886	224968288	-	224982310
Telipogon monteverdensis	84782533	84782602	224968414	84657378	224997514
Telipogon nervosus	84782561	323388403	224968174	84657385	224982234
Telipogon obovatus	223371624	223371114	224969113	223370551	224982840
Telipogon olmosii	84782562	223370895	224968321	-	224982334
Telipogon panamensis	84782563	84782618	224968282	84657386	224982306
Telipogon parvulus	13492684	13603675	224968327	13492763	224982338
Telipogon personatus	84782565	225580290	224968330	-	224982340
Telipogon pogonostalix	13492682	13603673	224967451	13492761	224981760
Telipogon pulcher	84782566	84782622	-	84657388	224982182

Telipogon smaragdinus	84782535	223371055	224968930	-	224982722
Telipogon vargasii	84782571	84782626	224968168	84657390	224982230
Telipogon venustus	223371204	223370724	224967242	-	224981620
Thysanoglossa jordanensis	126789390	126789197	-	-	-
Tolumnia arizajuliana	223371338	223370833	224968036	-	224982140
Tolumnia calochila	13492703	15004404	224967538	13492782	224981822
Tolumnia compressicaulis	223371336	223370831	224968030	-	224982136
Tolumnia gauntlettii	223371378	223370870	224968204	-	224982254
Tolumnia guianensis	223371314	223370811	224967931	-	224982070
Tolumnia guibertiana	223371457	223370951	224968525	-	224982458
Tolumnia gundlachii	223371661	223371153	224969236	-	224982918
Tolumnia haitiensis	223371408	223370904	224968354	-	224982354
Tolumnia hawkesiana	223371357	223370848	224968096	-	224982180
Tolumnia henekenii	13492706	15004410	224967688	13492785	224981912
Tolumnia lemoniana	223371636	223371128	224969158	-	224982868
Tolumnia prionochila	223371315	223370812	224967934	-	224982072
Tolumnia pulchella	223371348	223370841	224968072	-	224982164
Tolumnia quadriloba	223371501	223370985	224968717	-	224982582
Tolumnia sasseri	223371356	223370847	224968093	-	224982178
Tolumnia scandens	223371324	223370819	224967979	-	224982102
Tolumnia sylvestris	223371335	223370830	224968027	-	224982134
Tolumnia triquetra	223371351	223370844	224968078	-	224982168
Tolumnia tuerckheimii	13492705	15004408	224967691	13492784	224981914
Tolumnia urophylla	223371313	223370810	224967928	-	224982068
Tolumnia variegata	13492704	15004406	224968714	13492783	224982580
Trevoria escobariana	126789429	126789208	-	745672408	-
Trevoria zahlbruckneriana	8699117	13603619	-	9789397	564291852
Trichocentrum bicallosum	223371229	223370742	224967406	-	224981730
Trichocentrum cebolleta	223371690	223371183	224969326	13492823	224998118
Trichocentrum cepula	223371685	223371178	224969311	-	224998108
Trichocentrum cosymbephorum	223371494	223370978	224968696	-	224997704
Trichocentrum cymbiglossum	223371617	223371106	224969089	-	224982824

Trichocentrum jonesianum	13492745	15004487	224967421	13492824	224981740
Trichocentrum lacerum	223371667	223371159	224969254	223370561	224998072
Trichocentrum lanceanum	13492748	126789256	224967658	13492827	224981892
Trichocentrum lindenii	223371566	225580272	224968912	-	224982712
Trichocentrum longicalcaratum	223371545	223371028	224968849	-	224982670
Trichocentrum margalefii	223371675	223371167	224969278	-	224982946
Trichocentrum morenoi	223371462	223370956	224968558	-	224982478
Trichocentrum nanum	223371650	223371142	224969203	-	224982898
Trichocentrum oerstedii	223371371	223370865	224968183	-	224982240
Trichocentrum panduratum	223371230	223370743	224967409	-	224981732
Trichocentrum pfavii	13492746	13603691	224967670	13492825	224981900
Trichocentrum pulchrum	169303233	223370975	224968672	-	224982552
Trichocentrum pumilum	223371676	223371168	224969281	-	224982948
Trichocentrum stramineum	223371369	223370863	224968177	-	224997357
Trichocentrum tigrinum	223371213	186972406	224967275	223370498	187372734
Trichoceros antennifer	13492683	15004364	224967478	13492762	224981778
Trichoceros muralis	223371489	225580328	224968669	84657391	224982550
Trichopilia brevis	13492752	15004501	224967403	13492831	224981728
Trichopilia fragrans	223371589	223371074	224968990	-	224982762
Trichopilia laxa	223371246	223370758	224967526	-	224981814
Trichopilia leucoxantha	223371642	223371134	224969179	-	224982882
Trichopilia sanguinolenta	13492751	15004499	224968783	13492830	187372738
Trichopilia suavis	223371208	223370729	224967254	-	224981630
Trichopilia subulata	13492749	15004495	224967496	13492828	224981790
Trichopilia turialbae	223371279	167890042	224967673	-	224981902
Trigonidium egertonianum	399144501	13603547	-	9789361	564291916
Trigonidium insigne	78095205	78094501	-	-	564292212
Trigonidium obtusum	78095049	78094366	-	-	-
Trigonidium riopalenquense	78094953	78094312	-	-	-
Trigonidium turbinatum	78095122	78094438	-	-	564291914
Trizeuxis falcata	223371234	167889834	224967454	223370504	224981762
Vanda tessellata	71912898	353442674	576703379	293618067	-

Vargasiella peruviana	588295514	588295515	-	588295517	-
Vasqueziella boliviana	8699129	13603643	-	9789409	564291892
Vitekorchis excavata	13492935	223370771	224967586	223370552	224981852
Vitekorchis lucasiana	223371594	223371081	224969014	-	224982776
Warczewiczella discolor	58042676	58200574	-	58042588	564292028
Warczewiczella guianensis	58042673	58200568	-	58042585	-
Warczewiczella lipscombiae	58042675	58200566	-	58042584	-
Warczewiczella marginata	58042675	58200572	-	58042587	187372742
Warmingia eugenii	223371217	225580078	224967310	-	224981666
Warmingia zamorana	223371390	225580284	224968255	-	224982288
Warreopsis colorata	58042701	58200624	-	-	-
Warreopsis pardina	58042702	58200626	-	-	-
Zelenkoa onusta	13492726	126789168	224967388	13492805	224981718
Zygopetalum maculatum	58042715	18027086	-	58042618	653816614
Zygostates alleniana	126789391	126789198	224968138	84657393	224982210
Zygostates apiculata	223371640	223371132	224969170	223370554	224982876
Zygostates grandiflora	13492679	15004356	224967430	13492758	224981746
Zygostates lunata	84782578	223370856	224968141	-	224982212
Zygostates obliqua	84782509	223370859	224968159	-	224982224

Table S2

Scientific Name	ITS	*mat* K
Acianthera aphthosa	JQ306355	-
Acianthera asaroides	JQ306431	-
Acianthera barbacenensis	JQ306423	-
Acianthera binotii	JQ306361	-
Acianthera binotii	JQ306430	-
Acianthera bragae	JQ306362	-
Acianthera butcheri	KY084270	KY218749
Acianthera capanemae	JQ306367	-
Acianthera capillaris	JQ306424	-
Acianthera capillaris	JQ306425	-
Acianthera cogniauxiana	KR816545	KR816554
Acianthera compressiflora	JQ306436	-
Acianthera crinita	JQ306434	-
Acianthera crinita	JQ306435	-
Acianthera cryptantha	JQ306433	-
Acianthera decipiens	KR816546	KR816555
Acianthera enianthera	JQ306394	-
Acianthera fabiobarrosii	JQ306426	-
Acianthera fenestrata	AF262857	AF265468
Acianthera foetens	JQ306432	-
Acianthera fornograndensis	JQ306448	-
Acianthera fornograndensis	JQ306353	-
Acianthera freyi	JQ306494	-
Acianthera geminicaulina	KY084275	KY218756
Acianthera glanduligera	JQ306369	-
Acianthera gracilisepala	JQ306404	-
Acianthera hamata	KY084277	-
Acianthera hamosa	JQ306447	-
Acianthera hamosa	JQ306374	-

Acianthera hatschbachii	JQ306406	-
Acianthera heringeri	JQ306363	-
Acianthera jordanensis	JQ306378	-
Acianthera karlii	JQ306489	-
Acianthera klotzschiana	JQ306445	-
Acianthera lanceana	KR816548	KR816557
Acianthera limae	JQ306450	-
Acianthera lojae	KR816549	KR816558
Acianthera micrantha	JQ306373	-
Acianthera minima	JQ306382	-
Acianthera montana	JQ306437	-
Acianthera nemorosa	JQ306466	-
Acianthera ochreata	AF262858	AY008458
Acianthera ochreata	JQ306354	-
Acianthera ochreata	JQ306427	-
Acianthera odontotepala	KY081773	-
Acianthera oscitans	KY988806	KY988625
Acianthera panduripetala	JQ306371	-
Acianthera papillosa	JQ306501	-
Acianthera pavimentata	JQ306443	-
Acianthera pavimentata	JQ306444	-
Acianthera perdusenii	JQ306454	-
Acianthera platystachys	JQ306428	-
Acianthera prolifera	AF275688	AF275697
Acianthera pubescens	JQ306365	-
Acianthera pubescens	JQ306366	-
Acianthera ramosa	JQ306438	-
Acianthera recurva	JQ306375	-
Acianthera rodriguesii	JQ306446	-
Acianthera rubroviridis	KY081774	-
Acianthera saundersiana	EF079371	EF079330
Acianthera saundersiana	JQ306452	-

Acianthera saurocephala	AF262851	AF265469
Acianthera saurocephala	JQ306356	-
Acianthera serpentula	JQ306370	-
Acianthera serpentula	JQ306411	-
Acianthera sicaria	KR816550	KR816559
Acianthera sonderiana	JQ306377	-
Acianthera sonderiana	JQ306496	-
Acianthera spilantha	JQ306359	-
Acianthera spilantha	JQ306360	-
Acianthera strupifolia	JQ306440	-
Acianthera testifolia	KR816551	KR816560
Acianthera testifolia	KR816552	-
Acianthera translucida	JQ306368	-
Acianthera tricarinata	JQ306495	-
Acianthera wilsonii	KY081776	-
Anathallis adenochila	KC425725	-
Anathallis adenochila	JQ306490	-
Anathallis aristulata	JQ306338	-
Anathallis bolsanelloi	JQ306342	-
Anathallis burzlaffiana	KC425727	KC425857
Anathallis citrina	JQ306498	-
Anathallis depauperata	KC425735	KY988626
Anathallis funerea	KY988807	KY988627
Anathallis grayumii	KC425730	-
Anathallis grayumii	KC425731	KP012494
Anathallis heterophylla	JQ306339	-
Anathallis kautskyi	JQ306340	-
Anathallis lewisiae	KC425733	KC425858
Anathallis nanifolia	KC425736	-
Anathallis nectarifera	JQ306458	-
Anathallis obovata	JQ306497	-
Anathallis obovata	JQ306337	-

Anathallis ourobranquensis	JQ306459	-
Anathallis pabstii	KC425737	KC425859
Anathallis pachyphyta	KC425734	-
Anathallis peroupavae	KF747837	-
Anathallis petropolitana	JQ306491	-
Anathallis piratiningana	JQ306344	-
Anathallis pubipetala	JQ306460	-
Anathallis rabei	KC425738	KC425860
Anathallis radialis	JQ306345	-
Anathallis rudolfii	JQ306461	-
Anathallis welteri	JQ306341	-
Andinia longiserpens	KC425744	KP012521
Andinia pensilis	AF262826	AF265455
Andinia platysepala	JQ995331	KC425864
Andinia schizopogon	KC425740	KP012518
Arpophyllum giganteum	AF266742	AF265485
Barbosella australis	KY988808	KY988628
Barbosella cucullata	AF262815	AF265483
Brachionidium kirbyi	KY988809	KY988629
Dilomilis montana	AF260147	AY368404
Dilomilis montana	AF262915	AF263765
Diodonopsis erinacea	AF262788	EU214336
Draconanthes aberrans	KY988810	KY988630
Dracula amaliae	EF372351	-
Dracula andreettae	EF372357	-
Dracula andreettae	AF262765	-
Dracula astuta	EF372365	-
Dracula astuta	AF262762	-
Dracula bella	AF262760	-
Dracula chestertonii	AF262758	-
Dracula chimaera	EF372358	-
Dracula chimaera	AF262766	AF265444

Dracula cochliops	AF262767	-
Dracula cordobae	EF372355	-
Dracula diabola	EF372363	-
Dracula dodsonii	AF262759	-
Dracula erythrochaete	EF372364	-
Dracula erythrochaete	AF262763	EU214337
Dracula gorgona	EF372377	-
Dracula hirtzii	EF372382	-
Dracula inexperata	KY988811	KY988631
Dracula iricolor	EF372392	-
Dracula lemurella	EF372361	-
Dracula polyphemus	EF372368	-
Dracula radiosa	EF372373	-
Dracula rezekiana	EF372376	-
Dracula roezlii	EF372381	-
Dracula sodiroi	AF262764	-
Dracula vampira	EF372356	-
Dracula vampira	AF262761	-
Dracula wallisii	EF372375	-
Dresslerella elvallensis	KY988812	KY988632
Dresslerella elvallensis	AF262901	-
Dresslerella hirsutissima	AF262902	-
Dresslerella hispida	KY988813	KP012427
Dresslerella hispida	KY988814	KY988633
Dresslerella hispida	KY988815	KP012428
Dresslerella hispida	KY988816	KY988634
Dresslerella pertusa	KY988817	-
Dresslerella pertusa	AF262903	-
Dresslerella pilosissima	KY988818	KP012446
Dresslerella sp.	KY988819	KY988635
Dryadella albicans	KC425742	KC425863
Dryadella aviceps	JQ306381	-

Dryadella edwallii	AF262824	AF265454
Dryadella fuschi	KY988820	KY988636
Dryadella guatemalensis	KC425743	-
Dryadella hirtzii	EF079367	EF079327
Dryadella kautskyi	JQ306380	-
Dryadella simula	AF262825	AF265453
Dryadella susanae	KQ306486	-
Echinosepala aspasicensis	AF262905	AF302645
Echinosepala pan	KP012471	KP012429
Echinosepala sempergemmata	KP012473	KY988637
Frondaria caulescens	AF262914	AF265471
Kraenzlinella anfracta	KY084291	KY218777
Kraenzlinella erinacea	KY084293	KY218778
Kraenzlinella sp	KY084289	KY218773
Lankesteriana barbulata	KF747834	-
Lankesteriana barbulata	KC425726	-
Lankesteriana barbulata	JQ306457	-
Lankesteriana casualis	KY988821	KY988638
Lankesteriana cuspidata	KF747835	-
Lankesteriana duplooyi	KF747836	-
Lankesteriana fractiflexa	KC425729	-
Lepanthes ankistra	KY988822	KY988639
Lepanthes atrata	KY988823	KY988640
Lepanthes blephariglossa	KY988824	KY988641
Lepanthes blephariglossa	KY988825	KY988642
Lepanthes blepharistes	KY988826	KY988643
Lepanthes blepharistes	KY988827	KY988644
Lepanthes blepharistes	KY988828	KY988645
Lepanthes blepharistes	KY988829	KY988646
Lepanthes bradei	KY988830	KY988647
Lepanthes brunnescens	KY988831	KY988648
Lepanthes calliope	KY988832	KY988649

Lepanthes calodyction	KY988833	KY988650
Lepanthes candida	KY988834	KY988651
Lepanthes caprimulgus	KY988835	KY988652
Lepanthes cascajalensis	KY988836	KY988653
Lepanthes cloesii	KY988837	KY988654
Lepanthes confusa	KY988838	KY988655
Lepanthes cribii	KY988839	KY988656
Lepanthes cuspidata	KY988840	KY988657
Lepanthes decipiens	KY988841	KY988658
Lepanthes demissa	KY988842	KY988659
Lepanthes dikoensis	KY988843	KY988660
Lepanthes disticha	KY988845	KY988661
Lepanthes dolabriformis	KY988846	KY988662
Lepanthes dolabriformis	KY988847	KY988663
Lepanthes droseroides	KY988848	KY988664
Lepanthes dubbeldamii	KY988849	KY988665
Lepanthes elata	KY988850	KY988666
Lepanthes elata	KY988851	KY988667
Lepanthes elata	KY988852	KY988668
Lepanthes elegans	KY988853	KY988669
Lepanthes eximia	KY988854	KY988670
Lepanthes ferrelliae	KY988855	KY988671
Lepanthes gargantua	KY988856	KY988672
Lepanthes glicensteinii	KY988857	KY988673
Lepanthes gracillima	KY988858	KY988674
Lepanthes gustavoromeroi	KY988859	KY988675
Lepanthes hermansii	KY988860	KY988676
Lepanthes horichii	KY988861	KY988677
Lepanthes kleinii	KY988862	KY988678
Lepanthes latisepala	KY988863	KY988679
Lepanthes latisepala	KY988864	KY988680
Lepanthes lindleyana	KY988865	KY988681

Lepanthes lindleyana	KY988866	KY988682
Lepanthes machogaffensis	KY988867	KY988683
Lepanthes maduroi	KY988868	KY988684
Lepanthes martinae	KY988869	KY988685
Lepanthes matamorosii	KY988870	KY988686
Lepanthes mentosa	KY988871	KY988687
Lepanthes monteverdensis	KY988872	KY988688
Lepanthes montisnarae	KY988873	KY988689
Lepanthes myiophora	KY988874	KY988690
Lepanthes mystax	KY988875	KY988691
Lepanthes nycteris	KY988876	KY988692
Lepanthes olmosii	KY988877	KY988693
Lepanthes pulcherrima	KY988878	KY988694
Lepanthes queveriensis	KY988879	KY988695
Lepanthes rafaeliana	KY988880	KY988696
Lepanthes regularis	KY988881	KY988697
Lepanthes ribes	KY988882	KY988698
Lepanthes saltatrix	KY988883	KY988699
Lepanthes sandiorum	KY988884	KY988700
Lepanthes siboei	KY988885	KY988701
Lepanthes sijmii	KY988886	KY988702
Lepanthes spadariae	KY988887	KY988703
Lepanthes stenorrhyncha	KY988888	KY988704
Lepanthes terborchii	KY988889	KY988705
Lepanthes tristis	KY988890	KY988706
Lepanthes turialvae	KY988891	KY988707
Lepanthes variabilis	KY988892	KY988708
Lepanthes velosa	KY988893	KY988709
Lepanthes wendlandii	KY988894	KY988710
Lepanthes whittenii	KY988895	KY988711
Lepanthes williamsii	KY988896	KY988712
Lepanthopsis astrophora	AF262893	AF265487

Lepanthopsis floripecten	KY988897	KY988713
Lepanthopsis floripecten	KY988898	-
Masdevallia amaluzae	AF262799	-
Masdevallia ampullacea	AF262772	-
Masdevallia aphanes	AF262802	-
Masdevallia bicornis	AF262792	-
Masdevallia caesia	AF262786	-
Masdevallia caloptera	AF262773	-
Masdevallia calura	KY988899	KY988715
Masdevallia calura	KY988900	KY988716
Masdevallia caudivolvula	AF262770	-
Masdevallia chaparensis	AF262797	-
Masdevallia citrinella	AF262774	-
Masdevallia coccinea	AF262789	-
Masdevallia collina	AF262784	-
Masdevallia coriacea	AF262781	-
Masdevallia decumana	AF262795	-
Masdevallia eburnea	KY988901	KY988717
Masdevallia exquisita	KF479479	-
Masdevallia flaveola	KY988902	KY988718
Masdevallia floribunda	AF262776	AY368416
Masdevallia fulvescens	KY988903	KY988719
Masdevallia heteroptera	AF262800	-
Masdevallia hieroglyphica	AF262798	-
Masdevallia infracta	AF262785	-
Masdevallia infracta	JQ306379	-
Masdevallia kyphonantha	AF262780	-
Masdevallia lata	KY988905	KY988721
Masdevallia limax	AF262796	-
Masdevallia mentosa	AF262777	-
Masdevallia molossus	KY988906	KY988722
Masdevallia nidifica	AF262787	-

Masdevallia nidifica	-	JQ771566
Masdevallia ophioglossa	AF262790	-
Masdevallia oreas	AF262779	-
Masdevallia picturata	AF262775	-
Masdevallia pinocchio	AF262778	AF265445
Masdevallia racemosa	AF262771	-
Masdevallia reichenbachiana	AF262783	-
Masdevallia rubeola	AF262791	-
Masdevallia saltatrix	AF262793	-
Masdevallia scandens	KF479476	-
Masdevallia smallmanniana	KY988907	KY988723
Masdevallia sp.	KY988908	KY988724
Masdevallia striatella	KY988909	KY988725
Masdevallia titan	AF262803	-
Masdevallia uniflora	AF262769	AF265446
Masdevallia venezuelana	AF262782	-
Masdevallia ximenae	AF262794	-
Masdevallia yungasensis	KF478478	-
Masdevallia zahlbruckneri	KY988910	KY988726
Masdevallia zahlbruckneri	KY988911	KY988727
Muscarella aristata	KC425842	-
Muscarella cabellensis	KF747794	KP012396
Muscarella catoxys	KY988912	KY988728
Muscarella claviculata	KC425777	-
Muscarella fimbriata	KC425785	-
Muscarella furcatipetala	KY988913	KY988729
Muscarella hastata	KF747773	KR816553
Muscarella helenae	KC425847	-
Muscarella herpestes	KC425801	-
Muscarella llamachoi	KC425848	-
Muscarella longilabris	KC425849	-
Muscarella macroblepharis	KC425805	-

Muscarella marginata	KC425806	-
Muscarella megalops	KC425807	-
Muscarella mucronata	KC425850	-
Muscarella obliquipetala	KC425851	-
Muscarella quinqueseta	KC425817	-
Muscarella schudelii	KY988914	KY988730
Muscarella segregafolia	KC425822	-
Muscarella semperflorens	KY988915	KY988731
Muscarella sp.	KF747774	KP012419
Muscarella sp.	KY988916	KY988732
Muscarella strumosa	KC425831	-
Myoxanthus colothrix	KY988917	KP012438
Myoxanthus exasperatus	KY988918	KP012439
Myoxanthus hirsuticaulis	KY988919	KP012442
Myoxanthus lonchophyllus	AF262884	-
Myoxanthus octomeriae	KY988920	KP012444
Myoxanthus ovatipetalus	JQ306351	-
Myoxanthus punctatus	AF262885	AF265479
Myoxanthus ruschii	JQ306352	-
Myoxanthus scandens	KY988921	KP012443
Myoxanthus seidelii	JQ306350	-
Myoxanthus serripetalus	AF262883	-
Myoxanthus sp.	KY988922	KP012440
Myoxanthus sp.	KY988923	KP012441
Neocogniauxia hexaptera	AF260148	AF263766
Neocogniauxia hexaptera	AY429385	-
Neocogniauxia monophylla	AY008474	-
Octomeria costaricensis	KY988924	KY988733
Octomeria gracilis	AF262911	AF265484
Octomeria valerioi	KY988925	KY988734
Pabstiella alligatorifera	JQ306467	-
Pabstiella arcuata	JQ306477	-

Pabstiella aryter	JF934816	JF934876
Pabstiella aurantiaca	JQ306357	-
Pabstiella biriricensis	JQ306463	-
Pabstiella birricensis	JQ306409	-
Pabstiella bowmannii	JQ306483	-
Pabstiella carinifera	JQ306397	-
Pabstiella castellensis	JQ306484	-
Pabstiella conceicionensis	JQ306470	-
Pabstiella conceicionensis	JQ306499	-
Pabstiella ephemera	JQ995332	-
Pabstiella fasciata	JQ306473	-
Pabstiella hians	JQ306478	-
Pabstiella hypnicola	JQ995333	KY988735
Pabstiella lingua	JQ306387	-
Pabstiella lueriana	JQ306474	-
Pabstiella lueriana	JQ306475	-
Pabstiella mentosa	AF262864	-
Pabstiella mirabilis	AF262830	-
Pabstiella pellifeloidis	JQ306479	-
Pabstiella pellifeloidis	JQ306476	-
Pabstiella pterophora	JQ306396	-
Pabstiella pterophora	JQ306396	-
Pabstiella punctatifolia	JQ306346	-
Pabstiella punctatifolia	JQ306395	-
Pabstiella punctatifolia	JQ306346	-
Pabstiella rhombilabia	JQ306488	-
Pabstiella rubrolineata	JQ306393	-
Pabstiella seriata	JQ306465	-
Pabstiella seriata	JQ306464	-
Pabstiella seriata	JQ306465	-
Pabstiella sordida	JQ306399	-
Pabstiella sp.	JQ306462	-

Pabstiella teschiana	JQ306480	-
Pabstiella transparens	KF747838	-
Pabstiella tricolor	JQ306429	-
Pabstiella trifida	JQ306390	-
Pabstiella tripterantha	AF275694	-
Pabstiella tripterantha	JF934815	-
Pabstiella uniflora	JQ306469	-
Pabstiella villosisepala	JQ306471	-
Pabstiella villosisepala	JQ306472	-
Pabstiella wacketii	JQ995334	KC425856
Pabstiella wawraeana	JQ306407	-
Phloeophila nummularia	KF747839	KP012380
Phloeophila nummularia	KC425841	-
Phloeophila pelecaniceps	AF262810	AF265450
Phloeophila peperomioides	AF275690	AF291103
Phloeophila peperomioides	KC425745	-
Phloeophila pleurothallopsis	KC425746	KP012495
Phloeophila pleurothallopsis	KC425747	KP012496
Phloeophila pleurothallopsis	AF262811	AF265451
Platystele acicularis	KF747778	-
Platystele aff. reflexa	KC425756	-
Platystele beatricis	KC425749	KP012499
Platystele consobrina	KC425751	-
Platystele gyroglossa	KC425752	-
Platystele hirtzii	KF747780	-
Platystele jungermannioides	KY988926	KY988736
Platystele lancilabris	KP012493	KP012386
Platystele microtatantha	KF747781	-
Platystele minimiflora	KF747782	KP012387
Platystele misasiana	KF747783	KP012388
Platystele propinqua	KF747785	KP012390
Platystele resimula	KY988927	KY988737

Platystele schmidtchenii	KF747786	-
Platystele sp. nov.	KY988928	-
Platystele stenostachya	KF747787	-
Platystele stenostachya	KC425759	KP012501
Platystele tica	KP012458	KP012391
Pleurothallis adventurae	KY988929	KY988738
Pleurothallis allenii	AF262844	-
Pleurothallis anceps	KY988930	KY988739
Pleurothallis anthrax	KY988931	KY988740
Pleurothallis arietina	KY988932	KY988741
Pleurothallis aurantiaca	JQ306357	-
Pleurothallis aurita	KY988933	KY988742
Pleurothallis bivalvis	JQ306400	-
Pleurothallis bogarinii	KY988934	KY988743
Pleurothallis cardiantha	AF262832	-
Pleurothallis cardiothallis	AF262917	-
Pleurothallis cardiothallis	KY988935	-
Pleurothallis cardiothallis	-	KY988744
Pleurothallis cypelligera	KY988937	KY988745
Pleurothallis dentipetala	KY988938	KY988746
Pleurothallis discoidea	JQ306401	-
Pleurothallis divaricans	KY988939	KY988747
Pleurothallis dorotheae	KY988940	KY988748
Pleurothallis eumecocaulon	KY988941	KY988749
Pleurothallis fantastica	KC425761	-
Pleurothallis gratiosa	KY988942	KY988750
Pleurothallis helleri	KY988943	KY988751
Pleurothallis hemirhoda	AF262874	-
Pleurothallis inornata	KY988944	KY988752
Pleurothallis loranthophylla	AF262837	-
Pleurothallis matudana	KY988945	-
Pleurothallis miranda	AF262875	-

Pleurothallis niveoglobula	AF262839	-
Pleurothallis pruinosa	KY988946	KY988753
Pleurothallis quadrifida	JF934824	-
Pleurothallis quadrifida	JF934825	-
Pleurothallis racemiflora	AY008477	AY396076
Pleurothallis renniana	KY988947	KY988754
Pleurothallis restrepioides	JF934795	JF934856
Pleurothallis rowleei	KY988948	KY988755
Pleurothallis rowleei	KY988949	KY988756
Pleurothallis ruscaria	KY988950	KY988757
Pleurothallis ruscifolia	JF934814	JF934873
Pleurothallis scaphipetala	KY988951	KY988758
Pleurothallis scoparum	KY988952	KY988759
Pleurothallis silvaepacis	JQ995336	KY988760
Pleurothallis silverstonei	KY988953	KY988761
Pleurothallis sp.	KY988954	KY988762
Pleurothallis teaguei	AF275695	-
Pleurothallis tonduzii	KY988955	-
Pleurothallis tonduzii	KY988956	KY988763
Pleurothallis truncata	AF262834	-
Pleurothallis viduata	AF262838	-
Pleurothallis volcanica	KY988957	KY988764
Pleurothallopsis microptera	KY988958	KY988765
Pleurothallopsis nemorosa	AF291099	AF291104
Pleurothallopsis reichenbachiana	KY988959	KY988766
Pleurothallopsis striata	KY988960	KY988767
Pleurothallopsis tubulosa	KY988961	KY988768
Porroglossum amethystinum	AF262804	AF265448
Porroglossum apoloae	KY988962	-
Porroglossum oversteegenianum	KY988963	-
Porroglossum rodrigoi	AF262805	-
Porroglossum sijmii	KY988964	-

Porroglossum uxorium	AF262806	-
Restrepia antennifera	AF262906	-
Restrepia aristulifera	AF262907	AF265481
Restrepia muscifera	KY988965	KP012449
Restrepiella lueri	KY988966	KY988769
Restrepiella ophiocephala	AF262909	AF265482
Scaphosepalum anchoriferum	KP012459	KP012392
Scaphosepalum grande	AF262819	-
Scaphosepalum jostii	KY988967	KY988770
Scaphosepalum microdactylum	KP012460	KP012393
Scaphosepalum ovulare	KC425764	KP012503
Scaphosepalum swertiifolium	KC425765	KP012504
Scaphosepalum verrucosum	KC425767	KP012505
Specklinia absurda	KC425827	KP012506
Specklinia acoana	KF747800	-
Specklinia acrisepala	KC425768	-
Specklinia alajuelensis	KC425791	-
Specklinia alajuelensis	KC425792	-
Specklinia alajuelensis	KP012454	KP012412
Specklinia alajuelensis	KY988968	KY988771
Specklinia alta	KF747791	KP012394
Specklinia berolinensis	KY988969	-
Specklinia blancoi	KC425772	-
Specklinia brighamii	KC425773	KY988772
Specklinia brighamii	KC425774	-
Specklinia cactantha	KF747795	KP012397
Specklinia calyptrostele	KC425775	KP012507
Specklinia chontalensis	KF747799	KP012399
Specklinia colombiana	KC425809	-
Specklinia corniculata	KF747801	KP012400
Specklinia costaricensis	KC425783	AF265459
Specklinia cucumeris	KF747805	KP012403

Specklinia digitalis	KF747806	KP012404
Specklinia displosa	KF747808	KP012405
Specklinia dodii	KF747809	KP012406
Specklinia dunstervillei	KY988970	KY988773
Specklinia endotrachys	KF747810	KP012407
Specklinia fuegi	KC425786	KP012408
Specklinia fulgens	KC425800	KY988774
Specklinia gersonii	KP012457	KP012424
Specklinia glandulosa	KP012452	KP012410
Specklinia glandulosa	KY988971	KY988775
Specklinia gracillima	KC425793	-
Specklinia grisebachiana	KC425846	-
Specklinia grobyi	KF747813	-
Specklinia grobyi	KF747829	KP012413
Specklinia grobyi	KF747814	-
Specklinia guanacastensis	KP012464	KP012414
Specklinia lanceola	KC425803	KY988776
Specklinia lanceola	KY988972	KY988777
Specklinia lentiginosa	AF275692	-
Specklinia lichenicola	KC425845	-
Specklinia lugduno	KC425824	-
Specklinia luisdiegoi	KC425835	-
Specklinia microphylla	KC425808	-
Specklinia montezumae	KC425811	KP012509
Specklinia morganii	KF747818	KP012415
Specklinia pfavii	KC425814	KP012510
Specklinia picta	KC425815	-
Specklinia pisinna	KC425797	-
Specklinia psichion	KC425816	-
Specklinia recula	KF747822	KP012416
Specklinia remotiflora	KC425819	KP012511
Specklinia schaferi	KC425852	-

Specklinia scolopax	KC425821	KP012512
Specklinia simmleriana	KC425823	-
Specklinia sp.	KY988973	KY988778
Specklinia subpicta	JQ306389	-
Specklinia succulenta	JQ306383	-
Specklinia tribuloides	KC425834	-
Specklinia trichyphis	KC425854	-
Specklinia trilobata	KF747843	-
Specklinia truncicola	JQ306391	-
Specklinia turrialbae	KF747831	KP012425
Specklinia vierlingii	KC425780	-
Specklinia vittariifolia	KP012453	-
Specklinia vittariifolia	KY988974	KY988779
Specklinia wrightii	KC425855	-
Stelis adrianae	JQ995340	-
Stelis alajuelensis	JQ995341	JF934870
Stelis allenii	JQ995342	KY988780
Stelis alta	JF934804	-
Stelis angustipetala	KY988975	-
Stelis argentata	AF262878	-
Stelis argentata	JF934764	-
Stelis argentata	JQ306421	-
Stelis atroviolacea	AF262879	-
Stelis atwoodi	JQ995343	KY988781
Stelis brunnea	JF934798	-
Stelis canae	JQ995362	KY988782
Stelis carnosilabia	JF934808	-
Stelis carnosula	JQ306412	-
Stelis carpinterae	JF934796	JF934858
Stelis carpinterae	JF934796	-
Stelis cobanensis	JQ995344	-
Stelis conochila	KY988976	KY988783

Stelis convallaria	JF934791	JF934851
Stelis cylindrata	JQ995345	KY988784
Stelis deregularis	KC425839	-
Stelis despectans	JF934761	JF934831
Stelis deutroadrianae	JF934799	JF934860
Stelis dimidia	KY988977	KY988785
Stelis dressleri	JF934759	JF934829
Stelis emarginata	JF934781	-
Stelis endresii	JQ306413	-
Stelis ferrelliae	JQ995347	KY988786
Stelis fraterna	JQ306414	-
Stelis galeata	JQ995348	KY988787
Stelis gelida	JF934779	JF934843
Stelis gelida	JF934778	-
Stelis gemma	AF262880	-
Stelis glomerosa	JQ995349	-
Stelis glossula	JF934766	-
Stelis glossula	JF934767	-
Stelis guatemalensis	JF934765	-
Stelis harlingii	EF079364	-
Stelis harlingii	AF262846	AF265465
Stelis hydra	JQ995330	-
Stelis immersa	JF934790	-
Stelis immersa	JF934789	JF934850
Stelis janetiae	JF934802	JF934863
Stelis lamprophylla	JF934774	JF934838
Stelis lamprophylla	JF934776	-
Stelis lanata	AF262881	-
Stelis lankesteri	JQ995353	KY988788
Stelis lennartii	JF934777	JF934841
Stelis lennartii	JQ995324	-
Stelis lueriana	JF934810	JF934870

Stelis lueriana	JF934810	-
Stelis lutea	AF262827	-
Stelis megachlamys	JF934806	JF934867
Stelis megantha	JQ306419	-
Stelis microchila	JF934758	-
Stelis microchila	JF934757	JF934827
Stelis montserratii	JQ995325	-
Stelis montserratii	JQ306493	-
Stelis morae	JF934768	-
Stelis multirostris	JQ995354	KY988789
Stelis mystax	JQ995355	-
Stelis mystax	JF934794	JF934855
Stelis nexipous	KY988978	-
Stelis pachyglossa	JQ995359	KC425865
Stelis papillifera	JF934812	-
Stelis parvula	JQ306420	-
Stelis peliochyla	JQ306417	-
Stelis pidax	KY988979	-
Stelis pileata	JQ995328	KC425861
Stelis pilosa	JF934787	JF934848
Stelis platystylis	JQ995360	-
Stelis pulchella	JF934772	JF934836
Stelis punctulata	JF934783	JF934845
Stelis rodrigoi	AF262829	-
Stelis sclerophylla	JQ995327	-
Stelis segoviensis	AF262866	-
Stelis segoviensis	JQ995367	KY988790
Stelis segoviensis	JQ995365	-
Stelis segoviensis	AF262866	AF276313
Stelis sp.	JQ995326	-
Stelis sp.	KY988980	-
Stelis sp.	KY988981	-

Stelis spnov	JF934770	-
Stelis spnov	JF934763	-
Stelis tubata	AF262845	-
Stelis velaticaulis	AF262847	-
Stelis velaticaulis	JF934773	JF934837
Stelis viridiflava	JQ995330	KC425862
Stelis wercklei	KY988982	-
Stelis wettsteiniana	JQ306422	-
Trichosalpinx arbuscula	KY988983	KY988791
Trichosalpinx berlineri	KY988984	KY988792
Trichosalpinx cedralensis	KY988985	KY988793
Trichosalpinx dependens	JQ306456	-
Trichosalpinx egleri	JQ306347	-
Trichosalpinx fruticosa	KY988986	KY988794
Trichosalpinx minutipetala	KY988987	KY988795
Trichosalpinx nymphalis	KY988988	KY988796
Trichosalpinx orbicularis	KY988989	KY988797
Trichosalpinx pusilla	KY988990	KY988798
Trichosalpinx reflexa	KY988991	KY988799
Trichosalpinx reflexa	KY988992	KY988800
Trichosalpinx sijmii	KY988993	KY988801
Trisetella gemmata	AF262809	-
Trisetella scobina	AF262808	AF265449
Trisetella triglochin	EF079368	EF065592
Trisetella triglochin	AF262807	-
Zootrophion atropurpureum	JQ306415	-
Zootrophion dayanum	AF262898	AF265452
Zootrophion endresianum	KY988994	KY988802
Zootrophion gracillentum	KY988995	-
Zootrophion hirtzii	KY988996	-
Zootrophion hypodiscus	KY988997	-
Zootrophion machaqway	KY988998	KY988803

Zootrophion oblongifolium	KY988999	-
Zootrophion serpentinum	AF262899	-
Zootrophion vulturiceps	KY989000	KY988804
Zootrophion ximenae	KY989001	KY988805

Table S3

Alt.[1] model	Null model	LnL Alt	LnL null	AIC Alt.	AIC Null	AICwt[2] Alt.	AICwt Null	AICwt ratio Alt.	AICwt ratio Null
DEC+J	DEC	-2479	-2495	4965	4994	1	4,70E-07	2139964	4,70E-07
DIVALIKE+J	DIVALIKE	-2584	-2597	5174	5199	1	4,80E-06	208937	4,80E-06
BAYAREALIKE+J	BAYAREALIKE	-2547	-2611	5101	5227	1	4,50E-28	2,21E+27	4,50E-28

[1]Alternative model

[2]AIC weight

Table S4

Alt.[1] model	Null model	LnL Alt	LnL null	AIC Alt.	AIC Null	AICwt[2] Alt.	AICwt Null	AICwt ratio Alt.	AICwt ratio Null
DEC+J	DEC	-1375	-1398	2756	2801	1	2,00E-10	5,10E+09	2,00E-10
DIVALIKE+J	DIVALIKE	-1421	-1432	2848	2868	1	4,20E-05	23705	4,20E-05
BAYAREALIKE+J	BAYAREALIKE	-1418	-1502	2843	3008	1	1,20E-36	8,48E+35	1,20E-36

[1]Alternative model

[2]AIC weight

Table S5

		Denominator models										
	**Shifts**	**0**	**1**	**2**	**3**	**4**	**5**	**6**	**7**	**8**	**9**	**10**
**Numerator models**	**0**	1,000000	0,400000	0,101053	0,019140	0,009213	0,006609	0,010610	0,024198	0,061947	0,357914	0,858993
	**1**	2,500000	1,000000	0,252632	0,047850	0,023033	0,016522	0,026524	0,060495	0,154867	0,894785	2,147484
	**2**	9,895833	3,958333	1,000000	0,189408	0,091172	0,065398	0,104990	0,239458	0,613014	3,541857	8,500456
	**3**	52,246094	20,898438	5,279605	1,000000	0,481350	0,345277	0,554307	1,264246	3,236470	18,699605	44,879053
	**4**	108,540853	43,416341	10,968339	2,077492	1,000000	0,717311	1,151568	2,626462	6,723742	38,848284	93,235883
	**5**	151,316325	60,526530	15,290913	2,896223	1,394096	1,000000	1,605396	3,661538	9,373538	54,158222	129,979733
	**6**	94,254812	37,701925	9,524697	1,804055	0,868381	0,622899	1,000000	2,280769	5,838769	33,735111	80,964267
	**7**	41,325887	16,530355	4,176090	0,790985	0,380740	0,273109	0,438449	1,000000	2,560000	14,791111	35,498667
	**8**	16,142925	6,457170	1,631285	0,308979	0,148727	0,106683	0,171269	0,390625	1,000000	5,777778	13,866667
	**9**	2,793968	1,117587	0,282338	0,053477	0,025741	0,018464	0,029643	0,067608	0,173077	1,000000	2,400000
	**10**	1,164153	0,465661	0,117641	0,022282	0,010725	0,007694	0,012351	0,028170	0,072115	0,416667	1,000000

Table S6

		Denominator models									
	**Shifts**	**0**	**1**	**2**	**3**	**4**	**5**	**6**	**7**	**8**	**9**
**Numerator models**	**0**	1,000000	0,06	0,00281	0,000643	0,0006016	0,00089856	0,00143495	0,0035156	0,00390625	0,0087891
	**1**	15,111	1,000000	0,04255319	0,0097254	0,0090909	0,01357827	0,02168367	0,053125	0,05902778	0,1328125
	**2**	355,111	23,5	1,000000	0,22854691	0,2136364	0,31908946	0,50956633	1,2484375	1,3871	3,1210938
	**3**	1553,77	102,82	4,375	1,000000	0,9347594	1,3961	2,229591	5,4625	6,069444	13,65625
	**4**	1662,22	110	4,6808	1,069	1,000000	1,4936	2,3852	5,84375	6,493055	14,609375
	**5**	1112,8	73,64	3,1339	0,71624714	0,6695187	1,000000	1,59693	3,9125	4,34722	9,78125
	**6**	696,88	46,11	1,962	0,44851259	0,4192513	0,62619808	1,000000	2,45	2,722222	6,125
	**7**	284,44	18,82	0,80100125	0,18306636	0,171123	0,25559105	0,40816327	1,000000	1,11111	2,5
	**8**	256	16,94	0,72090113	0,16475973	0,1540107	0,23003195	0,36734694	0,9	1,000000	2,25
	**9**	113,77	7,52	0,3204005	0,07322654	0,0684492	0,10223642	0,16326531	0,4	0,44444444	1,000000

Table S7

**Models**	**Andean *Cyrtochilum***	**Andean *Oncidium***	**Andean Maxillariinae**	**Andean Zygopetalinae**	**Lowland Catasetinae**
M_1	209,1662181	385,0432344	398,9577432	144,3371826	167,4514812
M_2	209,972848	383,3817387	399,3069314	145,1437381	**172,1239314**
M_3	209,6789014	383,8963526	399,6904126	144,4122827	169,6060185
M_4	**212,3485961**	**385,5664845**	**401,477973**	**147,6038937**	172,0219435

Table S8

**Models**	***Dracula* + *Porroglossum* + *Masdevallia***	***Lepanthes* + *Draconanthes***	***Stelis* + *Pabstiella* + *Pleurothallis***	***Specklinia* (no div. rate shifts)**
M_1	299,0857696	284,272637	626,9890686	412,6994817
M_2	299,7982804	285,6285245	**643,3945671**	406,8332935
M_3	299,7605406	285,576369	627,2068275	**414,8406212**
M_4	**301,6334063**	**287,2096523**	629,3203181	408,5097191

Table S9

Cymbidieae dataset			Pleurothallidinae dataset		
**Models**	**LnL**	**AIC**	**Models**	**LnL**	**AIC**
M_a	-2747,42617	5508,85235	M_a	-1933,82584	3881,65169
**M_b**	**-2747,4254**	**5506,85085**	**M_b**	**-1933,82572**	**3879,65144**
M_c	-2793,1832	5596,36635	M_c	-1935,2922	3880,5844

Table S10

A. *Cyrtochilum*

Models	NP	logL	AICc	ω	λ	α	μ	β
Yule model	1	-84,58	171,25	0,172	0,6569	-	-	-
constant BD model	2	-82,84	169,97	0,326	1,1483	-	0,6987	-
**BAltiVar_EXPO**	**2**	**-82,83**	**169,95**	**0,33**	**0,0067**	**0,0015**	-	-
BAltiVarDcst_EXPO	3	-82,69	171,98	0,119	0,1396	0,0007	0,3189	-
BcstDAltiVar_EXPO	3	-84,6	175,8	0,018	0,6557	-	0,0629	-0,4259
BAltiVarDAltiVar_EXPO	4	-82,72	174,46	0,035	0,1697	0,0005	0,1441	0,0004

B. Andean Maxillariinae

Models	NP	logL	AICc	ω	λ	α	μ	β
Yule model	1	-158,32	318,68	0,296	0,7701	-	-	-
constant BD model	2	-157,51	319,17	0,231	1,0105	-	0,3388	-
**BAltiVar_EXPO**	**2**	**-157,25**	**318,64**	**0,302**	**0,0926**	**0,0008**	-	-
BAltiVarDcst_EXPO	3	-157,22	320,74	0,106	0,2384	0,0006	0,0954	-
BcstDAltiVar_EXPO	3	-158,39	323,07	0,033	0,7686	-	0,0988	-0,5289
BAltiVarDAltiVar_EXPO	4	-157,3	323,11	0,032	0,2159	0,0004	0,1648	-0,023

C. Andean Oncidiinae

Models	NP	logL	AICc	ω	λ	α	μ	β
Yule model	1	-168,88	339,81	0,267	0,6532	-	-	-
constant BD model	2	-168,08	340,3	0,209	0,8447	-	0,2855	-
**BAltiVar_EXPO**	**2**	**-167,58**	**339,31**	**0,343**	**0,0371**	**0,0008**	-	-
BAltiVarDcst_EXPO	3	-167,59	341,47	0,116	0,0775	0,0007	0,046	-
BcstDAltiVar_EXPO	3	-168,94	344,17	0,03	0,652	-	0,0615	-0,4222
BAltiVarDAltiVar_EXPO	4	-167,69	343,87	0,035	0,1452	0,0005	0,2514	-0,0004

D. Andean Zygopetalinae

Models	NP	logL	AICc	ω	λ	α	μ	β
**Yule model**	**1**	**-61,81**	**125,74**	**0,484**	**0,9405**	-	-	-
constant BD model	2	-61,52	127,38	0,213	1,2123	-	0,3832	-
BAltiVar_EXPO	2	-61,71	127,76	0,176	3,1054	0,0003	-	-

BAltiVarDcst_EXPO	3	-61,55	129,81	0,063	1,976	0,0001	0,3017	-
BcstDAltiVar_EXPO	3	-61,85	130,41	0,047	0,9382	-	0,123	-0,6859
BAltiVarDAltiVar_EXPO	4	-61,59	132,39	0,017	0,8809	0,0003	0,696	-1,0391

Abbreviations: NP, number of free parameters; logL, log- likelihood; λ, speciation rate; α, rate of variation of the speciation according to the palaeo-environmental variable; μ, extinction rate; β, rate of variation of the extinction according to the palaeo-environmental variable.

Table S11

A) *Dracula + Porroglossum + Masdevallia*

Models	NP	logL	AICc	ω	λ	α	μ	β
Yule model	1	-158,84	319,72	0	1,1735	-	-	-
**constant BD model**	**2**	**-146,4**	**296,94**	**0,24**	**3,7551**	**-**	**3,1011**	**-**
BAltiVar_EXPO	2	-149,16	302,45	0,015	0,0008	0,0023	-	-
BAltiVarDCST_EXPO	3	-148,3	302,89	0,012	0,0032	0,0026	0,2119	-
BcstAltiVar_EXPO	3	-146,98	300,24	0,046	3,5314	-	2,3458	0,0001
BAltiVarDAAltiVar_EXPO	4	-147,42	303,32	0,009	0,0089	0,0022	0,0608	0,0007

B) *Lepanthes + Draconanthes*

Models	NP	logL	AICc	ω	λ	α	μ	β
Yule model	1	-167,72	337,5	0	1,0588	-	-	-
**constant BD model**	**2**	**-139,57**	**283,3**	**0,397**	**5,8238**	**-**	**5,4409**	**-**
BAltiVar_EXPO	2	-147,8	299,76	0	0,0005	0,0029	-	-
BAltiVarDCST_EXPO	3	-146,25	298,82	0	0,0001	0,0035	0,1602	-
BcstAltiVar_EXPO	3	-141,81	289,94	0,014	5,3559	-	4,4095	0,0001
BAltiVarDAAltiVar_EXPO	4	-145	298,53	0	0,0011	0,0031	0,0462	0,0004

C) *Stelis + Pabstiella + Pleurothallis*

Models	NP	logL	AICc	ω	λ	α	μ	β
Yule model	1	-348,94	699,9	0	1,1983	-	-	-
**constant BD model**	**2**	**-302,3**	**608,67**	**0,377**	**5,348**	**-**	**4,8291**	**-**
BAltiVar_EXPO	2	-316,74	637,54	0	0,0002	0,0027	-	-
BAltiVarDCST_EXPO	3	-314,45	635,04	0	0,0004	0,0031	0,1172	-
BcstAltiVar_EXPO	3	-305,61	617,35	0,005	5,0749	-	3,6911	0,0001
BAltiVarDAAltiVar_EXPO	4	-311,06	630,34	0	0,0041	0,0027	0,0591	0,0008

Abbreviations: NP, number of free parameters; logL, log- likelihood; λ, speciation rate; α, rate of variation of the speciation according to the palaeo-environmental variable; μ, extinction rate; β, rate of variation of the extinction according to the palaeo-environmental variable.
